# Combined angiogenesis and PD-1 inhibition for immunomodulatory TNBC: concept exploration and biomarker analysis in the FUTURE-C-Plus trial

**DOI:** 10.1186/s12943-022-01536-6

**Published:** 2022-03-25

**Authors:** Song-Yang Wu, Ying Xu, Li Chen, Lei Fan, Xiao-Yan Ma, Shen Zhao, Xiao-Qing Song, Xin Hu, Wen-Tao Yang, Wen-Jun Chai, Xiao-Mao Guo, Xi-Zi Chen, Yan-Hui Xu, Xiao-Yu Zhu, Jian-Jun Zou, Zhong-Hua Wang, Yi-Zhou Jiang, Zhi-Ming Shao

**Affiliations:** 1 Key Laboratory of Breast Cancer in Shanghai, Department of Breast Surgery, Fudan University Shanghai Cancer Center, Shanghai, 200032 China; 2 Department of Oncology, Shanghai Medical College, Fudan University, Shanghai, 200032 China; 3 Precision Cancer Medical Center Affiliated to Fudan University Shanghai Cancer Center, Shanghai, 201315 China; 4 Department of Pathology, Fudan University Shanghai Cancer Center, Shanghai, 200032 China; 5 Laboratory Animal Center, Fudan University Shanghai Cancer Center, Shanghai, 201315 China; 6 Department of Radiation Oncology, Fudan University Shanghai Cancer Center, Shanghai, 200032 China; 7 Fudan University Shanghai Cancer Center, Institutes of Biomedical Sciences, Shanghai Medical College of Fudan University, Shanghai, 200032 China; 8 Jiangsu Hengrui Pharmaceuticals Co. Ltd, Shanghai, 201203 China

**Keywords:** Triple-negative breast cancer, Immunomodulatory subtype, First-line treatment, Clinical trial, Combination immunotherapy, Predictive biomarker

## Abstract

**Background:**

Immune checkpoint inhibitors had a great effect in triple-negative breast cancer (TNBC); however, they benefited only a subset of patients, underscoring the need to co-target alternative pathways and select optimal patients. Herein, we investigated patient subpopulations more likely to benefit from immunotherapy and inform more effective combination regimens for TNBC patients.

**Methods:**

We conducted exploratory analyses in the FUSCC cohort to characterize a novel patient selection method and actionable targets for TNBC immunotherapy. We investigated this in vivo and launched a phase 2 trial to assess the clinical value of such criteria and combination regimen. Furthermore, we collected clinicopathological and next-generation sequencing data to illustrate biomarkers for patient outcomes.

**Results:**

CD8-positivity could identify an immunomodulatory subpopulation of TNBCs with higher possibilities to benefit from immunotherapy, and angiogenesis was an actionable target to facilitate checkpoint blockade. We conducted the phase II FUTURE-C-Plus trial to assess the feasibility of combining famitinib (an angiogenesis inhibitor), camrelizumab (a PD-1 monoclonal antibody) and chemotherapy in advanced immunomodulatory TNBC patients. Within 48 enrolled patients, the objective response rate was 81.3% (95% CI, 70.2–92.3), and the median progression-free survival was 13.6 months (95% CI, 8.4–18.8). No treatment-related deaths were reported. Patients with CD8- and/or PD-L1- positive tumors benefit more from this regimen. *PKD1* somatic mutation indicates worse progression-free and overall survival.

**Conclusion:**

This study confirms the efficacy and safety of the triplet regimen in immunomodulatory TNBC and reveals the potential of combining CD8, PD-L1 and somatic mutations to guide clinical decision-making and treatments.

**Trial registration:**

ClinicalTrials.gov: NCT04129996. Registered 11 October 2019.

**Supplementary Information:**

The online version contains supplementary material available at 10.1186/s12943-022-01536-6.

## Background

Immune checkpoint therapies with antibodies targeting the programmed death-1 (PD-1)/PD-1 ligand (PD-L1) axis have gradually become the standard treatment for multiple “immune-hot” tumors, which possess high infiltration of cytotoxic lymphocytes and coexpression of immune-related molecules [1]. Blocking PD-1/PD-L1 has emerged as a breakthrough for systemic treatment of triple-negative breast cancer (TNBC), bringing great efficacy in combination with chemotherapy [2, 3]. Although PD-L1 expression was found to be effective for identifying patients who will benefit from immunotherapy and is widely used in the clinic, this biomarker does not always work [4, 5]. In the phase III KEYNOTE-119 trial, although a late separation of the survival curves and potential benefit in the CPS ≥ 20 subgroup was found, monotherapy with the anti-PD-1 antibody pembrolizumab failed to demonstrate an overall survival (OS) benefit versus chemotherapy [6]. These results suggest that future studies should decipher patient subpopulations more precisely and inform more effective combination regimens for TNBC patients.

TNBC includes molecular subtypes with distinct clinical and biological behaviors [7]. Histologically ‘simple’ TNBCs have been proven to be heterogenous and are clinically and molecularly complex. Among them, the immunomodulatory subpopulation is characterized by elevated immune cell signaling, increased tumor-infiltrating lymphocytes (TILs), and high expression of immune-stimulating and immune-inhibiting molecules, such as PD-L1 [8]. To promote subtype-based precision treatment, we introduced CD8 to define this subgroup of patients [9, 10] and validated its utility in the phase Ib/II FUTURE trial [10]. In brief, for heavily treated CD8-positive immunomodulatory TNBC, 10 (62.5%) out of 16 evaluable patients achieved an objective response at the first postbaseline evaluation under the combination treatment of camrelizumab and nab-paclitaxel [11, 12]. Based on the great efficacy obtained, we aimed to further expand the use of immunomodulatory subtypes and screen novel combinational immunotherapy strategies.

Given the importance of cytotoxic cell infiltration, it is important to elucidate clinically targetable pathways that may hinder CD8^+^ T cell infiltration and function. Numerous studies have aimed to reactivate CD8^+^ T cells [13]. For example, inhibiting glycolytic metabolism may enhance CD8^+^ T cell memory and antitumor function, supporting a potential combination strategy [14]. However, considering the potential toxicity of metabolic inhibitors, there is currently no applicable treatment for the clinic. Triparna Sen et al. targeted the DNA damage response via poly ADP-ribose polymerase and checkpoint kinase 1 inhibition; this strategy activated the STING/TBK1/IRF3 innate immune pathway and induced the activation and function of cytotoxic T cells, which potentiated the antitumor effect of PD-L1 blockade in small-cell lung cancer [15]. Despite the extensive studies attempting to reactivate CD8^+^ T cells, the strategies are limited by possible drug toxicity, low bioavailability, high cost, and so on, thus awaiting further exploration.

In this study, we conducted thorough explorative analyses and confirmed that CD8 positivity could identify an immunomodulatory subpopulation of TNBC, with a higher possibility of benefiting from immunotherapy. We also found that angiogenesis is a key discriminative feature of patients with low CD8^+^ T cell infiltration, and its inhibitor could facilitate checkpoint blockade. Based upon these findings, we launched a proof-of-concept phase II trial to evaluate the efficacy and safety of the novel triplet combination of famitinib, camrelizumab and nab-paclitaxel in advanced immunomodulatory TNBC patients and identified predictive biomarkers to guide further patient selection.

## Materials and methods

### FUSCC-TNBC study cohort and subsequent analysis

This study included a patient cohort from Fudan University Shanghai Cancer Center [7], consisting of 360 primary TNBCs. Informed consent was obtained from patients, and the research protocol was approved by the Clinical Research Ethics Committee of Fudan University Shanghai Cancer Center. Gene set variation analysis (GSVA) was utilized to calculate the enrichment score of each pathway in each sample with use of the “GSVA” package. Fifty pathways in the hallmark gene sets (v7.4) were included, which represent specific well-defined biological states or processes. CD8 staining was performed on FFPE sections [9] and TILs were evaluated on HE sections in a routine diagnostic setting [7]. Molecular subtypes of TNBC were generated as previously described [7].

### Orthotopic tumor model

Six-week-old female BALB/c mice were purchased from Shanghai Jihui Laboratory Animal Care Co., Ltd. and housed in the laboratory animal center of Fudan University Shanghai Cancer Center. For efficacy analysis, 10^5 4T1 cells were injected orthotopically into the fourth mammary fat pad. Anti-PD-1 monoclonal antibody was administered intraperitoneally at 10 mg/kg every six days. Famitinib was given orally at 5 mg/kg daily. Nab-paclitaxel was administered intravenously through the tail vein at 6.5 mg/kg every six days.

The experimental endpoint for individual mice was either maximum diameter of any tumor ≥ 20 mm, indication of necrosis, or death. Primary tumor volumes (mm^3^) were determined using calipers to measure dimensions and calculated using the formula: 0.5 × length x width^2^. At the endpoint, mice were euthanized, followed by surgical excision of tumors and liver and lung tissue. All animal experiments were performed according to protocols approved by the Research Ethical Committee of Fudan University Shanghai Cancer Center (FUSCC-IACUC-2021318).

### Mouse sample assessment

Tissues were quickly excised from mice and cut into 3 parts for RNA extraction, fixation, and flow cytometry. RNA extraction and PCR procedures were carried out according to the manufacturer’s guidelines (Vazyme, Cat#RC101-01, Cat#Q711-03 and Cat#R333-01). The products were quickly transferred to -80 °C for long-term storage. IHC was performed on the FFPE slides, with staining status independently assessed by two experienced pathologists. CD8 (CST, Cat#98941S, 1/200 dilution) and PD-L1 (CST, Cat#64988T, 1/200 dilution) staining scores were calculated according to the number of positive cells per high-power field (HPF, 80X). Granzyme B (Abcam, Cat#ab255598, 1/3000 dilution) and perforin (CST, Cat#31647S, 1/200 dilution) staining scores were calculated by multiplying the staining area score (0–4) and intensity score (0–3) per HPF. Four HPFs were randomly chosen for each slide, and the average score was used. For flow cytometry, tumors were mechanically dissociated and digested with 20 mg/ml dispase II (Roche, Cat#4942078001), 20 mg/ml collagenase I (Sigma, Cat#C0130) and 20 mg/ml hyaluronidase (Sigma, Cat#H3506). Cell suspensions were further filtered through 100-μm and 40-μm strainers and resuspended in PBS, followed by three washes. Red blood cells were lysed with red blood cell lysis buffer (BioLegend, Cat#420301). The cells were then washed in PBS and stained with Zombie-Red™ viability assay (BioLegend, Cat#423110) at a 1/1000 dilution. A monoclonal antibody against CD16/32 (BioLegend, Cat#101319) was used to block cells before staining with antibody panels. After that, cells were stained with fluorescently labeled antibodies against the following surface proteins at a 1/100 dilution in Cell Staining Buffer (BioLegend, Cat#420201): CD45 (Biolegend, Cat#103116), CD3e (Biolegend, Cat#100328), CD4 (Biolegend, Cat#100536), CD8a (BioLegend, Cat#100708), and CD49b (BioLegend, Cat#108922). For intracellular proteins, cells were fixed and permeabilized with fixation buffer (BioLegend, Cat#420801) and intracellular staining permeabilization wash buffer (BioLegend, Cat#421002). Permeabilized cells were then incubated with fluorescently labeled antibodies against perforin (BioLegend, Cat#154404). A CytoFLEX S flow cytometer (Beckman Coulter) and FlowJo software (Version 10.5.3, TreeStar) were used for further analyses.

### FUTURE-C-Plus trial design and procedures

FUTURE-C-PLUS (NCT04129996) was an open-label, single-arm, phase 2 trial conducted in China. Eligible patients included those 18–70 years of age with metastatic or inoperable, locally advanced CD8^+^ TNBC with no prior therapy for such disease. CD8^+^ disease was defined as CD8 expression on at least 10% of cells based on IHC (Ventana, Cat#790-4460) [10]. Confirmation of staining results was preferentially performed with tissue from recurrent or metastatic lesions; otherwise, primary tissue was also acceptable.

Eligible patients received oral famitinib (a tyrosine kinase inhibitor targeting VEGFR-2, PDGFR and c-kit) 20 mg on days 1–28, intravenous camrelizumab (a fully humanized, high-affinity monoclonal antibody against PD-1) 200 mg on days 1 and 15, and intravenous nab-paclitaxel 100 mg/m2 on days 1, 8, and 15 in a 4-week period until disease progression or unacceptable toxicity occurred. Patients received study treatments until disease progression, occurrence of intolerable toxicity, physician decision to stop, or patient withdrawal. If no unacceptable toxicity occurred, nab-paclitaxel was administered for eight cycles. Famitinib dose interruptions and dose reductions (first to 15 mg once daily and subsequently to 15 mg once every other day) were permitted for toxicities that were not relieved by supportive care. Camrelizumab dose reduction was not permitted, but treatment could be delayed (up to 12 weeks) or suspended for the management of an adverse event (AE) (grade 2 or worse severity). The dose of nab-paclitaxel could be reduced to 75 mg/m^2^ or 50 mg/m^2^ and then discontinued if required because of hematological toxicity (grade 2 or worse severity). Camrelizumab or nab-paclitaxel could be independently discontinued in the absence of disease progression. More details on dose modifications are available in the online protocol. For scenarios not specified in the protocol, the investigators were allowed to use discretion in dose modification depending on the severity of toxicity and an assessment of the risk versus benefit for the patient to maximize patient compliance. If the patients left the treatment group, they were treated per clinician choice (if tolerated) and continuously followed up.

The trial protocol (Supplementary Text 1) was approved by the institutional review boards of Fudan University Shanghai Cancer Center (FUSCC), and the trial was conducted in accordance with the Declaration of Helsinki and Good Clinical Practice guidelines. All patients provided written informed consent before enrollment. Patient samples were collected with written informed consent and ethics approval by the FUSCC Ethics Committee.

### Response assessments and end points

The primary endpoint was ORR according to RECIST v1.1, as determined by the investigators. Secondary endpoints included PFS, OS, duration of response (DOR), disease control rate (DCR), safety, tolerability, and biomarker results. Responses were evaluated by investigators using CT or MRI every 8 weeks from baseline for 12 months and then every 12 weeks until disease progression. Patient follow-up was performed every 3 months after treatment discontinuation until death occurred. Safety evaluations, including clinical examination, AE report by patients, and blood count test, were performed on days 1, 8, and 15 of each cycle. Biochemistry tests, electrocardiograms, and echocardiographs were performed on day 1 of every cycle. The severity of AEs was graded based on the National Cancer Institute Common Terminology Criteria for Adverse Events, version 5.0. The causality of AE to study treatment was determined by the investigator.

### Post hoc biomarker assessment

Tumor biopsy and blood DNA were isolated from fresh samples using TGuide M24 (Tiangen, Beijing, China). The purity and quantity of the total DNA were estimated by measuring absorbance at 260 nm (A260) and 280 nm (A280) with a NanoDrop 2000 spectrophotometer (Thermo Scientific, Wilmington, DE, USA); the extracted DNA was considered pure and suitable for future experiments if the A260/A280 ratio was within 1.6–1.9. FUSCC NGS 511-gene panel sequencing [16] was conducted to detect somatic and germline mutations in both biopsy (*n* = 23) and ctDNA (*n* = 23) samples.

FFPE blocks of samples from 30 patients were retrieved, and 4–5-μm-thick samples on slides were used for IHC staining. IHC for TIM-3 (CST, Cat#45208T, 1/400 dilution), c-Myc (Abcam, Cat#ab32072, 1/200 dilution), STING (CST, Cat#13647S, 1/100 dilution), PD-L1 (Abcam, Cat#ab228462, 1/500 dilution) and CD31 (DAKO, Cat#M0823, 1/100 dilution) was performed on FFPE sections from 30 patients, with the staining status being independently assessed by two experienced pathologists. If multiple tissue sections from one patient were available, the highest score was used for classification.

### Statistics

Analyses were performed in R (version 3.6.3) and GraphPad Prism (version 9.0.0). ORRs and 95% CIs were calculated using the Clopper-Pearson method. Median PFS and OS and 95% CI values were calculated using the Kaplan–Meier method. Differential and correlation analyses concerning continuous variables were performed with a nonparametric method. For categorical variables, the chi-square test or Fisher’s exact test was used. For in vivo efficacy studies, two-way ANOVA was used to evaluate significant differences in tumor volumes. All the tests were two-sided, and *P* ≤ 0.05 was considered statistically significant. Unless indicated, data are presented as the mean with corresponding s.e.m. values.

## Results

### CD8 positivity defines immune-enriched tumors in TNBC patients

**Fig. 1:**
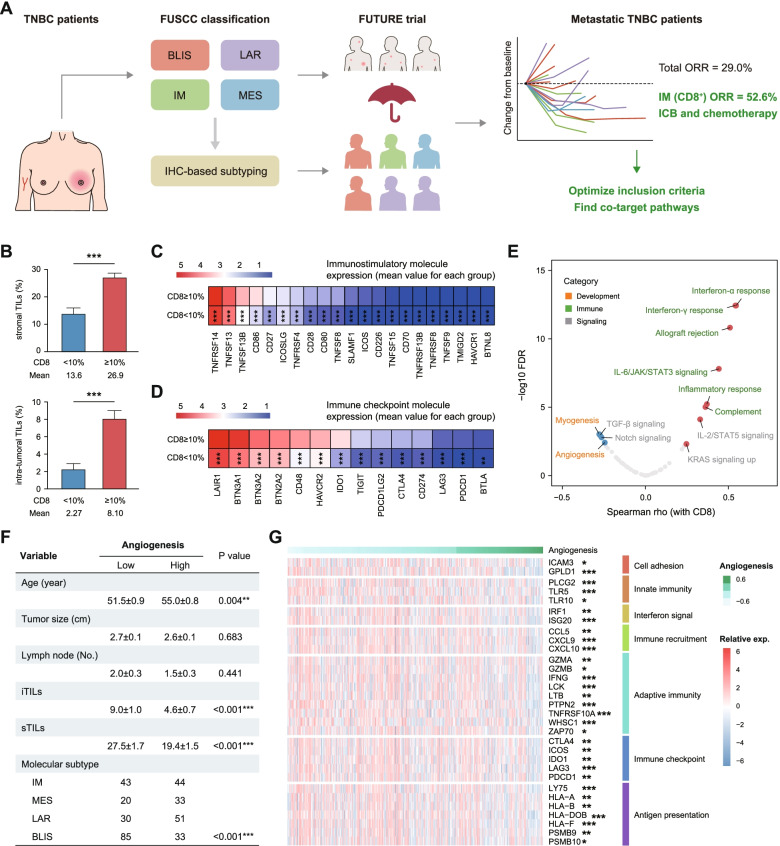
Angiogenesis is targetable in CD8-positive immunomodulatory TNBC patients.** A** Conceptual basis of the study to optimize inclusion criteria and find co-target pathways for effective TNBC immunotherapy. **B** Differences in stromal and intratumoral TILs between the CD8^+^ cells < 10% and CD8^+^ cells ≥ 10% subgroups. **C-D** Differences in immunostimulatory and immune checkpoint molecules between the CD8^+^ cells < 10% and CD8^+^ cells ≥ 10% subgroups. **E** Association of the indicated pathways with CD8^+^ cell infiltration in TNBC patients. Fifty gene sets from the MSigDB hallmark gene sets (v7.4) were included, and symbols with FDR < 0.005 are presented in colors in the plot. **F** Clinical characteristics associated with the angiogenesis enrichment score. **G** Association of representative molecules in different immune-related categories with the angiogenesis enrichment score. TNBC, triple-negative breast cancer; TILs, tumor-infiltrating lymphocytes; BLIS, basal-like immune-suppressed; LAR, luminal androgen receptor; IM, immunomodulatory; MES, mesenchymal-like

Considering the inconsistent efficacy of checkpoint blockade even in PD-L1^+^ tumors, patient selection strategies might need to be refined to improve outcomes. Based on the great efficacy obtained in immunomodulatory TNBC patients in the FUTURE trial [9], we aimed to further optimize the inclusion criteria and screen novel combination strategies for effective TNBC immunotherapy (Fig. 1A).

We first analyzed the distribution of molecular subgroups in patients with different CD8^+^ cell infiltration patterns in the FUSCC-TNBC cohort and found that 94.4% of immunomodulatory tumors were included in the CD8^+^ cells ≥ 10% population. Compared with those in the CD8^+^ cells = 0–9% group, patients in the CD8^+^ cells ≥ 10% group had higher infiltration of both intratumoral and stromal TILs (Fig. 1B). Likewise, patients in the CD8^+^ cells ≥ 10% group had higher expression of immune stimulatory molecules, such as costimulatory signaling molecules (Fig. 1C). Analysis of both clinical and molecular features confirmed that the antitumor response was highly activated in the CD8^+^ cells ≥ 10% group, which also featured enrichment of immune checkpoint molecules, including PDCD1 and CD274 (Fig. 1D), suggesting the potential benefit of PD-1 blockade.

### Targeting angiogenesis is feasible for facilitating CD8^+^ T cell infiltration

To identify actionable targets that may hinder CD8^+^ T cell infiltration, we performed correlative analysis of the CD8 staining score and biological processes. Differential analysis revealed strong enrichment of immune-related pathways, including interferon-alpha response, interferon-gamma response, and inflammatory response, in patients with higher CD8^+^ T cell infiltration compared to those with lower CD8^+^ T cell infiltration (FDR < 0.005) (Fig. 1E). Interestingly, the four negatively correlated pathways included two pathways, myogenesis and angiogenesis, that belong to the development category. Notably, given the clinical accessibility of antiangiogenic drugs, we focused subsequent research on angiogenesis. A higher angiogenesis enrichment score was strongly associated with higher age, lower intratumoral and stromal TILs, and a lower presence of immunomodulatory TNBC patients (Fig. 1F). In addition, correlation analysis revealed that a range of immune-related features were significantly decreased in patients with higher angiogenesis signature scores (Fig. 1G), which further supported the use of antiangiogenic therapy to facilitate checkpoint blockade.

### Activity and safety of famitinib plus PD-1 blockade-based therapy in vivo

**Fig. 2:**
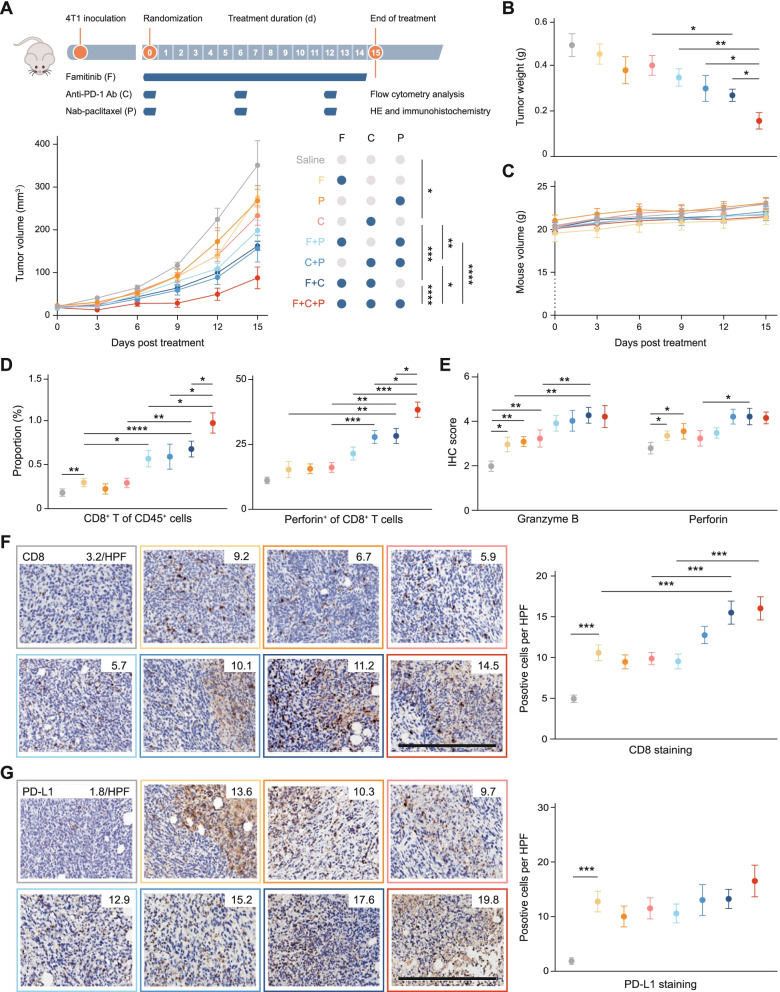
Famitinib facilitates PD-1 blockade to enhance antitumor immunity in a safe manner in vivo.** A** Study design and tumor growth curve in each treatment group. (*n* = 10 per group). **B-C** Tumor weight on the final day and mouse weight during treatment of each group as described above. **D** Proportion of CD8^+^ T cells among CD45^+^ cells and perforin^+^ cells among CD8^+^ T cells as assessed by flow cytometry. **E** Granzyme B and perforin expression as quantified by immunohistochemistry. **F-G** CD8^+^ and PD-L1^+^ cells as quantified by immunohistochemistry. Representative images of CD8 and PD-L1 staining are shown, with the mean cell number per HPF (800X) indicated. HPF, high-power field. Scale bars, 100 μm

To further evaluate whether angiogenesis inhibition facilitates immune checkpoint inhibitor-based therapy, we evaluated the activity and safety of the triplet combination of famitinib (an anti-angiogenetic tyrosine kinase inhibitor), PD-1 blockade and nab-paclitaxel (Fig. 2A). This triplet combination showed the best therapy response compared with other therapies (Fig. 2A-B and Fig. S1A-B) with a safe profile (Fig. 2C and Fig. S1C-D). In addition, the triplet regimen induced a pronounced immune response, with increased infiltration of CD8^+^ T cells and perforin production by cytotoxic T lymphocytes (Fig. 2D and Fig. S1E). This response was associated with a marked, synergistic increase in infiltrating lymphocytes and cytotoxic molecule expression (Fig. 2E-F and Fig. S1F-G). In addition, famitinib alone had a great immunostimulatory effect in vivo (Fig. 2D-F) and induced elevated expression of PD-L1 (Fig. 2G), indicating the validity of combining it with PD-1 blockade. Interestingly, treatment of mice bearing disease with combination angiogenesis inhibition and anti-PD-1 therapy had similar, although not as strong, therapeutic, and immune-stimulating efficacy to the triplet combination and even showed equivalent efficacy with an anti-PD-1 antibody and nab-paclitaxel combination, warranting investigation of the chemo-free regimen.

### Efficacy analysis of the triplet regimen and stratification based on CD8 status in patients in the FUTURE-C-Plus trial

**Fig. 3:**
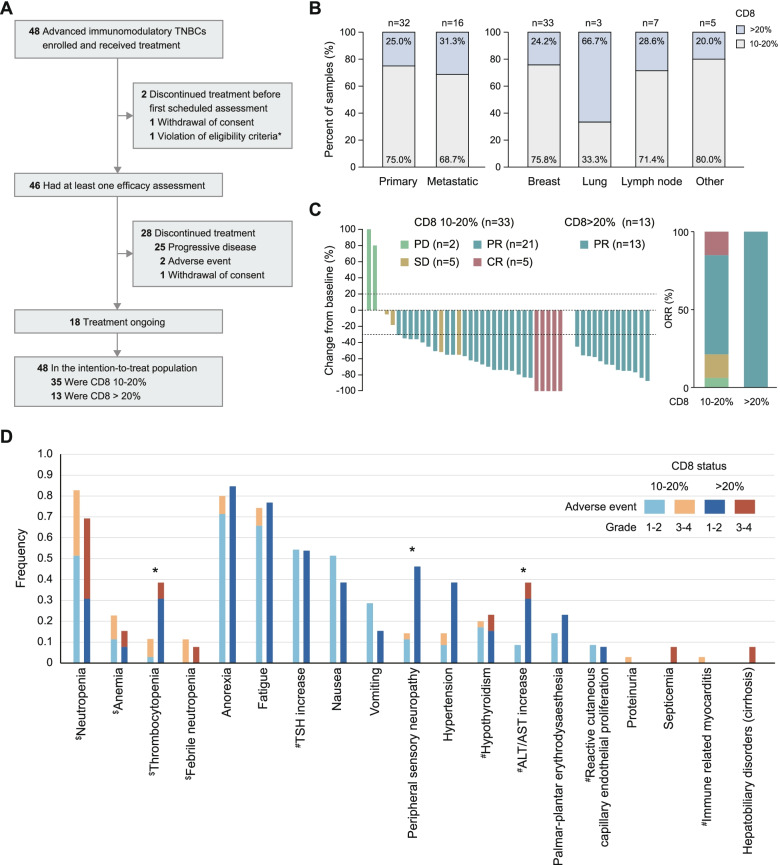
Efficacy and safety profiles of the triplet regimen in patients grouped by CD8 status. **A** Profile of the FUTURE-C-Plus trial. **B** Infiltration of CD8^+^ cells based on tumor source (primary or metastatic) and anatomical location. **C** Best percentage change from baseline in the target lesion in patients grouped by CD8 status. **D** Frequency of adverse events in patients grouped by CD8 status. All patients (*n* = 48) were included in the safety analysis. One case of ALT/AST increase was defined as potentially immune-related. $ Hematological toxicity; # potential immune-related toxicity. TSH, thyroid-stimulating hormone; ALT, alanine aminotransferase; AST, aspartate aminotransferase

Then, we launched a proof-of-concept phase II trial to validate the above established patient selection criteria and triplet regimen. Between October 2019 and October 2020, 48 (out of 122 screened) advanced immunomodulatory (CD8^+^ cells ≥ 10%) TNBC patients were enrolled and received the triplet combination of famitinib, camrelizumab, and nab-paclitaxel every 4 weeks (Table 1). Thirty-two (66.7%) patients had received previous chemotherapy in the (neo)adjuvant and/or metastatic setting. At data cutoff (September 30, 2021), the median follow-up was 17.0 months (range, 8.7–24.3), and 46 (95.8%) patients with at least one postbaseline assessment were eligible for efficacy analysis (Fig. 3A). Two (4%) patients discontinued treatment before the first scheduled postbaseline scan due to a second primary tumor (*n* = 1) and withdrawal of consent (*n* = 1).

**Table 1:** Baseline characteristics grouped by CD8 status (*N*=48)

**Characteristics**	**All**	**10-20% (*n*=35)**	**> 20% (*n*=13)**
Age, years			
Median (IQR)	50 (39-60)	49 (38-58)	52 (46-60)
< 40	12 (25.0)	10 (28.6)	2 (15.4)
≥ 40	36 (75.0)	25 (71.4)	11 (84.6)
Disease status			
Metastatic, de novo	16 (33.3)	9 (25.7)	7 (53.8)
Metastatic, recurrent	31 (64.6)	26 (74.3)	5 (38.5)
TFI 6-12 months	15 (31.3)	12 (34.3)	3 (23.1)
TFI >12 months	16 (33.3)	14 (37.1)	2 (15.4)
Locally inoperable advanced	1 (2.1)	0 (0)	1 (7.7)
ECOG performance status			
0	18 (37.5)	11 (31.4)	7 (53.8)
1	30 (62.5)	24 (68.6)	6 (46.2)
Baseline disease			
0	15 (31.2)	8 (22.9)	7 (53.8)
1	31 (64.6)	25 (71.4)	6 (46.2)
NA	2 (4.2)	2 (5.7)	0 (0)
Number of metastatic sites			
< 3	25 (52.1)	18 (51.4)	7 (53.8)
≥ 3	23 (47.9)	17 (48.6)	6 (46.2)
Metastatic site			
Lung	24 (50.0)	19 (54.3)	5 (38.5)
Liver	10 (20.8)	8 (22.9)	2 (15.4)
Bone	19 (39.6)	16 (45.7)	3 (23.1)
Neo/adjuvant chemotherapy			
Any	32 (66.7)	26 (74.3)	6 (46.2)
Anthracycline	30 (62.5)	24 (68.6)	6 (46.2)
Taxane	29 (60.4)	23 (65.7)	6 (46.2)
Platinum	6 (12.5)	6 (17.1)	0 (0)
Capecitabine	5 (10.4)	3 (8.6)	2 (15.4)
PD-L1 status			
Positive	17 (35.4)	9 (25.7)	8 (61.5)
Negative	13 (27.1)	10 (28.6)	3 (23.1)
Unknown	18 (37.5)	16 (45.7)	2 (15.4)

Data are presented as No. (%) unless otherwise indicated

*Abbreviation:* *ECOG* Eastern Cooperative Oncology Group, *TFI* treatment-free interval, *PD-L1* programmed death-ligand 1

Of the 48 annotated samples, 32 (66.7%) tissue samples were collected from primary tumors and 16 (33.3%) were collected from metastases (Fig. 3B); the ratio was similar to that of the IMpassion 130 trial [17]. The rate of samples with CD8^+^ cells > 20% was slightly higher in metastatic tumor samples (31.3% [5 of 16 samples]) than in primary tumor samples (25.0% [8 of 32 samples]). In addition, the rate of samples with CD8^+^ cells > 20% varied by anatomical location, with lung tissues having the highest rate (66.7%) and other tissues having similar rates.

The ORR was the primary endpoint of the FUTURE-C-Plus trial, and 81.3% patients in the intention to treat (ITT) population achieved objective response (Table 2). All 13 patients with CD8^+^ cells > 20% achieved an objective response, while 26 (74.3%) of 35 patients with CD8^+^ cells = 10–20% achieved an objective response (Fig. 3C). Among the 46 patients with post-baseline tumor assessment, the median duration of treatment was similar in the CD8^+^ cell > 20% and CD8^+^ cell = 10–20% subgroups (10.0 and 9.8 months, respectively) (Fig. S2). Six (46.2%) and 12 (34.3%) patients in each subgroup continued treatment up to the data cutoff.

**Table 2:** Antitumor activity grouped by CD8 status (*N*=48)

Antitumor activity	All	10-20% (*n*=35)	> 20% (*n*=13)
Objective response	39 (81.3, 70.2-92.3)	26 (74.3, 56.7-87.5)	13 (100, 75.3-100)
Best overall response			
Complete response	5 (10.4)	5 (14.3)	0 (0)
Partial response	34 (70.8)	21 (60.0)	13 (100)
Stable disease	5 (10.4)	5 (14.3)	0 (0)
Progressive disease	2 (4.2)	2 (5.7)	0 (0)
Unknown	2 (4.2)*	2 (5.7)	0 (0)

Data are presented as No. (%, 95% CI) or No. (%)

Responses were assessed in accordance with RECIST version 1.1. Only confirmed responses were included

*Two patients exempted post-baseline efficacy assessments

Of the 48 enrolled patients, 46 (95.8%) had at least one treatment-related adverse event (AE), most of which were anorexia (81.3%), neutropenia (79.2%), and fatigue (75.0%), and 24 (50%) had grade ≥ 3 AEs (Table S1). AEs that led to the discontinuation of any agent occurred in 3 (6.3%) patients. No treatment-related deaths were reported. Overall, the rates of thrombocytopenia (38.5% vs. 11.4%, *P* = 0.048), peripheral sensory neuropathy (46.2% vs. 14.3%, *P* = 0.048), and ALT/AST increase (38.5% vs. 8.6%, *P* = 0.025) were higher in CD8^+^ cell > 20% patients (Fig. 3D).

### Baseline clinico-genomic parameters associated with ORR

**Fig. 4:**
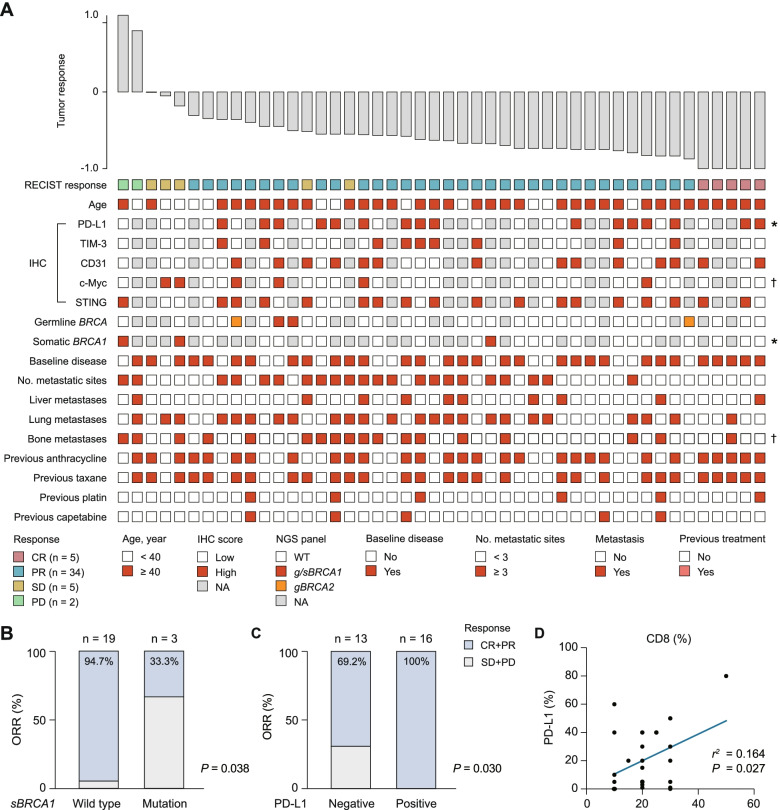
Baseline biomarker results from clinico-genomic data based on RECIST response. **A** Patient age, IHC results, germline (according to routine clinical diagnostic testing) and somatic *BRCA* mutation status, clinical characteristics, and prior therapies are depicted. Data were available for 46 patients with at least one postbaseline efficacy assessment. Samples were taken at baseline before study treatment. **B-C** ORR grouped by somatic *BRCA1* mutation and PD-L1 expression. **D** Correlation between CD8 status and PD-L1 expression. IHC, immunohistochemistry; ORR, objective response rate; NA, not available

To identify predictive biomarkers for the triplet regimen, we first focused on the association between commonly used clinicopathological parameters, including patient age, immunohistochemistry (IHC) results, *BRCA* mutation status, metastatic disease status, and previous treatment, of enrolled patients and therapy response in terms of ORR (Fig. 4A). All patients with samples included in these analyses had a confirmed response. Interestingly, we found that the presence of *BRCA1* somatic mutation indicated a worse tumor response (*P* = 0.038, Fig. 4B). All patients with PD-L1^+^ tumors achieved an objective response, while only 69.2% of patients with PD-L1^−^ tumors achieved an objective response (*P* = 0.030, Fig. 4C). Next, we found a positive correlation between PD-L1 expression (stained by sp142) and CD8 status (*P* = 0.027, Fig. 4D), further suggesting that CD8^+^ T cells could be incorporated as a biomarker into established PD-L1-based patient selection criteria.

### Baseline biomarker results from biopsy specimens based on survival

**Fig. 5:**
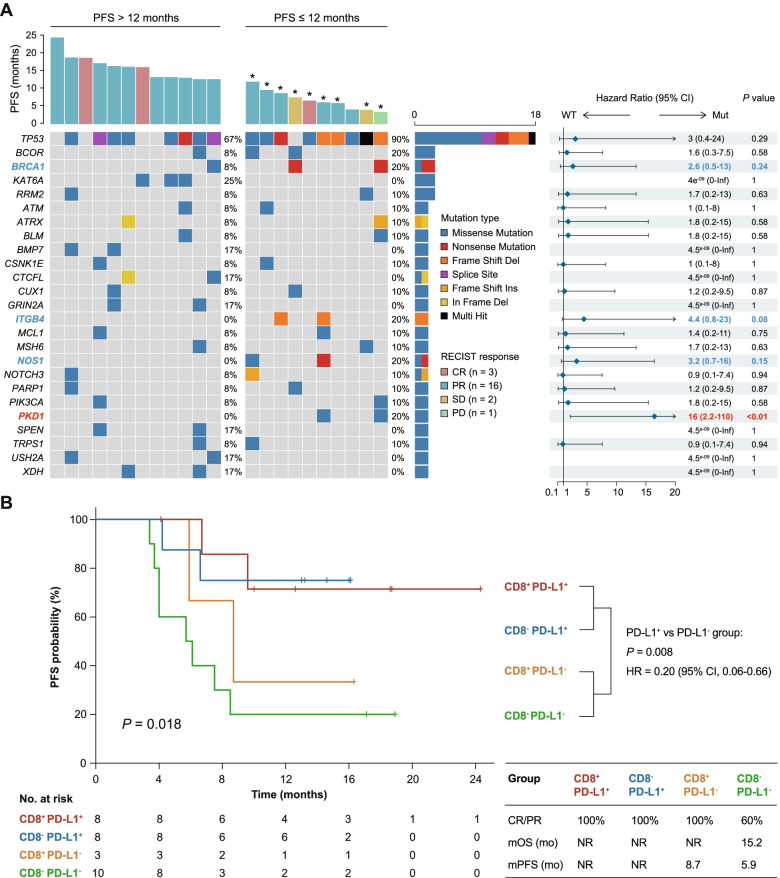
Baseline biomarker results from biopsy specimens based on PFS. A Genomic events based on timing of progression following treatment (PFS event ≤ 12 versus > 12 months); asterisks indicate censoring. An exploratory forest-plot analysis of PFS according to specific somatic mutations showing unstratified hazard ratios with 95% CIs for progression. **B** Kaplan–Meier estimates of PFS by CD8 status and PD-L1 expression. PFS was assessed in patients with available PD-L1 staining results (*n* = 29). CD8 > 20% tumors were defined as CD8^+^. PFS, progression-free survival

Progression-free survival (PFS) and overall survival (OS) are important secondary endpoints. The median PFS for this trial was 13.6 months (95% CI, 8.4–18.8), and the median OS was not reached. We used the Kaplan–Meier method to evaluate the predictive value of frequent somatic mutations (≥ 5% in the whole cohort). All patients with samples included in these analyses had > 12 months of follow-up, except for one recently enrolled patient. To summarize, somatic mutation of *PKD1* indicated poor PFS (*P* < 0.01, Fig. 5A). In addition, mutated *BRCA1*, *ITGB4* and *NOS1* also indicated a trend of worse response. Such results were partly validated with the use of OS data (Fig. S3). *PKD1* somatic mutation also indicated poor OS (*P* = 0.01), and a similar trend was observed for mutation of *BLM*, *MSH6* and *NOS1*.

We next sought to determine whether PD-L1 and CD8 were related to survival. A classification system based on the two markers showed a survival difference in terms of PFS (*P* = 0.018, Fig. 5B). Patients with CD8^+^PD-L1^+^ or CD8^−^PD-L1^+^ tumors had the best PFS, with a median PFS not reached, compared to 8.7 months in CD8^+^PD-L1^−^ and 5.9 months in CD8^−^PD-L1^−^ tumors (Fig. 5B). For ORR and OS, CD8^−^PD-L1^−^ tumors showed the worst values (ORR = 60% and median OS = 15.2 months), further suggesting that CD8 can be considered with PD-L1 staining to define patients who are most likely to benefit from combination immunotherapy. A pairwise comparison of PFS between patients with PD-L1^+^ and PD-L1^−^ tumors also showed a highly significant difference in PFS outcome (*P* = 0.008). Patients with CD8^+^ tumors had a better PFS than those with CD8^−^ tumors, although this difference did not reach statistical significance (*P* = 0.16).

These data suggested the value of somatic mutations together with classic PD-L1 and CD8 staining for predicting the outcome of combination immunotherapy in advanced immunomodulatory TNBC patients and further highlighted the need to co-target related pathways in future treatment strategies.

## Discussion

With the substantial increase in emergent technologies in the realm of tumor immunology, the improved understanding of tumor-immune interactions contributes to the development of immune-oncology and yields potential strategies in TNBC treatment. However, the benefit of current treatment strategies is still far from satisfactory, and barriers to precision treatment still exist [18]. Although TNBC seems to be relatively “hotter” than other types of breast cancer, it demonstrates a relatively poor efficacy of immunotherapy compared with other “immune-hot” tumors, such as melanoma [6, 19]. Provided that TNBC is a highly clinically and biologically heterogeneous disease, there is an urgent need to subgroup TNBC patients to identify those who are more likely to benefit from immunotherapy and to find a clinically actionable method to further remodel the “immune-hot” tumor microenvironment (producing not only higher immune infiltration but also better antitumor immunity) [20].

Here, we found that CD8 status was able to identify an immune-enriched subpopulation in TNBC with a higher possibility of benefiting from immunotherapy. Further integrative analysis demonstrated that angiogenesis is a key discriminative feature of patients with low CD8^+^ T cell infiltration and can represent an immunosuppressive phenotype. In FUTURE-C-Plus trial, the well-selected CD8-positive population of advanced TNBC patients responded well and safely to combined famitinib, camrelizumab and nab-paclitaxel.

To date, numerous biomarkers have been proven to be associated with immunotherapy efficacy, typified by PD-L1 expression, tumor mutation burden, and TILs [21, 22]. Among these, PD-L1 expression was effective in identifying patients who are likely to benefit from immunotherapy and is widely used in the clinic [2, 3, 17, 23]. In the future, seeking novel, well-validated biomarkers to complement PD-L1 expression in TNBC is of high clinical significance. Since immune checkpoint inhibitors block PD-1/PD-L1 inhibitory signals on cytotoxic T cells to induce subsequent clinical benefit [24, 25], it is not strange that successful treatment strategies require sufficient T cells in the tumor bed [4, 26]. Based on previous studies, we explored the clinical value of the CD8 score as a biomarker to identify the immunomodulatory subpopulation. The proof-of-concept FUTURE-C-Plus trial, which enrolled patients with ≥ 10% CD8^+^ cells, validated the utility of this strategy by virtue of the impressive ORR achieved in advanced TNBC patients. To better verify the role of the CD8 staining score, we divided patients into two groups using a CD8^+^ cells > 20% cutoff. The CD8^+^ cells > 20% group exhibited better ORR, PFS and OS. Adverse events, especially thrombocytopenia, peripheral sensory neuropathy and ALT/AST increase, were more frequent in these patients, which could indicate a powerful immune response and should be treated in advance, such as cytokine storm [27]. This result supports the use of CD8 in the clinic to perform clinically meaningful patient stratification.

In addition, the combination of angiogenesis inhibition and checkpoint blockade demonstrates increased efficacy. The crosstalk between tumor angiogenesis and immune cells is more likely to be a destructive cycle in growing tumors [28]. The presence of tumor vessels was reported to hamper CD8^+^ T cell trafficking into the tumor bed and inhibit their cytotoxicity [29, 30]. Moreover, VEGF, the pivotal driver of angiogenesis, interferes with the maturation of dendritic cells, thereby suppressing T cell priming, and induces exhaustion of CD8^+^ T cells [31]. Meanwhile, a variety of innate and adaptive immune cells contribute to the malformation of tumor vessels. For example, M2-polarized macrophages and regulatory T cells can secrete pro-angiogenic factors that promote vascular immaturity. While CD8^+^ T cells could suppress angiogenesis and induce vascular maturation by secreting IFN-γ [31]. These findings indicated that normalizing aberrant vascular-immune crosstalk could potentiate cancer immunotherapy [28, 32, 33]. Furthermore, this combination strategy has been demonstrated through pivotal clinical trials, granted approval from the FDA, and is now being used in patients with kidney or lung cancer [34, 35]. Although it has been explored in a series of clinical trials, the basic mechanism underlying the success of the combination of antiangiogenic therapy and immunotherapy remains unclear [36]. In the FUSCC-TNBC cohort, we found that angiogenesis was inversely correlated with CD8^+^ cell infiltration, which indicates that angiogenesis has the potential to be targetable in CD8^+^ TNBC. The orthotopic tumor model suggests that such regimen strongly promotes both CD8^+^ T-cell infiltration and function compared with a single agent or doublet therapy and maintains biological safety. The patient-level data further validated this hypothesis. The mechanism underlying the success of the combination therapy is likely attributed to the inhibition of neovasculature and the generation of normal vessels. The great result obtained in FUTURE-C-Plus trial may also be attributable to the ethnic factors; thus, further international multi-center studies are needed.

Notably, we found that the effects of the famitinib and anti-PD-1 antibody combination were close to those of the triplet regimen in vivo. As reported in the in vivo experiments, angiogenesis inhibition alone effectively increased infiltration of cytotoxic T cells and PD-L1 expression to remodel the tumor immune microenvironment and sensitive immunotherapy. Together with the predictable reduction in adverse effects, the combinational chemo-free regimen is worthy of the validation in the future.

Furthermore, we revealed clinicopathologic and genomic predictive biomarkers. As expected, all PD-L1^+^ patients achieved an objective response [2, 23, 37]. CD8^+^ T cells can be used in combination with PD-L1 expression to further identify patients who are more likely to benefit in all aspects. Although the PFS between CD8 10–20% and CD8 > 20% patients did not reach statistical significance, this might be due to the lack of CD8 < 10% patients, so the predictive value of CD8 could be underestimated. In contrast, *PKD1* somatic mutation indicated both worse PFS and OS. *PKD1* encodes a member of the polycystic protein family and is considered associated with polycystic kidney disease [38]. Previous studies have shown that *PKD1* can regulate various biological processes, including cell proliferation, survival, motility, and so on, and ultimately alter cancer cell behaviors [39]. Mutations in the *PKD1* gene could also result in autosomal dominant polycystic kidney disease and loss of PKD1 impaired lysosomal activity in a calpain-dependent manner [40]. However, its functional role in cancer immunotherapy was not mentioned and should be further elucidated. We also found that some gene mutations, such as those in *BRCA1*, *ITGB4* and *NOS1*, tended to predict less benefit from the treatment. In addition, the non-mutated genes in less responsive patients should also be explored. Larger patient cohorts and detailed basic experiments are awaited to further verify the predictive value of these gene events and their underlying biological meaning in immunotherapy.

Considering that FUTURE-C-Plus is a single-arm clinical trial and lacks CD8 < 10% patients, more convincing evidence is needed, and subsequent randomized controlled FUTURE-SUPER trials are awaited. Although the basic mechanism by which combined antiangiogenic therapy and immunotherapy provide impressive benefits was partly elucidated with an in vivo model, the full mechanism remains unclear. In addition, it will be informative to explore the change in the immune milieu after angiogenic inhibitor treatment, which has the potential to identify patients who are most likely to respond. Nevertheless, the same limitations that apply to most types of predictive biomarker studies also apply to this study: the results are often based on single biopsies collected at a single time point, and cumulative data collection is lacking. The fact that tumors and their responses to therapy are often heterogeneous and vary with the therapy duration can cause unreliable results. In future studies, it will be necessary to collect appropriate biological samples, such as baseline tumor biopsies and serial blood samples every two treatment cycles, for translational analysis.

## Conclusion

 In summary (Fig. S4), with prior preclinical exploratory analyses and in vivo experiments, our FUTURE-C-Plus study confirmed the feasibility of a precision subtype-guided combination strategy in advanced TNBC patients. Antiangiogenic agents have their own role and act in concert with anti-PD-1 antibody and chemotherapy regimens. Both clinicopathological and genomic potential predictive biomarkers were identified and warrant further evaluation in the ongoing randomized controlled trial FUTURE-SUPER. Overall, our findings lay the foundation for a strategy to stratify patients based on biomarkers to identify those who are more likely to benefit from immunotherapies and suggest the potential of combining angiogenesis inhibition, checkpoint blockade and chemotherapy for treating TNBC patients; such a strategy may be broadly applicable in other malignancies.

## Supplementary Information

**Additional file 1.** 

## Data Availability

All data needed to evaluate the conclusions in the paper are presented in the paper and/or the Supplementary Materials or can be asked via the correspondence (ZS, zhimingshao@fudan.edu.cn) upon reasonable request. Microarray data and sequence data from the FUSCCTNBC cohort have also been deposited in the Sequence Read Archive (WES and RNA-seq; SRA: SRP157974).

## Abbreviations

AE: Adverse event
CR: Complete response
ctDNA: Circulating tumor DNA
DCR: Disease control rate
DOR: Duration of response
FFPE: Formalin-fixed, paraffin-embedded
GSVA: Gene set variation analysis
ICB: Immune checkpoint blockade
IHC: Immunohistochemistry
ITT: Intention to treat
NGS: Next-generation sequencing
ORR: Objective response rate
OS: Overall survival
PD-1: Programmed death-1
PD-L1: Programmed death-1 ligand
PD: Progressive disease
PFS: Progression-free survival
PP: Per-protocol
PR: Partial response
SD: Stable disease
TIL: Tumor-infiltrating lymphocyte
TNBC: Triple-negative breast cancer
VEGF: Vascular endothelial growth factor
