# Supplementary material for: Combined angiogenesis and PD-1 inhibition for immunomodulatory TNBC: concept exploration and biomarker analysis in the FUTURE-C-Plus trial
Source: Mol Cancer. 2022 Mar 25;21:84. doi: 10.1186/s12943-022-01536-6 (PMC8951705; doi:10.1186/s12943-022-01536-6)
Supplement: Supplementary file 1 — Additional file 1. [file 12943_2022_1536_MOESM1_ESM.pdf]

Supplementary Materials

This PDF file includes:

Supplementary Figure 1-4, Supplementary Table 1, Supplementary Text 1

Supplementary Figure 1. The triplet regimen is safe and immunogenic.

Supplementary Figure 2. Duration of therapy grouped by CD8 status.

Supplementary Figure 3. Baseline biomarker results from biopsy specimens based on OS.

Supplementary Figure 4. Schematic diagram of the study.

Supplementary Table 1. Treatment-related adverse events grouped by CD8 status (N=48).

Supplementary Text 1. Trial protocol.

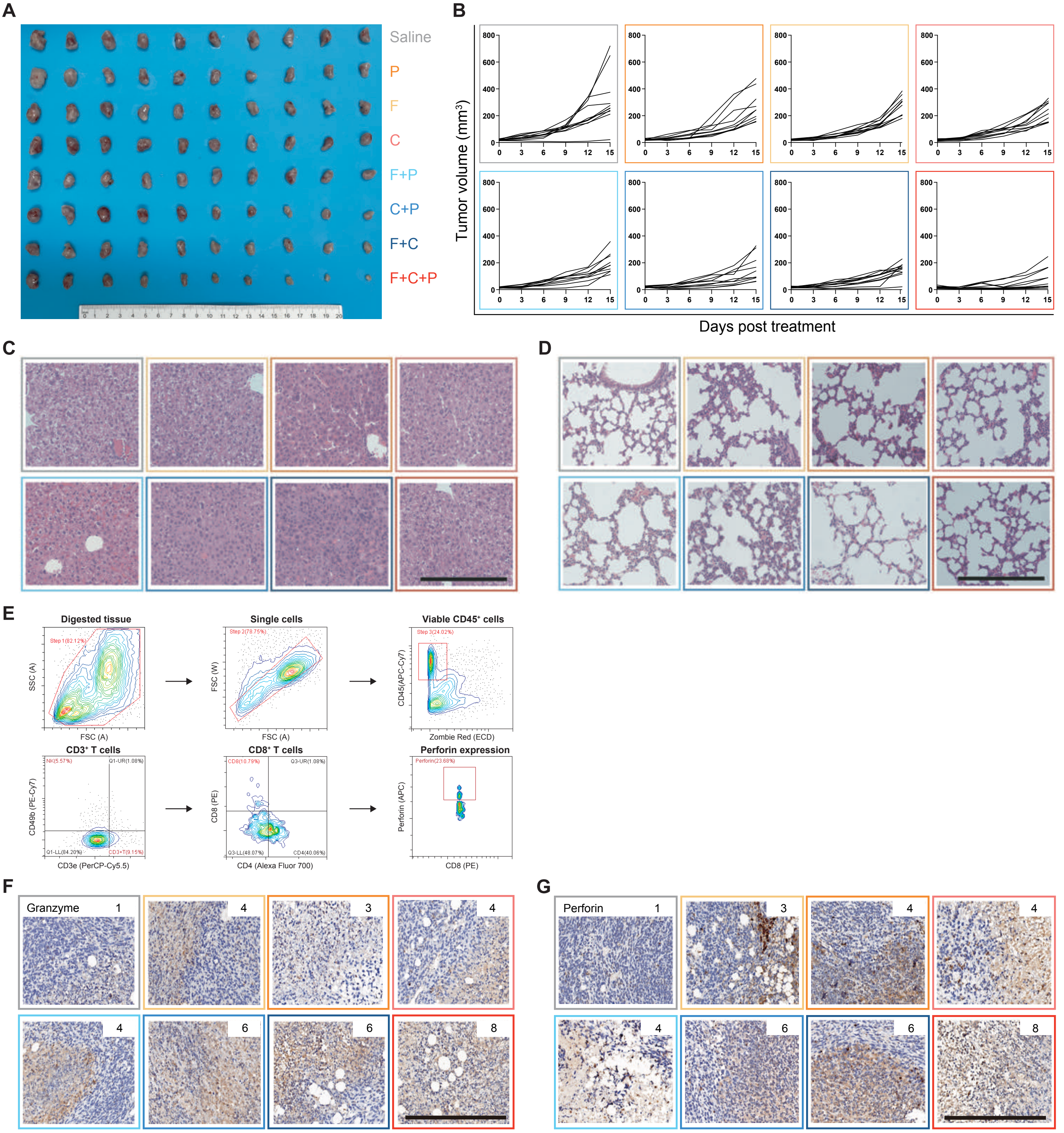

Supplementary Figure 1. The triplet regimen is safe and immunogenic.

(A) Representative images of the subcutaneous breast tumors in each group.

(B) Individual tumor growth curves in each treatment group.

(C-D) Safety evaluation with use of liver (**C**) and lung (**D**) sections.

(E) Gating strategy of Perforin^{+}CD8^{+} T cells in mice.

(F-G) Representative images of granzyme (**F**) and perforin (**G**) in each group. Scale bars, 100 μm.

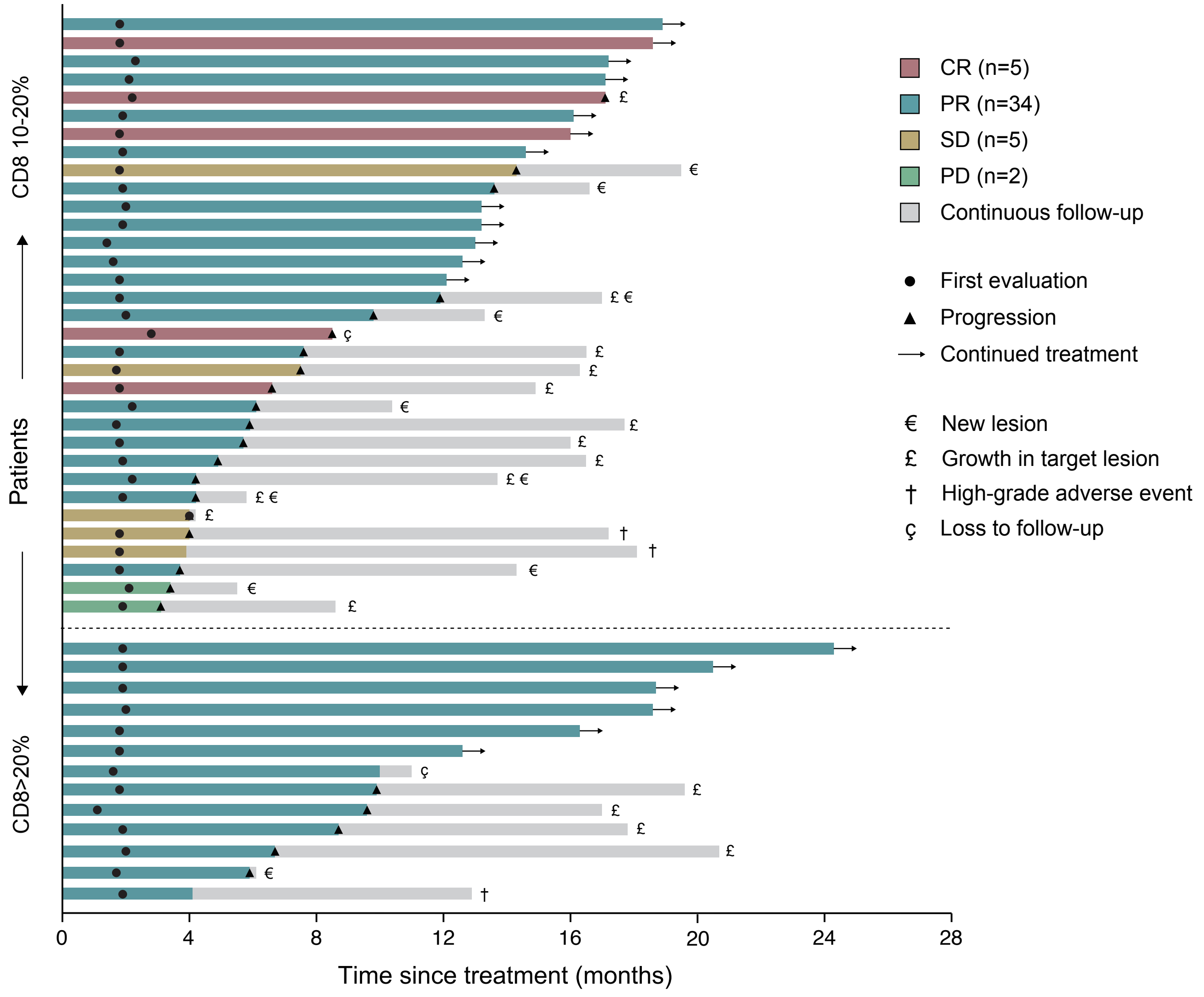

Supplementary Figure 2. Duration of therapy grouped by CD8 status.

Duration of therapy in patients with post-baseline assessment data (n=46); the colorful bar length represents the treatment duration of each patient, and the grey bar length represents the follow-up time after therapy finished.

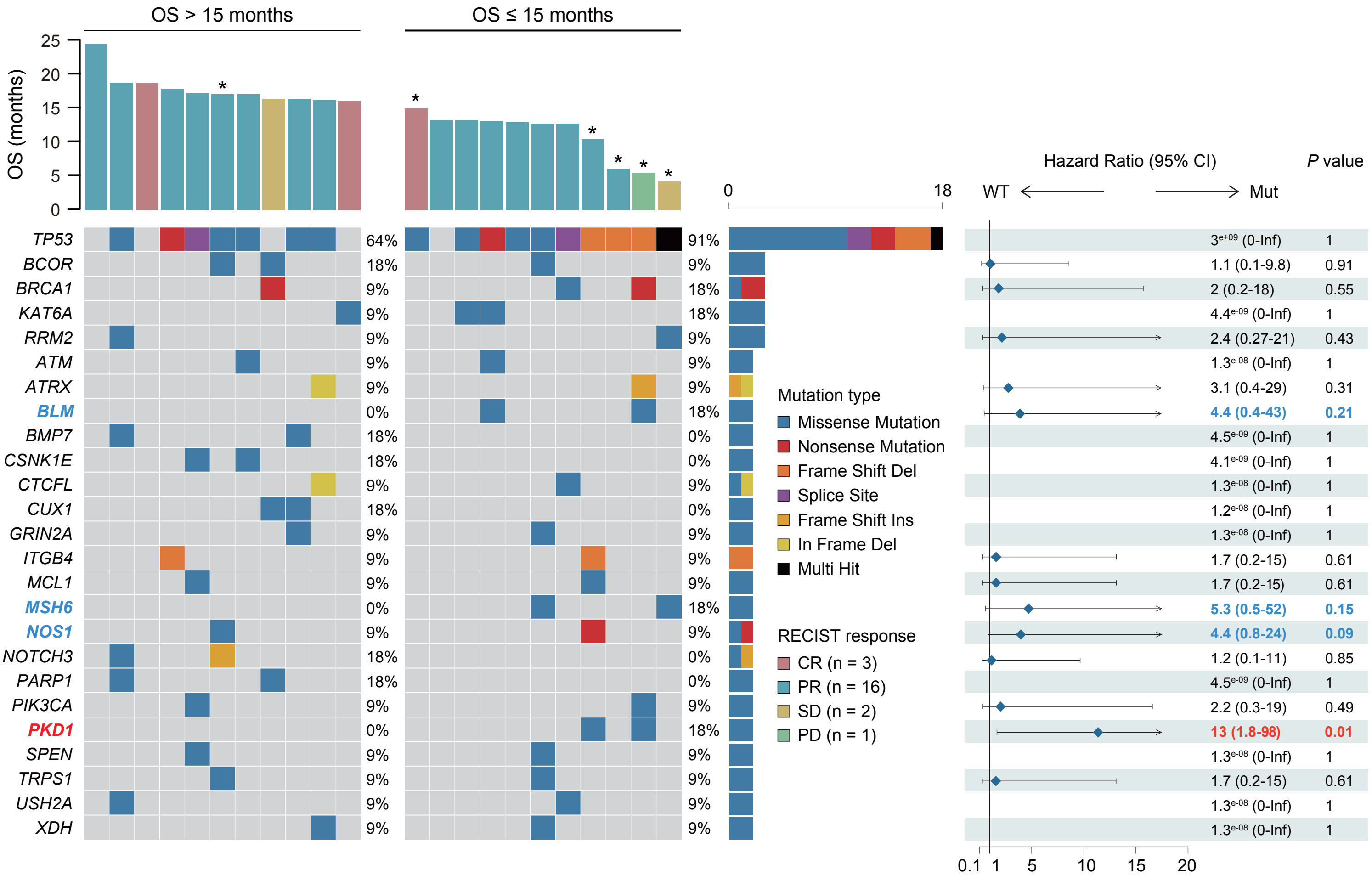

Supplementary Figure 3. Baseline biomarker results from biopsy specimens based on OS.

Left panel: Genomic events of patients based on timing of death following treatment (OS events ≤15 versus >15 months); asterisks indicate censoring. Right panel: An exploratory forest-plot analysis of OS according to specific somatic mutation. Unstratified hazard ratios with 95% CIs for death are shown. OS, overall survival; CI, confidence interval.

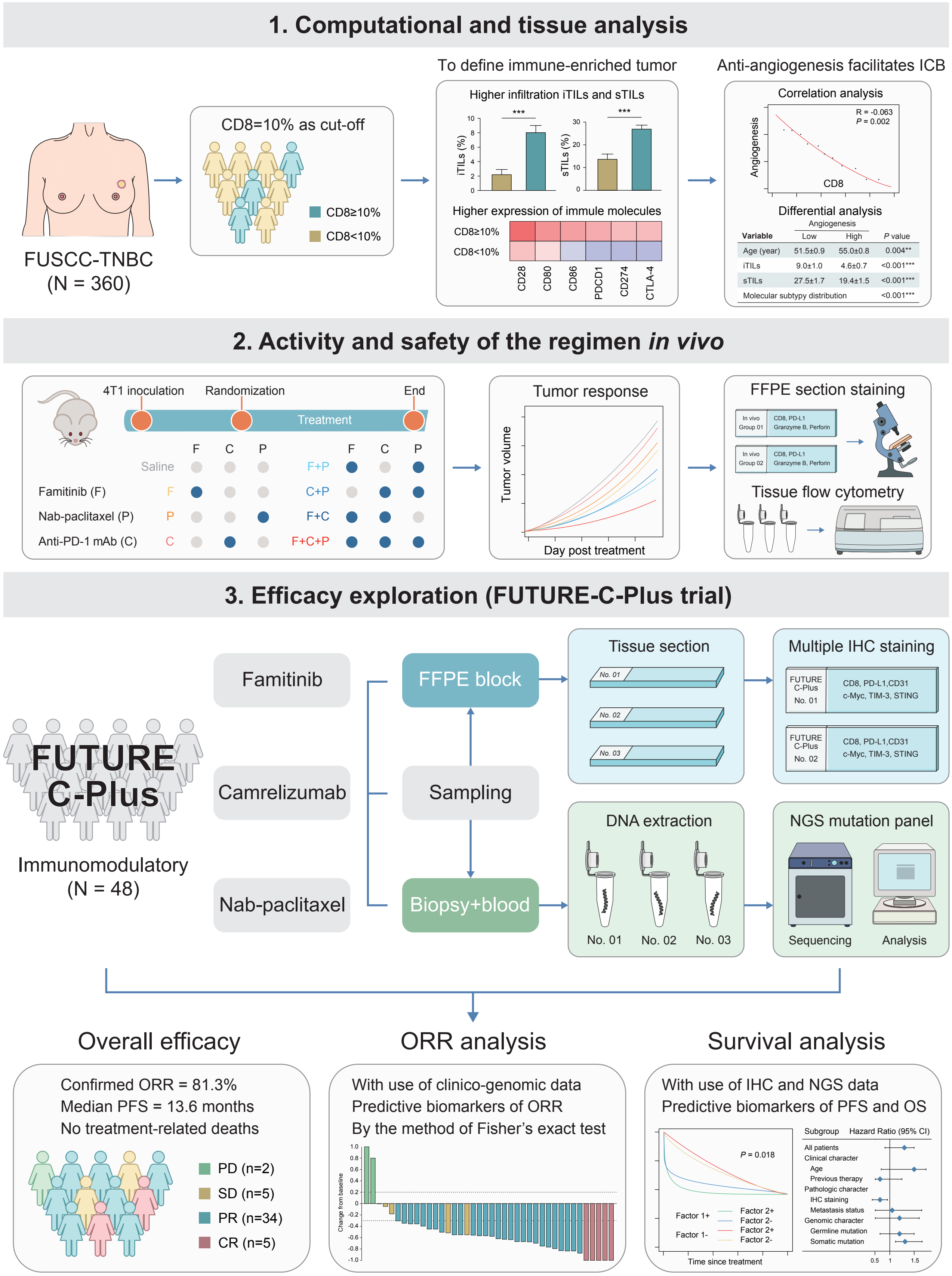

Supplementary Figure 4. Schematic diagram of the study. With the use of a tissue analysis approach, *in vivo* exploration, and patient-level validation, a new patient selection criterion and a novel triplet combination regimen were developed. The biomarkers found in the post hoc analyses await further confirmation in the subsequent randomized controlled FUTURE-SUPER trial. TNBC, triple-negative breast cancer; ICB, immune checkpoint blockade; FFPE, formalin-fixed, paraffin-embedded; IHC, immunohistochemistry; NGS, next-generation sequencing; ORR, objective response rate; PFS, progression-free survival; OS, overall survival.

Supplementary Table 1. Treatment-related adverse events grouped by CD8 status (N=48).

Adverse Event	All	≤20%	>20%	Grade 1-2	≤20%	>20%	Grade 3-4	≤20%	>20%
**Hematological toxicity**									
Neutropenia	38 (79.2)	29 (82.9)	9 (69.2)	22 (45.8)	18 (51.4)	4 (30.8)	16 (33.3)	11 (31.4)	5 (38.5)
Anaemia	10 (20.8)	8 (22.9)	2 (15.4)	5 (10.4)	4 (11.4)	1 (7.7)	5 (10.4)	4 (11.4)	1 (7.7)
Thrombocytopenia	9 (18.8)	4 (11.4)	5 (38.5)	5 (10.4)	1 (2.9)	4 (30.8)	4 (8.3)	3 (8.6)	1 (7.7)
Febrile neutropenia	5 (10.4)	4 (11.4)	1 (7.7)	0 (0.0)	0 (0.0)	0 (0.0)	5 (10.4)	4 (11.4)	1 (7.7)
**Non-hematological toxicity**									
Anorexia	39 (81.3)	28 (80.0)	11 (84.6)	36 (75.0)	25 (71.4)	11 (84.6)	3 (6.3)	3 (8.6)	0 (0.0)
Fatigue	36 (75.0)	26 (74.3)	10 (76.9)	33 (68.8)	23 (65.7)	10 (76.9)	3 (6.3)	3 (8.6)	0 (0.0)
TSH increase	26 (54.2)	19 (54.3)	7 (53.8)	26 (54.2)	19 (54.3)	7 (53.8)	0 (0.0)	0 (0.0)	0 (0.0)
Nausea	23 (47.9)	18 (51.4)	5 (38.5)	23 (47.9)	18 (51.4)	5 (38.5)	0 (0.0)	0 (0.0)	0 (0.0)
Vomiting	12 (25.0)	10 (28.6)	2 (15.4)	12 (25.0)	10 (28.6)	2 (15.4)	0 (0.0)	0 (0.0)	0 (0.0)
Peripheral sensory neuropathy	11 (22.9)	5 (14.3)	6 (46.2)	10 (20.8)	4 (11.4)	6 (46.2)	1 (2.1)	1 (2.9)	0 (0.0)
Hypertension	10 (20.8)	5 (14.3)	5 (38.5)	8 (16.7)	3 (8.6)	5 (38.5)	2 (4.2)	2 (5.7)	0 (0.0)
Hypothyroidism	10 (20.8)	7 (20.0)	3 (23.1)	8 (16.7)	6 (17.1)	2 (15.4)	2 (4.2)	1 (2.9)	1 (7.7)
ALT/AST increase*	8 (16.7)	3 (8.6)	5 (38.5)	7 (14.6)	3 (8.6)	4 (30.8)	1 (2.1)	0 (0.0)	1 (7.7)
Palmar-plantar erythrodysaesthesia	8 (16.7)	5 (14.3)	3 (23.1)	8 (16.7)	5 (14.3)	3 (23.1)	0 (0.0)	0 (0.0)	0 (0.0)
Reactive cutaneous capillary endothelial proliferation	4 (8.3)	3 (8.6)	1 (7.7)	4 (8.3)	3 (8.6)	1 (7.7)	0 (0.0)	0 (0.0)	0 (0.0)
Proteinuria	1 (2.1)	1 (2.9)	0 (0.0)	0 (0.0)	0 (0.0)	0 (0.0)	1 (2.1)	1 (2.9)	0 (0.0)
Septicaemia	1 (2.1)	0 (0.0)	1 (7.7)	0 (0.0)	0 (0.0)	0 (0.0)	1 (2.1)	0 (0.0)	1 (7.7)
Immune related myocarditis	1 (2.1)	1 (2.9)	0 (0.0)	0 (0.0)	0 (0.0)	0 (0.0)	1 (2.1)	1 (2.9)	0 (0.0)

Hepatobiliary disorders (cirrhosis)	1 (2.1)	0 (0.0)	1 (7.7)	0 (0.0)	0 (0.0)	0 (0.0)	1 (2.1)	0 (0.0)	1 (7.7)
**Potential immune-related adverse events**									
TSH increase	26 (54.2)	19 (54.3)	7 (53.8)	26 (54.2)	19 (54.3)	7 (53.8)	0 (0.0)	0 (0.0)	0 (0.0)
Hypothyroidism	10 (20.8)	7 (20.0)	3 (23.1)	8 (16.7)	6 (17.1)	2 (15.4)	2 (4.2)	1 (2.9)	1 (7.7)
Reactive cutaneous capillary endothelial proliferation	4 (8.3)	3 (8.6)	1 (7.7)	4 (8.3)	3 (8.6)	1 (7.7)	0 (0.0)	0 (0.0)	0 (0.0)
ALT/AST increase*	1 (2.1)	0 (0.0)	1 (7.7)	0 (0.0)	0 (0.0)	0 (0.0)	1 (2.1)	0 (0.0)	1 (7.7)
Immune related myocarditis	1 (2.1)	1 (2.9)	0 (0.0)	0 (0.0)	0 (0.0)	0 (0.0)	1 (2.1)	1 (2.9)	0 (0.0)

NOTE. Data are presented No. (%).

Data in CD8 10-20% (n=35) and >20% (n=13) subgroups are shown in two columns, respectively.

*One case of ALT/AST increase was defined as potential immune-related adverse event.

Abbreviation: ALT, alanine aminotransferase; AST, aspartate aminotransferase; TSH, thyroid stimulating hormone.

Combination of famitinib with camrelizumab plus nab-paclitaxel as first-line treatment for advanced, immunomodulatory triple-negative breast cancer (FUTURE-C-PLUS): an open-label, single-arm, phase 2 trial

Sponsor: Fudan University Shanghai Cancer Center

Principal Investigator: Zhi-ming Shao

Version No.: 1.1

Version Date: August 21, 2019

Table of Contents

Table of Contents 1
1. Introduction: background and scientific rationale 17
1.1. Background 17
1.1.1. Overview of breast cancer, chemotherapy status and challenges of triple-negative breast cancer 17
1.1.2. Information on the drugs 18
1.2. Scientific rationale 23
1.2.1. Rationale for the combination therapy 23
1.2.2. Design rationale for triple-agent combination regimen 27
1.3 Potential risks and benefits 31
1.3.1 Known potential risks 32
1.3.2 Known possible benefits 32
2. Study objectives and endpoints 32
2.1 Study objectives 32
2.1.1 Primary objective 32
2.1.2 Secondary objectives 32
2.2 Study endpoints 33
2.2.1 Primary endpoint 33
2.2.2 Secondary endpoints 33
3. Study design 34
3.1 Overall design 34
3.2 Measures to minimize bias 35
4. Subject's screening and withdraw 35
4.1 Inclusion criteria 35
4.2 Exclusion criteria 37
4.3. Lifestyle requirements 39
4.3.1. Contraception 39
4.4. Subject's withdrawal from the study or discontinuation of the treatment 39
4.4.1. Withdrawal from the study 39
4.4.2. Criteria for discontinuation of study treatment 40
4.4.3. Steps to withdraw or discontinue study treatment 40
4.5. Definition of the end of study 41
5. Investigational product management 41
5.1. Investigational product delivery 41
5.2. Route of administration 41
5.3. Drug storage and management 41
6. Study procedures 41
6.1. Screening 41
6.2. Enrollment 44
6.3. Treatment period 44
6.4. Follow-up period 46
6.5. Last visit 47
6.6. Withdrawal from the study 47

6.7. Unscheduled visits 47
6.8. Rescue treatment/alternative treatment 47
7. Assessment 48
7.1. Efficacy assessment 48
7.1.1. Imaging assessment 48
7.1.2. Primary endpoint 50
7.1.3. Secondary endpoint 50
7.2. Safety assessment 51
7.2.1. Pregnancy test 51
7.2.2. Adverse events 51
7.2.3. Laboratory safety assessment 51
7.2.4. Vital signs and physical examination 52
7.2.5. 12-Lead ECG 53
7.3. Pharmacokinetics and immunogenicity assessment 53
7.3.1. Pharmacokinetic and immunogenic blood sample collection 53
8. Dose adjustment 53
8.1. Overall regulations of dosage 53
8.1.1. Administration interruption and dose adjustment of camrelizumab 54
8.1.2. Administration interruption and dose adjustment of nab-paclitaxel 57
8.1.3. Administration adjustment of famitinib 58
9. Concomitant treatment 59
10. Treatment of common adverse reactions 59
10.1. Treatment of hand-foot syndrome 59
10.2. Treatment of hepatic toxicity 60
10.3. Treatment of hypertension 61
10.4. Treatment of proteinuria 61
10.5. Guidelines for supportive treatment of immuno-oncology drug Camrelizumab 61
10.6. Infusion Reaction Management 63
11. Adverse event report 64
11.1. Adverse events (AE) 64
11.1.1. Definition of AE 64
11.1.2. Criteria for the severity of AEs 65
11.1.3. Criteria for the relationship between AE and study drug 65
11.2. Serious adverse events (SAE) 66
11.2.1. Definition of SAE 66
11.2.2. Hospitalization 67
11.2.3. Disease progression and death 67
11.2.4. Other anti-tumor treatment 68
11.2.5. SAE reporting system 68
11.3. Pregnancy 68
11.4 Follow-up of AE/SAE 69
12. Data analysis/statistical methods 70
12.1 Sample size 70
12.2 Statistical analysis plan 70

12.3 Statistical assumptions and discriminant rules 70
12.4 Analyzed population 70
12.5. Statistical methods 70
12.5.1 Basic methods 70
12.5.2 Primary efficacy endpoint analysis 70
12.5.3 Secondary efficacy endpoint analysis 71
12.5.4 Processing of missing data 71
12.5.5 Safety analysis 71
12.5.6 Pharmacokinetic analysis 71
12.5.7 Immunogenicity analysis 72
13. Data processing 72
13.1 Requirements for data filled by investigators 72
13.2 Traceability of data, filling of Case Report Form (CRF) 72
14. Quality assurance and quality control 72
15. Ethics 72
16. Study progress, data storage 73
16.1 Study progress 73
16.2 Data storage 73
17. Reference 73
Appendix 1: Physical Condition Scoring Criteria (ECOG, Eastern Cooperative Oncology Group) 74
Appendix 2: Clinical Staging Criteria for Breast Cancer (AJCC breast cancer TNM staging) 75
Appendix 3: Response Evaluation Criteria in Solid Tumors 76
Appendix 4: Classification criteria for AE of drug (NCI-CTCAE Version 5.0) 88

Synopsis of Clinical Trial Protocol

**Title**	Combination of Famitinib with Camrelizumab plus Nab-paclitaxel as first-line treatment for patients with immunomodulatory advanced triple-negative breast cancer (FUTURE-C-PLUS): a prospective, single-arm, phase 2 study
**Version No.**	1.1
**Study objectives**	**Primary objective**<br>To evaluate the objective response rate (ORR) of PD-1 antibody camrelizumab plus nab-paclitaxel and famitinib as a first-line therapy in patients with unresectable local advanced or metastatic triple-negative immunomodulatory breast cancer.<br>**Secondary objectives**<br>To evaluate the progression-free survival (PFS), overall survival (OS), Duration of Response (DoR) and Disease Control Rate (DCR) of PD-1 antibody camrelizumab plus nab-paclitaxel and famitinib as a first-line therapy in patients with unresectable local advanced or metastatic triple-negative immunomodulatory breast cancer;<br>To evaluate the safety of PD-1 antibody camrelizumab plus nab-paclitaxel and famitinib as a first-line therapy in patients with unresectable local advanced or metastatic triple-negative immunomodulatory breast cancer;<br>To evaluate the pharmacokinetics (PK) characteristics of PD-1 antibody camrelizumab plus nab-paclitaxel and famitinib as a first-line therapy in patients with unresectable local advanced or metastatic triple-negative immunomodulatory breast cancer;<br>To evaluate the immunogenicity of PD-1 antibody camrelizumab plus nab-paclitaxel and famitinib as a first-line therapy in patients with unresectable local advanced or metastatic triple-negative immunomodulatory breast cancer.<br>**Exploratory objective**<br>To assess predictive and prognostic exploratory biomarkers in archival and/or fresh tumor tissue and blood and their association with disease status and/or response to study treatment.

**Study endpoints**	Primary endpoint<br>Objective response rate (ORR)<br>Secondary endpoints<br>PFS<br>OS<br>DCR<br>DoR<br>Safety endpoints: the incidence and grade of adverse events (AE) and severe adverse events (SAE); AE is assessed according to the NCI-CTC AE 5.0; changes of the following indicators compared to their corresponding baselines: ECOG performance status, vital signs, physical examination, laboratory examination indicators (hematology, urinalysis, routine stool test, blood chemistry test), 12-lead electrocardiogram (ECG), echocardiogram, etc.<br>PK parameters of PD-1 antibody camrelizumab plus nab-paclitaxel and famitinib<br>The proportion of subjects who produce anti-SHR-1210 antibody and the level of the antibody<br>The predictive and prognostic exploratory biomarkers in archival and/or fresh tumor tissue and blood and their association with disease status and/or response to study treatment.
**Study population**	The subjects enrolled in this study are patients diagnosed with unresectable local advanced or metastatic triple-negative immunomodulatory breast cancer by the Department of Pathology, Fudan University Shanghai Cancer Center. Triple-negative refers to ER negative (IHC ER positive <1%) and PR negative (IHC PR positive <1%) and HER2 negative (IHC-/+; or IHC++, but FISH/CISH-). The definition of immunomodulatory type refers to the proportion of CD8+ cells is ≥10% in immunohistochemistry test.<br>Patients with unresectable local advanced or recurrent/metastatic triple-negative immunomodulatory breast cancer have not received any systemic anti-tumor treatment during the recurrence and metastasis stage.<br>Before enrollment, all subjects must provide the diagnostic unstained pathological sections. If the metastatic lesion is a single measurable target lesion, the pathological evidence must be based on the biopsy of the metastatic lesions. If the biopsy of the metastatic lesions cannot be obtained, then the biopsy of the primary lesion shall be used as the pathological basis, but the diagnostic result must be confirmed as triple-negative immunomodulatory type through pathological consultation by Fudan University Shanghai Cancer Center.
**Study design**	This is an open-label, single-arm, phase 2 clinical trial.<br>Forty-six patients with unresectable local advanced or metastatic triple-negative immunomodulatory breast cancer are intended to be enrolled in this trial, to guarantee the total number of evaluable patients is not < 41 (estimated by drop-out rate of 10%). The eligible patients have never received taxanes treatment, or

	received taxanes treatment as adjuvant therapy/neoadjuvant therapy but the time to relapse is greater than 6 months, while not having received any systemic anti-tumor treatment in the advanced stage. After enrollment, the patients are administered with PD-1 antibody camrelizumab (200 mg intravenously, once every 2 weeks, with a treatment cycle of 4 weeks), paclitaxel (albumin bound,100 mg/m^2 intravenously, once weekly, every consecutive 3 weeks followed by a week break, with a treatment cycle of 4 weeks), initial dose of famitinib malate (20 mg, once daily, dose can be reduced to 15 mg in case of intolerance, with a treatment cycle of consecutive 4 weeks). The target treatment cycle of nab-paclitaxel is 6 weeks, without an upper limit. The triple-agent combination therapy should continue until disease progression, intolerable toxicity, withdrawal of informed consent or treatment discontinuation at investigator's discretion (whichever occurs first). When the treatment is finished, a 28-day safety follow-up should be conducted since subject's last administration until all adverse events resolve to ≤ grade I, or all adverse events are clinically stable (whichever occurs later); subjects that are discontinued the treatment due to non-PD, non-death reasons should be followed up for efficacy until PD, starting to receive other anti-tumor treatment or death(whichever occurs first); all subjects should be followed up for survival (OS data collection) until death, loss to follow-up or trial termination (whichever occurs first).<br>The overall design of this study is as follows:

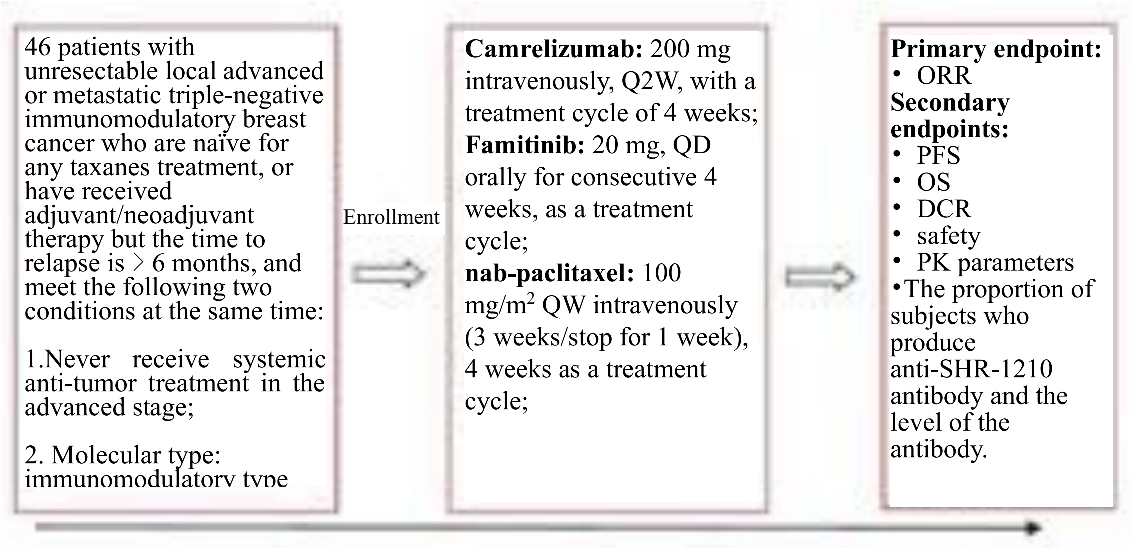

The assessment will be conducted every 2 cycles (every 8 weeks) until disease progression or discontinuation from the trial.

**Administration route**	Anti-PD-1 antibody camrelizumab: 200 mg intravenously Q2W, with a treatment cycle of 4 weeks;<br>Famitinib malate: the starting dose is at 20 mg QD, orally, for consecutive 4 weeks as a treatment cycle, dose can be reduced to 15 mg in case of tolerance;<br>nab-paclitaxel: 100 mg/m^2 QW intravenously, once weekly, every consecutive 3 weeks followed by a week break, with a treatment cycle of 4 weeks; at least 6 cycles should be completed, without upper limit.<br>All study drugs will be administered continuously per administration cycle until disease progression, intolerable toxicity, withdrawal of the informed consent or discontinuation at investigator's discretion.
**Inclusion criteria**	Patients can be enrolled in this trial only if they meet all the following inclusion criteria:<br>1. Age ≥18 years old and ≤70 years old.<br>2. ECOG status of 0 to 1.<br>3. The expected survival period is not < 3 months.<br>4.Advanced triple-negative invasive breast cancer is confirmed by pathological examination, as well as meets the following conditions:<br>The definition of triple-negative breast cancer (TNBC) refers to the breast cancer with estrogen receptor (ER), progesterone receptor (PR) and human epidermal growth factor receptor 2 (HER-2) all negative in pathological examination. Specifically: ER negative: IHC<1%, PR negative: IHC<1%, HER2 negative: IHC-/+ or IHC++ but FISH/CISH negative. All specimens need to be verified by the Pathology Department of Fudan University Shanghai Cancer Center , and patients with recurrence and metastasis must have their molecular classification re-examined;<br>Tumor staging: recurrent or metastatic breast cancer; patients with local recurrence must be confirmed by the investigator that they cannot receive radical surgical resection;<br>Definition of immunomodulatory type: The proportion of CD8+ cells is ≥10% in IHC staining analysis.<br>5. Patients who have never received taxanes treatment or received taxanes treatment as adjuvant therapy/neoadjuvant therapy but the interval between the end of treatment and relapse is greater than 6 months, or have not received systemic anti-tumor treatment; (chemotherapy, targeted therapy etc.) in the advanced stage of cancer.<br>6. At least one extracranial target lesion with measurable diameter can be identified based on imaging judgment. (RECIST 1.1 standard).<br>7. The major organs are functioning well, namely the relevant examinations meet the following requirements within 7 days before the first administration:<br>1) Routine blood test:

	ANC≥1.5×10^9/L<br>PLT≥90×10^9/L<br>Hb≥90 g/L<br>2) Blood chemistry test:<br>TBIL≤1.5×ULN<br>Serum cholesterol ≤ 1.25 x ULN; triglyceride ≤ 2.0 x ULN<br>ALT and AST≤2.5×ULN; for patients with hepatic metastases, ALT and AST≤5×ULN<br>BUN and Cr≤1.5×ULN and creatinine clearance rate ≥50 mL/min (Cockcroft-Gault formula)<br>PT and APTT ≤ 1.5 x ULN<br>3) Echocardiography:<br>LVEF ≥50%<br>4) 12-lead ECG:<br>QT interval (QTcF) corrected by Fridericia method <450 ms for males and < 470ms for females;<br>8. Patients join this study voluntarily, sign the informed consent form, with good compliance and are also cooperative during follow-up.
**Exclusion criteria**	Patients will not be included in this study if they have any of the following conditions:<br>1. Patients who have received taxanes treatment as adjuvant/neoadjuvant therapy, but the interval between the end of paclitaxel treatment and recurrence and metastasis were <6 months.<br>2. Patients who have previously received treatment (except bevacizumab) with VEGFR class of small molecule tyrosine kinase inhibitors (such as famitinib, sorafenib, sunitinib, regorafenib, etc.).<br>3. Patients with a medical history of hemorrhage, and any severe bleeding event that reached grade 3 or above in CTCAE5.0 within 4 weeks before screening.<br>4. Imaging shows that the tumor has invaded the periphery of important blood vessels or it is highly possible that the tumor will invade important blood vessels and cause fatal hemorrhage during treatment at investigator's discretion.<br>5. Patients with abnormal coagulation function and bleeding tendency (14 days before randomization, the patient must meet the standard: INR is within the normal range without anticoagulant); patients that are treated with anticoagulant or vitamin K antagonist such as warfarin, heparin or its analogues; under the premise that the prothrombin time International Normalized Ratio (INR, International Normalized Ratio) ≤1.5, the use of low-dose warfarin (1 mg orally, once daily) or low-dose aspirin (not exceed 100 mg/day) is permitted for the purpose of prevention.<br>6. Patients with arteriovenous thrombosis occurred within one year before screening, such as cerebrovascular accidents (including transient ischemic attacks), deep vein thrombosis (except those who recovered, as confirmed by

	the investigator, from venous thrombosis caused by intravenous catheterization due to pre-chemotherapy) and pulmonary embolism, etc.;<br>7. Patients with a history of autoimmune diseases or those using glucocorticoids or immunosuppressive drugs;<br>8. Patients with swallowing inability, chronic diarrhea and intestinal obstruction, or with other conditions that affect the administration and absorption of medications;<br>9. Patients with a third space effusion that cannot be controlled by drainage or other methods (such as large amounts of pleural fluid and ascites);<br>10. Patients that have received radiotherapy, chemotherapy, surgery or other targeted and immunotherapy for advanced triple-negative breast cancer within 4 weeks before receiving the first study treatment;<br>11. Patients that have not recovered from the AEs of the previous medication before receiving the first treatment at investigator's discretion (NCI-CTCAE version 5.0 classification> grade 1);<br>12. Patients with untreated central nervous system (CNS) disease (patients with treated, asymptomatic treated CNS metastases also eligible.);<br>13. Patients that participated in other anti-tumor drug clinical trials within 4 weeks before receiving the first administration;<br>14. Patients with long-term unhealed wounds or fractures with incomplete healing;<br>15. Patients whose urinalysis showed that urine protein ≥ 2+ and 24h urine protein quantitative> 1 g was confirmed;<br>16. Patients with past or current history of pulmonary fibrosis, interstitial pneumonia, pneumoconiosis, radiation pneumonia, drug-related pneumonia, or severely impaired lung function;<br>17. Patients with known active stage of HBV or HCV infection or hepatitis B DNA ≥500, or patients with chronic abnormal liver function;<br>18. Patients who suffered from other malignant tumors in the past 5 years, excluding cured cervical carcinoma in situ, skin basal cell carcinoma or skin squamous cell carcinoma;<br>19. Patients with allergies, or those who are known of having a medical history of allergies to any drug components of this study; or those who are allergic to other monoclonal antibodies;<br>20. Patients with active infections or those who have been treated with systemic immune stimulating factors within 4 weeks before enrollment;<br>21. Patients with a history of immunodeficiency, including HIV positive, or have other acquired or congenital immunodeficiency diseases, or have a history of organ transplantation; or have a positive syphilis antibody test;<br>22. For female subjects: patients that are non-surgical sterilized or non-menopausal refused to use a medically approved contraceptive method during the study treatment period and within 6 months after the end of the study treatment; women of childbearing age are positive in serum or urine pregnancy test within 7 days before enrolled in this study, or in lactating

	period;<br>23. Patients with hypertension that cannot be controlled by a single antihypertensive drug (systolic blood pressure> 140 mmHg, diastolic blood pressure> 90 mmHg); those with a history of unstable angina; those who are newly diagnosed with angina within 3 months before screening or with a myocardial infarction occurrence within 6 months before screening; patients with arrhythmia (including QTcF: male ≥450 ms, female ≥470 ms) that requires long-term use of antiarrhythmic drugs or with cardiac insufficiency that ≥ grade 2 in New York Heart Association grading;<br>24. Patients with a history of abnormal thyroid function;<br>25. Patients with Grade ≥ 2 peripheral neuropathy;<br>26. Patients with a clear history of neurological or mental disorders, including epilepsy or dementia;<br>27. Patient with any other condition that is considered not suitable to participate in this study at investigator's discretion.
**Withdrawal from the study**	The subject can voluntarily withdraw from the trial at any time, or may be required to withdraw from the trial due to safety or behavioral reasons, or unable to comply with the study visit schedule or procedures required by the protocol of the clinical research center where he or she belongs to.<br>Reasons for the subject's withdrawal from the study may include:<br>The subject withdraws the informed consent to participate in the study and refuses further follow-up;<br>Clinical adverse events, abnormal values of laboratory tests or complicated diseases, and the investigator believes that continuing to participate in the trial is not in the best interests for the subjects;<br>Other situations where the investigator believes that it is necessary for the patients to withdraw from the study, such as the subject's losing the ability to express their will freely due to imprisonment or isolation;<br>Lost to follow-up;<br>The subject's death;<br>The investigator terminates the study<br>The reason for the subject's withdrawal must be recorded in the Case Report Form and the subject's medical records.<br>It should be noted that the withdrawal of informed consent means that the subject has withdrawn the consent for further contact, or no longer agrees to the previously authorized person to provide further information. Whenever possible, the subject should notify the investigator in written notice that he has decided not to receive follow-up. The investigator should make every effort to explain and record the withdrawal of informed consent, and clarify the withdrawal is whether the patient is not willing to take the study drug, or not willing to be followed up by the visit specified by the trial protocol at the same time.
**Determination of sample size**	Assuming that the point-estimate value of ORR of the treatment group is 0.6, 41 subjects are needed if the width of the two-sided 95% confidence interval

	is 0.3, Considering the drop-out rate of 10%, therefore, a total of 46 subjects are needed.
**Data analysis and statistical methods**	Categorical data will be descriptively summarized using statistics including frequency and percentage, and 95% confidence intervals for the overall percentage of cases will be presented when necessary. Continuous data will be descriptively summarized using statistics including mean, standard deviation, median, minimum and maximum. Time-event data will be used to estimate the survival function, draw the survival curve and estimate the median time and its 95% confidence interval by employing the Kaplan-Meier method.<br>Safety analysis: descriptive statistics will be conducted using the following data (but not limited to) based on the safety set. Summary of adverse events; incidence and severity of adverse events; summary of serious adverse events; correlation analysis of adverse events; laboratory examinations, vital signs, electrocardiogram data and the changes from the baseline, etc.<br>Efficacy analysis: the objective response rate (ORR), disease control rate (DCR) and their corresponding 95% confidence interval are estimated (Clopper-Pearson method). The PFS, DoR, OS and their corresponding 95% confidence interval are estimated (Kaplan-Meier method) and calculated (Brookmeyer-Crowley method based on the log-log transformation, the standard error is calculated using the Greenwood formula).<br>Pharmacokinetic analysis: A population pharmacokinetic model will be used to explore the pharmacokinetic features of SHR-1210, famitinib and paclitaxel.

Schedule of Activities

Items	Screening period	Treatment period					End of treatment/withdrawal from the study	After treatment		Survival follow-up
		First cycle		Start of second cycle				Safety follow-up	Efficacy follow-up	
	**D-28-D-1**	**D8±1**	**D15±1**	**D1±1**	**D8±1**	**D15±1**		28 days after last dosing of test drug (±7)	Non-PD non-death patients	Every 12 weeks (±7 days)
Informed consent	√									
Demography	√									
Medical history	√									
Histopathological examination	√									
ECOG performance status	√ (Within 7 days)			√			√	√		
Physical examination	√ (Within 7 days)			√			√	√		
Vital sign	√ (Within 7 days)			√			√	√		
Routine blood test	√ (Within 7 days)	√	√	√	√	√	√			
Routine urine test	√ (Within 7 days)			√			√			
Fecal occult blood	√ (Within 7 days)			√			√			
Blood chemistry test	√ (Within 7 days)	√	√	√	√	√	√			
Coagulation function test	√ (Within 7 days)			√			√			

Thyroid function	√ (Within 7 days)			√			√			
Tumor markers (CEA、CA153、CA125)	√ (Within 7 days)			√			√			
Pregnancy test	√ (Within 7 days)	When necessary					√			
Infectious disease screening	√ (Within 7 days)									
Electrocardiogram	√ (Within 7 days)			√			√			
Echocardiogram	√ (Within 7 days)			√			√			
Imaging examination	√	Every 8 weeks (±7 days)								
Bone scan	√	When necessary								
Judgment of inclusion and exclusion	√									
PK blood sample collection [18]			√	√				√		
Camrelizumab		Dosing on D1±1, D15±1 per cycle								
nab-paclitaxel		Dosing on D1±1, D8±1, D15±1 per cycle								
Famitinib		Daily dosing								
Subject log		√					√			

Concomitant medication /treatment	√			√			√	√		
Return study drug		Paclitaxel: return on D1±1, D8±1, D15±1/cycle;<br>Camrelizumab: return on D1±1 and D15±1;<br>famitinib: return on D1±1 (the last cycle)					√			
Record of AE	√			√			√	√		
Time at progression/death									√	√
Further treatments for the tumor								√	√	√

Note:

1) Examinations and tests are carried out according to the schedule of activities, regardless of the duration of drug discontinuation. The following examinations shall be completed within the time window listed in the test procedure. The examinations can be carried out in advance if there are statutory holidays, and reasons for time window exceedance should be recorded in CRF;

2) Wax blocks or pathological sections of tumor tissues are collected during the screening period for genetic testing or other exploratory studies, and tumor biopsy is performed if necessary.

1. Informed consent form: it must be signed before all test operations but not limited to within 28 days before the enrollment.

2. Demographic data (initials, gender, ethnicity, marital status, date of birth, height, weight, and body surface area and body mass index are calculated based on height and weight);

3. Medical history inquiry: including past medical history and treatment history (clinical/pathological diagnosis, diagnosis time, clinical/pathological staging, HER2/ER/PR/expression; whether he has accepted surgery, neoadjuvant treatment, adjuvant treatment, and radiotherapy or not, when to progression and the rationale for diagnosis of the disease progression; treatment for recurrent/metastatic breast cancer, such as surgery, chemotherapy, targeted therapy, etc.), history of smoking and drinking, history of drug allergy (drug name, allergy symptoms), concomitant disease and medication (name of disease, and name, dosage, and usage of concomitant medication);

4. Vital signs: including body temperature, blood pressure, respiratory rate and pulse (blood pressure monitoring: blood pressure is monitored 3 times a day for 2 weeks before treatment with famitinib. Abnormal blood pressure should be monitored daily; normal blood pressure should be monitored twice weekly).

5. Physical examinations: including general conditions, skin mucous membrane, lymph nodes, head and neck, chest, abdomen, musculoskeletal, neural reflex, respiratory system, cardiovascular system, genitourinary system (if necessary), mental status, etc.; height is generally measured only at baseline, and weight must be measured for each physical examination.

6. Hematology: including absolute counts of WBC, ANC, LC, RBC, Hb, PLT; hematology should be performed before weekly chemotherapy with paclitaxel.

7. Urinalysis: including urine protein, urine sugar, and urine occult blood. If the urinalysis shows urine protein++ or above, please perform the 24h urine protein quantification;

8. Routine stool test: including fecal occult blood;

9. Blood biochemistry tests: including Glucose, TP, A/G, ALT, AST, ALP, γ-GT, ALB, TBIL, DBIL, IBIL, TG, CHOL, UA, BUN, Cr, K+, Na+, Mg2+, CL-, Ca2+, P; myocardial enzyme spectrum test can be performed if necessary, which is determined by the investigator according to the conditions of the subjects;

10. Coagulation function: PT, APTT, TT, Fbg and D-dimer;

11. Thyroid function: free T3, free T4, T3, T4 and TSH;

12. Infectious disease screening: including five indicators of hepatitis B virus, HIV and HCV antibody test; if hepatitis B surface antigen is positive, virological examination is required.

13. Pregnancy test: A blood HCG test should be performed among female subjects of childbearing age during the screening period to rule out pregnancy; a urine pregnancy test can be performed in subsequent examinations, and if the result is positive, a blood HCG test should be performed to confirm pregnancy.

14. 12-lead electrocardiogram: Pay attention to the heart rate, QT, QTc and P-R time; the test shall be repeated if necessary (at least 10 minutes apart).

15. Echocardiography: The Echocardiography can be performed within 28 days before randomization (including qualified UCG completed before signing the ICF). If symptoms such as chest pain and palpitations occur during the study, additional examinations can be carried out as appropriate;

16. Tumor imaging examination: At least multi-slice spiral CT or enhanced MR scan of the head, chest and abdomen are performed during the screening period. At least multi-slice spiral CT or enhanced MR scan of the chest and abdomen are performed during the follow-up. The investigator will decide whether the patient requires head imaging re-examination based on the patient's clinical symptoms. The scan of sites including neck and pelvic can be further performed in tumor assessment at baseline or later depending on clinical situations. The time window for tumor imaging assessment is ±7 days. The report within 28 days before the first dose is required during the screening period (including qualified tumor imaging examinations completed before signing the ICF). The time point of tumor imaging examination during the administration period is determined from the beginning of the treatment, regardless of the time during which the administration is suspended due to toxicity. All imaging assessments will continue until the disease progression is confirmed by the investigator. Every effort must be made to persuade subjects who are withdrawn due to intolerance of toxicity to continue imaging assessment until the disease progression, new anti-tumor therapy is adopted, or they are lost to follow-up.

17. Bone scan: A bone scan should be performed before randomization among patients who have not had a bone scan within 28 days before the first medication. Positive bone lesions need to be re-examined by bone CT/MRI (or X-ray), and bone metastases are followed up in the same way as the baseline according to the time point of tumor imaging examination. During the treatment and follow-up period, the investigator will decide whether the patient requires another bone scan or bone CT/MRI based on the patient's clinical symptoms.

18. PK blood sample collection: SHR-1210: Collect blood samples, separate serum, and detect SHR-1210 blood concentration and immunogenicity within 30 minutes before the administration of each cycle from the first to the fourth cycle, within 30 minutes before the administration for every subsequent 4 cycles (12 weeks), and 28 days after the end of treatment (±7 days). Famitinib and nab-paclitaxel: blood samples are collected and the plasma is separated within 30 minutes before all dosing in the third week of the first cycle, within 5 minutes and 4 hours (±10 minutes) after the end of dosing, and the concentration of famitinib and paclitaxel are detected respectively. The time of administration on the day before and on the day of famitinib administration are recorded in detail.

19. Previous/concomitant medication: Concomitant medications within 28 days before the first medication and during the trial; only concomitant medications and treatments for new or unresolved AEs related to the treatment should be recorded once the treatment of a subject is discontinued. The concomitant medications and treatments for AEs (whether related to the study drug or not) still present at the visit during treatment discontinuation should be followed up until AE is relieved, disappears or 12 months after the last administration.

20. Observation records of adverse events: AEs are monitored from the day of signing the informed consent form, until 28 days after the last administration [AEs (whether related to the study drug or not) that still exist at the visit during discontinuation of the treatment shall be followed up until the AE is relieved, disappears or 12 months after the last medication]; adverse events, concomitant medication/treatment and unplanned inspections should be recorded in detail during the study.

21. Further treatments for tumors: During the period from the discontinuation of study drug to the end of survival follow-up, it should be recorded whether the subjects have adopted other tumor therapy; during survival follow-up, only the results of tumor therapy should be recorded, and no concomitant medication for other diseases should be recorded.

22. Efficacy follow-up: Subjects who discontinue medication for non-disease progression and non-death causes should receive efficacy follow-up after the end of the study drug treatment, as well as tumor imaging assessment at the time point specified in the protocol until disease progression, use of other anti-tumor drugs or death (whichever occurs first); each time point of follow-up, tumor imaging assessment results and other anti-tumor treatment information should be recorded in detail.

23. Survival follow-up (OS data collection): Subjects should receive survival follow-up after the treatment discontinuation visit. Since the completion of the treatment discontinuation visit, subjects themselves, their family members or local physicians will be followed up in the clinic or by telephone at least every 12 weeks from the date of completion of the treatment discontinuation visit to collect survival (date of death and cause of death) and post-treatment data (including treatment received) until death, loss to follow-up of the subject, or end of OS data collection (whichever occurs first). Each survival follow-up should be recorded in detail in the corresponding section of CRF.

1. Introduction: background and scientific rationale

1.1. Background

1.1.1. Overview of breast cancer, chemotherapy status and challenges of triple-negative breast cancer

Breast cancer ranks first position in the incidence of female cancers in China which is on the rise. It is a common malignant tumor that endangers women's health. In China, according to the data collected by the National Cancer Registry (NCR) in 2017, there were about 279,000 new female breast cancer cases in 2014, with an incidence rate of 41.82/100,000, ranking first position among morbidity rate of female malignant tumors; death cases caused by breast cancer were about 66,000, the mortality rate is 9.9/100,000, and the age of onset is younger than that in western countries. Breast cancer is the generic term of a group of diseases, at least can be categorized into four molecular subtypes: luminal A, luminal B, HER2 over-expression and triple-negative type. Triple-negative breast cancer (TNBC) is a molecular type that lacks estrogen receptor, progesterone receptor and human epidermal growth factor receptor 2, which accounts for about 15% to 20% of the total breast cancer cases. The characteristics of TNBC include high recurrence rate at the early age, high distant metastasis rate, and poor prognosis.

Since there is a lack of effective targeted therapy drugs for recurrent and metastatic TNBC, chemotherapy is still the key method for the treatment of this cancer. As for recurrent and metastatic TNBC, according to the *2018 NCCN Breast Cancer Guidelines* [1] and the *2017 Chinese Anti-Cancer Association(CACA) Breast Cancer Diagnosis and Treatment Guidelines* and norms [2], the routine recommended mono-chemotherapy includes: anthracyclines, such as doxorubicin, epirubicin, pirarubicin and pegylated liposomal doxorubicin; taxanes, such as paclitaxel, docetaxel, and nab-paclitaxel; antimetabolites, such as capecitabine and gemcitabine; non-taxanes type microtubule formation inhibitors, such as vinorelbine and eribulin.

For patients who need to quickly reduce tumor burden and relieve symptoms, combined chemotherapy can also be a treatment option, including: doxorubicin/cyclophosphamide (AC), epirubicin/cyclophosphamide (EC), cyclophosphamide, methotrexate /fluorouracil (CMF), gemcitabine/paclitaxel (GT), etc. Compared with single-agent sequential therapy, combined chemotherapy (ORR/PFS) has better efficacy but greater toxicity. Patients who receive combined chemotherapy still have limited survival period and the OS of the patients cannot benefit from the therapy obviously. A number of clinical studies have shown that patients with recurrent and metastatic TNBC have poorer first-line treatment efficacy with either mono- or combination chemotherapy. The median overall survival period is about 12 months, and the 5-year survival rate is <30%. Currently in our country, chemotherapy is still the recommended treatment for recurrent and metastatic TNBC but finding a more effective treatment for metastatic TNBC has become a hot spot and difficult issue.

More than 50 years ago, scientists discovered that the extent of TILs infiltration was negatively correlated with breast cancer recurrence. With in-depth studies, it is found that the extent of TNBC chemotherapy benefit is also closely related to the density of TILs. Fudan

University Shanghai Cancer Center used high-throughput gene chip and sequencing technology on 465 specimens to draw the largest multi-omics map of TNBC cohort in the world. The study was published on the *Cancer Cell* [3] in March 2019. According to the different characteristics of the subtype surface protein, the study named 4 subtypes in the triple-negative "family": immune-modulated (IM) type, Luminal androgen receptor type (LAR), basal-like immunosuppressant (BLIS), mesenchymal type (MES). It is the first time that the classification standard for TNBC is proposed on the rationale of the big data system, which points out a new direction for finding targets. Among them, the IM subtype accounts for 24% of TNBC, and its prognosis is relatively good. But in this subtype 6% patients still have a recurrence and/or metastasis within 5 years after surgery. This subtype is characterized by increased signal transduction of immune cells in gene expression data, and H&E staining confirms the increase of stromal cells and tumor infiltrating lymphocytes (TILs). Although the mutation load of IM subtypes is not significantly higher than those of other subtypes, gene set enrichment analysis (GSEA) shows that compared with other subtypes, the adaptive immune system and gamma interferon signaling pathway are activated in IM subtypes. This type highly expresses immune-related markers, such as programmed death receptor-1 (PD-1) and its ligand (PD-L1). PD-1 is an important immunosuppressive transmembrane protein on the surface of T cells. PD-1 mainly restrains T cell activity in chronic inflammation, infection or cancer. PD-1 has 2 ligands, PD-L1 and PD-L2. In the tumor microenvironment, tumor cells can express PD-L1 or PD-L2. One way for cancer cells to evade destruction by the immune system is to connect to the PD-1 protein of T cells through ligands. When the ligand is connected to PD-1, T cells cannot detect the tumor and send signals to the immune system to attack the tumor. In addition, the combination of CIBERSORT (single cell type analysis method) and differential expression profile analysis showed that the IM subtypes are simultaneously enriched in immune-activated cells and immune-stimulating factors. Combined clinical and genomics performance characteristics have confirmed that immune recognition is activated in IM subtypes. Therefore, tumors may avoid immune successfully by recruiting immunosuppressive cells or activating immune checkpoint molecules. Although the number of immunosuppressive cells did not increase in the IM subtype, expression profile analysis showed that a variety of immunosuppressive molecules were significantly overexpressed in this subtype, this provides additional theoretical rationale for the use of immune checkpoint blocking therapy.

1.1.2. Information on the drugs

1.1.2.1. Information on nab-paclitaxel

Paclitaxel of injection (albumin-bound type) (hereinafter referred to as "nab-paclitaxel") is a new type of lyophilized nanoparticle paclitaxel with human serum albumin as a carrier. It uses nanotechnology to combine paclitaxel with human serum albumin to produce nanoparticles. Cremo-phor EL, the cosolvent of traditional paclitaxel injection, was not used in nab-paclitaxel, this reduces the potential risk of solvent-related allergic reactions, so pretreatment to prevent allergic reactions is no longer needed before medication, and the drug infusion time can be shortened to 30 minutes. In addition, since albumin has the characteristics of binding and releasing, when human albumin is used as a drug carrier, plus the utilization of albumin receptor Gp60 on the cell membrane and the caveolae, and the

function of cysteine-rich acid secreted protein (SPARC) in tumor tissues, the entry of nab-paclitaxel into tumor cells can be promoted, the purpose of targeted therapy can be achieved, and the anti-tumor efficacy can be enhanced.

Compared with paclitaxel, nab-paclitaxel has better pharmacokinetic properties and faster and higher tissue distribution rate. The distribution rate of nab-paclitaxel is 40 times faster than its discharge rate. When nab-paclitaxel binds to the albumin receptors in the tumor stroma, the concentration of it in the tumor tissue is 26% higher than traditional paclitaxel. In 2005, based on a phase 3 comparative study between nab-paclitaxel and paclitaxel, advanced breast cancer was approved as an indication for nab-paclitaxel (Abraxane). A total of 460 patients with advanced breast cancer were involved in the study. The key efficacy endpoint was response rate: 33% in the nab-paclitaxel group vs 19% in the paclitaxel group (p=0.001); the time to progression between the 2 groups was 23.0 weeks vs 16.9 weeks. In terms of safety: the incidence of grade 4 neutropenia in the nab-paclitaxel group was significantly lower than that in the paclitaxel group: 9% vs 22%. The incidence of grade 3 sensory nerve abnormalities was higher in the nab-paclitaxel group (10% vs 2%), but it was controllable and self-limiting with dose suspension or dose reduction. In addition, due to the improved dosage form of nab-paclitaxel, allergic reactions of nab-paclitaxel are significantly less than those of paclitaxel, and the infusion time is significantly shortened in the clinical application process. The study included a total of 186 cases of "first-line" treatment patients (41% of the total subjects) who had not used paclitaxel for advanced cancer treatment or had used paclitaxel in the previous adjuvant treatment phase but the time to relapse was longer than 1 year. Among first-line patients, the objective response rate of the albumin-paclitaxel group was significantly higher than that of the paclitaxel group (42% vs 27%, p=0.029). The time of progression free survival (PFS) were also extended, although there was no obviously statistical difference (24 weeks vs 19.7 weeks, p=0.173).

In January 2005, the US Food and Drug Administration (FDA) approved the marketing authorization of paclitaxel (albumin bound, abraxane). In September 2018, the National Medical Products Administration (NMPA) of China approved the marketing authorization of paclitaxel (albumin bound, Aiyue, Hengrui Pharmaceuticals), which is suitable for the treatment of metastatic breast cancer with failure of combined chemotherapy or breast cancer with recurrence within 6 months after adjuvant chemotherapy. The *NCCN guidelines*, *ESMO guidelines* and *China Breast Cancer Guidelines* all recommend nab-paclitaxel as a mono-chemotherapy for recurrent or metastatic breast cancer.

Although nab-paclitaxel has been approved as a chemotherapeutic drug for breast cancer indications at home and abroad, it has not been enlisted into *the National Medical Insurance Drug List* due to its high price. Patients enrolled in this study are required to be patients who failed ordinary paclitaxel or docetaxel treatment before enrollment. Currently, both nab-paclitaxel (domestic or imported) both can be obtained. To reduce the financial burden of patients, the nab-paclitaxel in this study is temporarily provided free of charge by Jiangsu Hengrui Pharmaceuticals Co., Ltd. (refers to Hengrui hereafter, paclitaxel has been marketed in China and has indications for breast cancer). If the drug is enlisted into *the National Medical Insurance Drug List* during this trial, Hengrui will stop donating the drug.

1.1.2.2. Information on PD-1 antibody camrelizumab (R&D code: SHR1210)

In recent years, with the deepening understanding of the human body immune system and the rapid development of biotechnology, breakthroughs have been made in tumor immunotherapy. Immunotherapy has become an important means of tumor treatment, which occupies an increasingly important position in the comprehensive tumor treatment system.

The full name of PD-1 is programmed death-1 (Programmed death-1), a negative costimulatory molecule discovered in recent years. PD-L1 and PD-L2 are the ligands of PD-1 and they can specifically bind to PD-1. Through high expression of PD-L1 molecules, tumor cells can combine with PD-1 molecules on T lymphocytes to transmit negative regulatory signals. The combination leads to the induction of apoptosis and immune incompetence of the original specific T cells. The tumor cells avoid the human's immune monitoring and killing. PD-1 inhibitor is a new kind of tumor immunotherapy drug with high profile currently. PD-1 inhibitor regulates the anti-tumor activity of T lymphocytes by blocking the PD-1/PD-L1 signaling pathway and induces tumor apoptosis.

Since 2014, because of the breakthrough efficacy of anti-PD-1 monoclonal antibody, the U.S. Food and Drug Administration (FDA) has successively approved anti-PD-1 monoclonal antibodies (Nivolumab, Bristol-Myers Squibb Company; Pembrolizumab, Merck & Co.) for the treatment of patients with advanced melanoma, non-small cell lung cancer, renal cell carcinoma, head-and-neck cancer, Hodgkin's lymphoma, hepatocellular carcinoma after sorafenib treatment, and PD-L1-positive cervical cancer. Patients who are with advanced cancer, failed standard treatment, or without an effective treatment plan are also advanced to first and second-line treatment with anti-PD-1 monoclonal antibody. In addition, due to the long-lasting efficacy and relatively mild adverse effects of anti-PD-1 monoclonal antibodies, a series of hundreds of clinical trials on advanced solid tumors and hematological malignancies have been carried out internationally, including monotherapy and combination therapy. The results of the previous trials all show a higher effective rate and long-term survival rate compared with existing therapies.

Although in recent years, breakthroughs have been made in tumor immunotherapy. However, many patients with TNBC have not achieved good effects by using anti-PD-1/PD-L1 antibody alone. Pembrolizumab (KEYNOTE 012/086/119) and Atezolizumab (NCT01633970) have undergone many monotherapy clinical studies in triple-negative breast cancer [4].

KEYNOTE-012 (NCT01848834) is a multi-center, open-label phase 1b clinical trial, that enrolled patients with PD-L1 positive advanced TNBC (The definition is tumor stroma or ≥1% tumor cells expressing PD-L1 positive by IHC detection), gastric cancer, urothelial carcinoma and head and neck cancer. This study evaluated the safety and efficacy of intravenous infusion of Pembrolizumab monoclonal antibody in patients with PD-L1 positive solid tumors. The study results of safety and efficacy in TNBC patients: the results of the detection of PD-L1 expression in 111 advanced TNBC tumor specimens showed that 65 (58.6%) tumor specimens were PD-L1 positive. Among them, 32 subjects were enrolled and evaluated for safety and tumor efficacy. Twenty-seven cases were evaluated for anti-tumor efficacy, with ORR of 18.5%, 1 case of CR (3.7%), 4 cases of PR (14.8%), 7 cases of SD (25.9%), and the median response time was 17.9 weeks (7.3-32.4 weeks). The median

duration of response did not reach (15- ≥47.3 weeks). Treatment related adverse events (TRAE) included mild arthralgia (18.8%), fatigue (18.8%), myalgia (18.8%) and nausea (15.6%). Five subjects had ≥ level 3 of TRAE, and 1 case of drug-related death.

KEYNOTE-086 Cohort B (NCT02447003) enrolled patients with PD-L1-positive advanced TNBC to evaluate the safety and efficacy of Pembrolizumab as the first-line treatment. Study results: 137 patients were screened, of which 79 (58%) were tested positive for PD-L1. A total of 52 subjects were enrolled in the group, with an ORR of 23%, 4% of CR, 19% of PR, 17% of SD, and the median response time was 8.7 weeks (8.1-17.7 weeks). The median PFS was 2.1 months (95%CI, 2.0-3.9). TRAE occurred in 37 subjects (71%), in which the most common were fatigue (31%), nausea (15%), and diarrhea (13%) .

The ORR of mono-chemotherapy in patients with PD-L1 positive advanced TNBC is about 20%, therefore, it is still necessary to explore that the identification of specific patient population that are sensitive to anti-PD-1/PD-L1 antibody drugs and the other anti-tumor drugs with different mechanisms that can be plus them to enhance the efficacy.

As of October 2018, the highest incidence of AEs during treatment in various studies is haemangioma (occurring in >30% of subjects receiving SHR-1210; included haemangioma and skin haemangioma of skin [both in preferred term]). Other common (occurring in >10% of SHR-1210 subjects) reported AE included anemia, fatigue, fever, elevated transaminases (alanine aminotransferase [ALT] and/or aspartate aminotransferase [AST]).

The most frequently reported serious adverse events (SAE) in all studies (approximately 1% of subjects) included hemangioma, lung infection, pneumonia, upper gastrointestinal (GI) bleeding, liver failure, and malignant progressive tumors. Immune-related SAE pneumonia (occurring in 1.1% of subjects) and interstitial lung disease (occurring in 0.5% of subjects) were considered adverse events related to SHR-1210. Other immune-related AEs that occurred in subjects with ≥5% SHR-1210 included elevated AST, elevated ALT, skin rash, diarrhea, and hypothyroidism.

1.1.2.3. Information on famitinib malate

In 1971, Judah Folkman first proposed the theory of tumor growth vascular dependency, pointing out that angiogenesis played an important role in the occurrence and development of tumors. Current studies have shown that tumor angiogenesis is a landmark process in the occurrence and development of solid tumors, and it is the pivotal of rapid tumor growth, invasion and metastasis. Anti-angiogenesis therapy can prevent tumor development, spread and metastasis by inhibiting tumor blood vessel formation. Among them, VEGF is a key driving factor of angiogenesis. It participates in tumor angiogenesis through various mechanisms such as mediating the proliferation, migration, infiltration of vascular endothelial cells, altering vascular permeability and expanding blood vessels. VEGFA is the most important vascular endothelial growth factor (VEGF), which has the strongest effect in inducing tumor angiogenesis. The frequency of VEGF-A protein expression and gene amplification is higher in TNBC. This suggests that anti-angiogenic drugs may be pharmaceutically active in TNBC.

Famitinib monotherapy used in breast cancer

Famitinib (famitinib malate) is a multi-target tyrosine kinase inhibitor, which has a good inhibitory activity on multiple receptor tyrosine kinases such as VEGFR2, VEGFR3, c-Kit, PDGFRβ, Flt1, Flt3, Ret, C-Src, etc. Preclinical results show that famitinib has an obvious response on a variety of human tumors transplanted in nude mice; its anti-tumor effects are superior to its similar product sunitinib in vivo and in vitro.

A phase 2 clinical trial [5] on famitinib in advanced HER2-negative breast cancer, mainly enrolled patients with HER-2 negative advanced triple-negative breast cancer who had experienced at least third-line chemotherapy regimen failure (including anthracyclines and taxanes) to evaluate the efficacy and safety of famitinib. According to the two-stage design method of clinical trial Simon for the anti-tumor drug, the sample size is designed according to the objective response rate (ORR), with null hypothesis ORR $\leq$ 5% and alternative hypothesis ORR $\geq$ 15%. Taking α=0.05, β=0.15, PASS software is used in this study, and optimization design criteria is employed. In the first stage, 25 cases will be enrolled. If 1 case achieves PR or CR, the sample will be expanded to 70 cases. If 6 out of 70 cases have responses, it indicates that further study can be carried out. Considering 10% of dropout rate, a total of 28 cases need to be enrolled in the first stage, and the second stage needs to expand the sample to 77 cases. Among the 27 patients enrolled in the first stage, 4 patients had PR in this study, and 2 patients were confirmed PR 4 weeks later, the ORR is 7.41% and DCR is 22.22%. The median progression-free survival (mPFS) was 1.93 months, suggesting that famitinib has a certain effect on patients with advanced breast cancer.

In terms of safety, the study found that the common hematological adverse reactions of famitinib were mainly manifested as white blood cell count decreased in 24 subjects (88.89%), neutrophil count decreased in 22 subjects (81.48%), platelet count decreased in 9 subjects (33.33%), hemoglobin decreased in 2 subjects (7.41%), etc. Non-hematological adverse reactions were mainly manifested in 23 subjects of proteinuria (48.15%), 21 subjects of hand-foot syndrome (77.78%), 14 subjects of hypertension (51.85%), 12 subjects of fatigue (44.44%), 6 subjects of oral ulcers (22.22%), 4 subjects of stomatitis (14.81%), 12 subjects of elevated TSH (44.44%), and 8 subjects of elevated AKP (29.63%), 7 subjects of transaminase increased (25.93%), 4 subjects of TBIL increased (14.81%), etc. These were basically common adverse reactions of similar product targeted drugs. In this study, most adverse events were mild to moderate (grade 1/2), and severe adverse events (grade 3/4) were relatively rare, while no adverse events of grade 4 occurred. The subjects were tolerable to famitinib treatment.

This study shows that famitinib monotherapy has a certain response on metastatic HER2-negative breast cancer at a tolerable dose, but the response is limited.

1.2. Scientific rationale

1.2.1. Rationale for the combination therapy

1.2.1.1. Rationale for combination therapy of anti-PD-1 antibody and chemotherapy

Chemotherapeutic drugs can lead to the death of tumor cell through cytotoxicity, besides, in recent years, more and more studies have proved that they have the effect of enhancing the body's anti-tumor immune function. Clinical practice shows that breast cancer patients (stage 2/3) who have received taxane treatment have enhanced T cell and NK cell functions. Chemotherapy kills tumor cells to release a large amount of tumor antigens, increases the amount of cross-presented tumor antigens, and then stimulates the body to produce an immune response. Combination therapy can block the activation of T cells caused by PD-1/PD-L1 inhibitors, recognize the signals of standard chemotherapy to release tumor antigens, further enhance the immune response, and kill tumor cells to benefit patients. This suggests that PD-1/PD-L1 monoclonal antibody plus standard chemotherapy is a feasible treatment strategy.

Anti-PD-1 antibody has limited single-agent efficacy in PD-L1-positive triple-negative breast cancer. Based on the relationship between the presence of TILs in breast cancer and PD-L1 over-expression, combination chemotherapy and immunotherapy have now become the hot anti-tumor strategy for triple-negative breast cancer. Currently, both Pembrolizumab (PD-1 monoclonal antibody) and Atezolizumab (PD-L1 monoclonal antibody) have been combined with chemotherapy in clinical studies of TNBC. In TNBC treatment, nab-paclitaxel can be used as the best combination chemotherapy drug because of its efficacy, safety, clinical operability, and the avoidance of immunosuppressive effects caused by steroids. The results of phase 1b study showed that in the combination therapy of Atezolizumab and nab-paclitaxel for triple-negative breast cancer not selected for PD-L1 status, 32 evaluable cases had an ORR of 38% and a 6-month DCR of 52%. Based on this result, breakthroughs have been made in the phase 3 clinical trial of Atezolizumab plus chemotherapy (NCT02425891, IMpassion130).

IMpassion130 is a multi-center, randomized, double-blind, phase 3 clinical trial [6]. It enrolled patients with advanced TNBC who had not previously received systemic anti-tumor treatment for the metastatic stage, and evaluated the efficacy, safety and pharmacokinetics of atezolizumab plus nab-paclitaxel compared with placebo plus nab-paclitaxel in the first-line treatment of patients with advanced TNBC.

In the study, patients were randomly assigned to the atezolizumab plus nab-paclitaxel treatment group or the placebo plus nab-paclitaxel control group at a ratio of 1:1. Stratification factors include whether the patient received taxane treatment during the neo-adjuvant or adjuvant stage, whether there is liver metastasis at baseline, and the expression of PD-L1 at baseline (positive vs. negative). The 2 primary endpoints of the study are PFS (evaluated according to RECIST 1.1) and overall survival (OS).

Study results: A total of 902 patients were enrolled, and 451 patients were in the treatment group and the control group respectively. The results of the intention-to-treat population (ITT)

analysis showed that the median PFS of the treatment group was 7.2 months, and that of the control group was 5.5 months (hazard ratio (HR), 0.80; 95% CI, 0.69-0.92; P = 0.002); The median OS of the treatment group was 21.3 months, and that of the control group was 17.6 months (HR 0.84; 95% CI, 0.69-1.02; P = 0.08). Analysis of PD-L1 positive subgroup data showed that the median PFS of the treatment group was 7.5 months, and that of the control group was 5.0 months (HR 0.62; 95% CI, 0.49-0.78; P<0.001); The median OS of the treatment group was 25 months, and that of the control group was 15.5 months (hazard ratio, 0.62; 95% CI, 0.45-0.86).

Based on the study results, on March 8, 2019, the FDA passed on a priority rationale the review of atezolizumab plus chemotherapy for the first-line treatment of unresectable locally advanced or metastatic PD-L1-positive triple-negative breast cancer. It also provides a strong rationale for PD-1/PD-L1 antibody drugs plus chemotherapy to treat triple-negative breast cancer.

Based on the approach of using polygenomics to accurately molecular typing of triple-negative breast cancer carried out by the team of Professor Zhimin Shao from Fudan University Shanghai Cancer Center and others, the Fudan University Shanghai Cancer Center is currently conducting a clinical trial on precision treatment of refractory triple-negative breast cancer-FUTURE study, patients with triple-negative breast cancer who failed to benefit after standard chemotherapy were accurately divided into seven different targeting arms according to their different targets. As of May 28, 2019, a total of 37 subjects have been enrolled, of which the group of IM arms (IM type: immunomodulatory) subjects (SHR1210 plus nab-paclitaxel treatment group) have enrolled 12 subjects, 7 subjects can be evaluated, and there were 3 subjects of PR (43%), 3 subjects of SD (1 subject of reduced SD) (43%), and 1 subject of PD (14%). From the current data, it can be observed that the application of the PD-1 antibody camrelizumab plus nab-paclitaxel and the selection of appropriate advantage population will further improve the efficacy of immunotherapy in combination with chemotherapy in the treatment of triple-negative breast cancer.

1.2.1.2. Rationale for combination therapy of anti-PD-1 antibody and anti-angiogenesis drugs

The VEGF and VEGFR pathways regulate the immune response by increasing DNA damage and tumor mutation burden. The VEGFR signaling pathway plays an important role in mediating tumor immune escape, and the inhibition of this pathway may enhance the activation of tumor immunity by PD-1 antibodies.

Preclinical studies have confirmed that the combination of the drugs of these 2 pathways can produce synergistic effects. The effect of camrelizumab plus apatinib on MC38 colorectal cancer was evaluated in Tg mice expressing human PD-1.

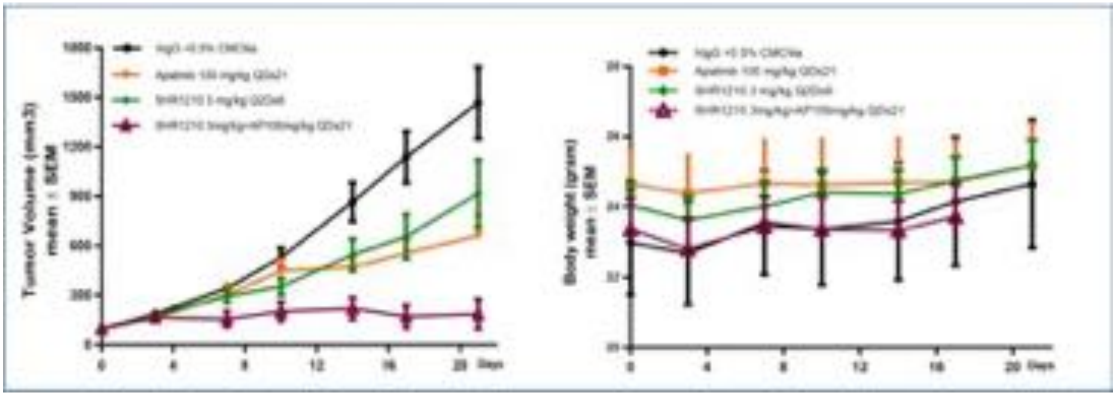

Figure 1: Assessment of the effect of camrelizumab plus apatinib on MC38 colorectal cancer in Tg mice expressing human PD-1

It is considered that SHR1210 plus apatinib may be able to improve the objective response rate of tumor while maintaining the efficacy of immunotherapy. Camrelizumab, as an immunotherapy, can achieve clinical sustained effective disease response/control (long-term efficacy). Apatinib has high tumor response rate and disease control rate (short-term efficacy).

In 2017, the top tumor translational medicine journal *Science Translational Medicine* published 2 consecutive pre-clinical studies, both of which showed that in mouse in-situ and recurrent breast cancer models, anti-angiogenesis drugs can significantly enhance the effect of anti-PD-1 or PD-L1 antibodies in inhibiting tumor growth [7] through such mechanisms as inducing the normalization of tumor blood vessels and enhancing the anti-tumor immune effect of CTL in the microenvironment of tumor.

Famitinib malate is a new multi-target tyrosine kinase receptor inhibitor independently developed by Hengrui. In addition to blocking the downstream signaling pathway of VEGFR2, it can also inhibit the activities of VEGFR3, c-Kit, PDGFRβ, Flt1, Flt3, Ret, c-Src and other receptor tyrosine kinases, and preclinical studies and in vivo experiments have proved that famitinib has even better inhibitory effect than sunitinib in some aspects, and famitinib has the dual effects of anti-tumor proliferation and inhibition of tumor tissue angiogenesis. The anti-tumor effect of famitinib as a monotherapy has been confirmed in a number of clinical studies related to solid tumors.

Currently there is "an open-labeled, multi-centered, phase 2 clinical trial on anti-PD-1 antibody SHR-1210 plus famitinib malate in the treatment of advanced urinary system tumors and gynecological tumors" funded by Hengrui. The study has been conducted in multiple institutions such as Fudan University Shanghai Cancer Center. The primary objective: To evaluate the efficacy of camrelizumab for injection plus famitinib in the treatment of advanced urinary system tumors and gynecological system tumors. The secondary objectives: To evaluate the safety and tolerance of camrelizumab for injection plus famitinib in the treatment of various tumors; to determine the pharmacokinetic properties of camrelizumab for injection plus famitinib in the treatment of various tumors in advanced stage; to obtain information about subjects producing anti-camrelizumab for injection antibody (ADA). The clinical trial is currently under recruitment.

1.2.1.3. Rationale for combination therapy of anti-angiogenesis drugs and chemotherapy

Bevacizumab is a recombinant human-derived anti-VEGF monoclonal antibody. Bevacizumab plus chemotherapy has been approved as the first-line treatment of metastatic breast cancer in many countries (more than 80 countries, except the United States). Multiple clinical studies have proved that bevacizumab plus chemotherapy can improve the existing chemotherapy protocols for the treatment of metastatic breast cancer. The key phase 3 clinical trial E2100 showed that bevacizumab plus paclitaxel had a PFS of 11.8 months compared with the PFS of 5.9 months of the mono-chemotherapy of paclitaxel (HR: 0.60; P<0.001), and the ORRs were 36.9% and 21.2% respectively (P<0.001), but there was no significant difference in OS. Two subsequent studies (AVADO and RIBBON-1) also confirmed that bevacizumab plus chemotherapy in the first-line treatment of mBC can improve PFS, but did not benefit OS. In a RIBBON-2 study evaluating bevacizumab plus second-line chemotherapy in the treatment of HER2-negative metastatic breast cancer, 159 patients with triple-negative breast cancer were included in a subgroup analysis. PFS of the bevacizumab combined chemotherapy group (6.0 months) was significantly higher than that of the mono-chemotherapy group (3.4 months), P = 0.0006; ORRs were 41% and 18% (P = 0.0078) respectively; OS has a tendency to benefit, the OSs of the combination therapy group and mono-chemotherapy group were 17.9 and 12.6 months respectively (HR 0.624, P = 0.0534).

Overall, as for the efficacy of bevacizumab plus chemotherapy in the treatment of patients with metastatic triple-negative breast cancer, the overall ORR is about 40-50%, the median PFS is 7-8 months; the median OS is 18-19 months; the 12-month OS rate is 60 to 70%. See Table 1 below for details.

Table 1: The efficacy of bevacizumab plus chemotherapy as the first-line treatment in metastatic triple-negative breast cancer

Study Name and Drug	n	ORR	Median PFS	Median OS	12-month OS rate
ATHENA Study (Paclitaxel plus Bevacizumab)	585	49%	7.2	18.3	65%
Meta-analysis (Chemotherapy plus Bevacizumab)	621	42%	8.1	18.9	71%
IMELDA Study (Capecitabine maintenance plus Bevacizumab)	46	Not reported	7.6	Not reported	90%

The above study results suggest that anti-vascular targeted drugs plus chemotherapy can improve the efficacy of mono-chemotherapy in the treatment of metastatic triple-negative breast cancer.

A “Phase 2 clinical trial of famitinib plus docetaxel versus docetaxel in the second-line treatment of advanced non-squamous and non-small cell lung cancer” funded by Hengrui was carried out in Shanghai Pulmonary Hospital. The first part of this study was a single-center, single-arm, open-label clinical trial. Three dose groups were selected for the dose escalating study, namely famitinib 15mg plus docetaxel 60mg/m^2, famitinib 20 mg plus docetaxel 60mg/m^2 and famitinib 25mg plus docetaxel 60mg/m^2, 3 to 6 subjects were enrolled in each

dose group, and the DLT observation period was within the first cycle after the medication starts. According to the safety, pharmacokinetics and preliminary efficacy results of the 3 dose groups, the dose of famitinib in the second part of study was determined, and at the same time, the number of subjects in this dose group was expanded to 10 cases. The study is currently in the follow-up period.

During the dose escalating stage, 3 subjects were enrolled in each of the 3 dose groups, and no DLT was observed in the first cycle, namely the core phase. There were effective cases in the 20mg group and 25mg group. The preliminary pharmacokinetic results showed that there was no drug interaction between famitinib and docetaxel. Considering that there is some overlap between the common adverse reactions of famitinib and docetaxel, the dose of famitinib 20mg is recommended for the second part of study.

A total of 18 subjects were enrolled in the first phase, 3 in the 15 mg group, 11 in the 20 mg group, and 4 in the 25 mg group. The tolerance results showed that all the 18 subjects experienced AEs, 6 subjects experienced SAEs, 1 case was definitely related (neutropenia), and 5 cases were definitely not related (progressive disease). The key hematological adverse events were neutrophil counts decreased, white blood cell counts decreased, hemoglobin decreased, and platelet counts decreased; key non-hematological adverse events were proteinuria, hand-foot syndrome, elevated alanine aminotransferase, elevated aspartate aminotransferase, fatigue, and nausea. The main grade 3 to 4 adverse events were neutrophil counts decreased, white blood cell counts decreased, proteinuria, and hand-foot syndrome. Most of the adverse events were mild to moderate events, and no unexpected adverse events occurred. The efficacy results showed that in 2 cycles of efficacy assessment, there were 5 cases of PR and 11 cases of SD. At present, all subjects have been out of the group and are currently in the follow-up period.

Famitinib is a multi-target tyrosine receptor kinase inhibitor. The clinical trial of its combination with chemotherapy in the treatment of non-small cell lung cancer provides a rationale for its combination with chemotherapy in the treatment of triple-negative breast cancer.

1.2.2. Design rationale for triple-agent combination regimen

1.2.2.1. Rationale for the triple-agent combination therapy of camrelizumab, nab-paclitaxel and famitinib

Combined immunotherapy is currently the most popular study field. A number of studies have confirmed that the combination of anti-PD-1/PD-L1 antibodies with other immunomodulators, chemotherapy, and molecular targeting can significantly improve the efficacy of immunotherapy.

It can be seen from the above-mentioned studies that nab-paclitaxel is currently one of the standard monotherapies for the treatment of metastatic triple-negative breast cancer recommended by Chinese and foreign guidelines. Although the mono-chemotherapy of VEGFR tyrosine kinase inhibitor famitinib and anti-PD-1/PD-L1 antibody is effective to some extent in the treatment of metastatic triple-negative breast cancer, their efficacy is limited.

Based on the results of phase 3 studies abroad, the anti-PD-L1 antibody atezolizumab plus nab-paclitaxel has been approved in the United States to treat PD-L1-positive metastatic triple-negative breast cancer, providing a rationale for the prospects of efficacy of the combination therapy of similar drugs in our country. However, only 18% of subjects in this study are Asians, Chinese patients' data on the use of this treatment protocol is limited. At the same time, considering the availability of drugs, to meet the domestic clinical needs of TNBC, it is still necessary to explore how to further enhance the efficacy of the combination therapy of PD-1/PD-L1 antibody and nab-paclitaxel and to select its advantage benefit population.

Consider the synergistic effect of anti-vascular targeted drugs plus immunotherapy and chemotherapy. At present, international studies of bevacizumab plus PD-L1 antibody atezolizumab and chemotherapy have been carried out for tumor of multiple types. The IMpower150 study published in the *New England Journal of Medicine* in June 2018 is a randomized, controlled phase 3 study of first-line treatment for patients with metastatic non-small cell lung cancer (NSCLC), comparing the efficacy and safety of atezolizumab plus chemotherapy (carboplatin plus paclitaxel) ± Bevacizumab and bevacizumab plus chemotherapy. Patients were randomly assigned by a ratio of 1: 1: 1 to receive atezolizumab plus carboplatin plus paclitaxel (ACP group: 402 patients), or atezolizumab plus bevacizumab plus carboplatin plus paclitaxel (ABCP group: 400 patients) or bevacizumab plus carboplatin plus paclitaxel (BCP group: 400 patients). Randomization stratification factors include gender, presence of liver metastases and PD-L1 expression at baseline, (assessed by immunohistochemical analysis). In the primary endpoint analysis, the ABCP group was compared with the BCP group, and then the ACP group with the BCP group. The results showed that the PFS of the ABCP group was significantly longer than that of the BCP group (median PFS: 8.3 months vs. 6.8 months; HR=0.62, 95% CI=0.52-0.74; P<0.001). The 6-month PFS rate of the ABCP group was higher than that of the BCP group (66.9% vs. 56.1%); the 12-month PFS rates were 36.5% and 18.0% respectively.

In terms of safety, safety analysis was included in this study (393 patients received ABCP and 394 received BCP). The incidence of adverse events related to any treatment was 94.4% in the ABCP group and 95.4% in the BCP group. The incidence of grade 1 or 2 treatment-related adverse events was 35.9% in the ABCP group and 45.4% in the BCP group. The most common grade 3/4 treatment-related adverse events were neutropenia, decreased neutrophil counts, febrile neutropenia, and hypertension. There were 11 cases (2.8%) of treatment-related deaths in the ABCP group and 9 cases (2.3%) in the BCP group. The total incidence of immune-related adverse events in the ABCP group was 77.4%, and the most common immune-related adverse events were skin rash, hepatitis, hypothyroidism, hyperthyroidism, pneumonia, and colitis.

IMpower150 is the first phase 3 study to explore the efficacy and safety of immune checkpoint inhibitors plus anti-angiogenesis plus chemotherapy for the first-line treatment of advanced non-squamous NSCLC [8]. The results showed that compared with bevacizumab plus carboplatin plus paclitaxel, atezolizumab plus bevacizumab plus carboplatin plus paclitaxel has shown benefits for both PFS and OS, providing a new treatment option for the first-line treatment of advanced non-squamous NSCLC. In addition, the clinical trial protocol is well tolerated, which is similar to previously reported safety data.

The phase 3 study of bevacizumab plus PD-L1 antibody atezolizumab and chemotherapy (ABCP protocol) in the treatment of ovarian cancer is underway.

Based on the study experience of this combination in the treatment of other types of tumor, as well as the safety and preliminary efficacy of previous studies of immunotherapy plus chemotherapy, immunotherapy plus anti-vascular targeted drugs, and anti-vascular targeted drugs plus chemotherapy in the treatment of triple-negative breast cancer, we have the reason to assume that the combination of the anti-vascular targeted drug famitinib, PD-1 antibody camrelizumab and chemotherapy can further improve the treatment efficacy for advanced triple-negative immunomodulatory population while ensuring safety and tolerance.

For the treatment of recurrent and metastatic triple-negative breast cancer, a type of refractory tumor, rare breakthroughs have been made in this field for many years, it is the current major pursuit to find the most likely beneficial treatment for patients with immunomodulatory subtypes of triple-negative breast cancer through a triple-agent combination therapy (camrelizumab, nab-paclitaxel, famitinib) on the rationale of preliminary studies.

This study is an exploratory clinical trial. Based on the molecular typing, it recommends a theoretically suitable individualized treatment protocol. No trial data has been published using similar treatment protocols. Theoretically, the control group should be a population of the same molecular typing and adopt the standard of care or the treatment protocol selected by the doctor, but not people of different molecular typing adopting the same protocol; people of different molecular typing should adopt corresponding individualized treatment protocol, which can theoretically better benefit this group of people. If people of different molecular typing are set as a control group and the trial protocol is adopted, the possibility of benefit is low, and it may also bring more risks to patients.

1.2.2.2. Dose rationale for combination therapy of camrelizumab, nab-paclitaxel and famitinib

1.2.2.2.1. Dose selection rationale for combination therapy of camrelizumab and anti-vascular targeted drugs

In this study, the initial dose of camrelizumab in combination therapy was set as 200 mg Q2W intravenously, which was the recommended dose in its instructions.

1.2.2.2.2. Dose selection rationale for famitinib plus chemotherapy

A “phase 2 clinical trial on famitinib plus docetaxel versus docetaxel in the second-line treatment of advanced non-squamous and non-small cell lung cancer” was carried out by Shanghai Pulmonary Hospital. The first stage of this trial was a single-center, single-arm, open-label clinical trial, 3 dose groups were selected for dose escalating study, namely famitinib 15 mg plus docetaxel 60 mg/m^2, famitinib 20 mg plus docetaxel 60 mg/m^2 and famitinib 25 mg plus docetaxel 60 mg/m^2, 3 to 6 subjects were enrolled in each dose group, and the DLT observation period was within the first cycle after starting the medication. According to the safety, pharmacokinetics, and preliminary efficacy results of the 3 dose groups, the dose of famitinib in the second stage was determined, and at the same time, the number of subjects in this dose group was expanded to 10. The trial is currently in the

follow-up period.

During the dose escalating stage, 3 subjects were enrolled in each of the 3 dose groups, and no DLT was observed in the first cycle, namely the core phase. There were effective cases in both the 20mg group and the 25mg group. The preliminary pharmacokinetic results showed that there was no drug interaction between famitinib and docetaxel. Considering that there is some overlap between the common adverse reactions of famitinib and docetaxel, the dose of famitinib 20mg was recommended for the second phase.

A total of 18 subjects were enrolled in the first phase, 3 in the 15 mg group, 11 in the 20 mg group, and 4 in the 25 mg group. The tolerance results showed that 18 subjects had AEs, 6 subjects had SAEs, 1 case was definitely related (neutropenia), and 5 cases were definitely not related (disease progression). The key hematological adverse events were neutrophil counts decreased, white blood cell counts decreased, hemoglobin decreased, and platelet counts decreased; key non-hematological adverse events were proteinuria, hand-foot syndrome, elevated alanine aminotransferase, elevated aspartate aminotransferase, fatigue and nausea. Key grade 3 to 4 adverse events were neutrophil counts decreased, white blood cell counts decreased, proteinuria and hand-foot syndrome. Most of the adverse events were mild to moderate events, and no unexpected adverse events occurred. The efficacy results showed that in 2 cycles of efficacy assessment, there were 5 cases of PR and 11 cases of SD. At present, all subjects have been out of the group and are currently in the follow-up period.

The above trial provides data support for the starting dose of famitinib plus chemotherapy, which is set to 20 mg qd, given in continuous orally.

1.2.2.2.3. Selection rationale for dosage of nab-paclitaxel

As mentioned earlier, in the pivotal phase 3 clinical trial for nab-paclitaxel, for patients with advanced disease who had not used paclitaxel or had used paclitaxel during previous adjuvant treatment stage but the time to recurrence is longer than 1 year, that is, “advanced first-line” patients, compared with the paclitaxel group, the objective response rate on the primary endpoint in the nab-paclitaxel group was significantly improved, which shows the efficacy superiority of nab-paclitaxel in mono-chemotherapy. nab-paclitaxel is recommended by NCCN, ESMO and China Breast Cancer Guidelines to be used as a mono-therapeutic drug for recurrent or metastatic breast cancer, respectively.

Choosing nab-paclitaxel can ① avoid the immunosuppressive effects caused by the corticosteroids necessarily used when the anti-PD-1 antibody is used in combination with other taxanes; ② theoretically, compared to other taxanes, nab-paclitaxel has better efficacy, and the tumor antigens released by killing tumor cells increases. Moreover, the nab-paclitaxel can jointly stimulate PD-1 inhibitor camrelizumab for the immune response, and co-stimulate and activate tumor-specific T cells, as well as prolong duration of these activities.

Meanwhile, refer to the drug selection of previous phase 3 clinical trial design on PD-1/PD-L1 antibody combined with chemotherapy in patients with triple-negative breast cancer.

Study	Treatment Regimen
IMpassion130	Treatment group: atezolizumab 840 mg q2w + paclitaxel (albumin bound, 100

	mg/m², 3 weeks/discontinuation for 1 week)<br>Control group: placebo 840 mg q2w + paclitaxel (albumin bound, 100 mg/m², 3 weeks/ discontinuation for 1 week)
KEYNOTE-355	Treatment group: pembrolizumab 200 mg q3w + chemotherapy (including paclitaxel (albumin bound, 100 mg/m², 3 weeks/ discontinuation for 1 week)<br>Control group: placebo 200 mg q3w + chemotherapy (including paclitaxel (albumin bound, 100 mg/m², 3 weeks/ discontinuation for 1 week)

In this study, the combined chemotherapeutic drug and standard single-agent control group were nab-paclitaxel. The considerations in dose selection are as follows. The FDA and NMPA have approved the q3w administration regimen of abraxane and Ai Yue® 260 mg/m² respectively. However, in clinical practices, the weekly administration regimen has better tolerability and efficacy than the q3w protocol, so the former type of administration regimen is more recommended.

A phase 2 randomized study of nab-paclitaxel in the first-line treatment of advanced breast cancer showed the superiorities of weekly administration: in this study, there were 4 groups, nab-paclitaxel 300 mg/m² q3w, weekly 100 mg/m², weekly 150 mg/m², and docetaxel 100 mg/m² q3w, a total of 302 patients were enrolled. The ORR of the 300 mg/m² q3w group (the first day of every 3 weeks) was 37%, the ORR of 100 mg/m² weekly administration (3 weeks/discontinuation for 1 week) was 45%, and the ORR of the 150 mg/m² weekly administration (3 weeks/discontinuation for 1 week) was 49%, and the ORR of the docetaxel 100 mg/m² q3w group (the first day of every 3 weeks) was 35%. The PFS were 11.0 months, 12.8 months, 12.9 months and 7.5 months, respectively. And under the same mode of administration, there was no significant difference in PFS and ORR between 100 mg/m² and 150 mg/m², but compared with patients treated with 100 mg/m², there was a significant higher proportion of patients treated with 150 mg/m² who had grade 3 or 4 neutropenia (44% vs 25%) and grade 3 sensory nerve disorders (14% vs 8%).

Subsequent clinical studies have also shown that under weekly administration, a dose exceeding 100 mg/m² does not bring more efficacy benefits, but causes more serious toxicities and side effects due to high doses. CALGB 40502 is a phase 3 randomized study in naïve chemotherapy patients with HER2-negative advanced breast cancer, and now they received bevacizumab in combination with different chemotherapeutic drugs, including paclitaxel, paclitaxel (albumin bound, 150mg/m², weekly administration) and ixabepilone. In this study, patients could not tolerate the weekly administration of 150 mg/m² of nab-paclitaxel, and the PFS did not increase compared to paclitaxel. The conclusion was "150 mg/m² of nab-paclitaxel should not be used". The NCCN guidelines states that the weekly dose of nab-paclitaxel should not exceed 125 mg/m². In summary, considering the efficacy and safety, the appropriate dose and route of administration of nab-paclitaxel is 100 mg/m², weekly dose (3 weeks/discontinuation for 1 week).

Therefore, we choose the fixed dose of camrelizumab 200 mg Q2W intravenously, famitinib 20 mg qd in oral route, and nab-paclitaxel 100 mg/m² intravenously, administered weekly (3 weeks/discontinuation for 1 week) as the initial dose of triple-drug combination therapy.

1.3 Potential risks and benefits

1.3.1 Known potential risks

nab-paclitaxel has been marketed already, and its adverse reactions include myelosuppression (mainly neutropenia) which is dose-dependent and dose-limiting toxicity, peripheral neurotoxicity, liver dysfunction, allergic reactions, etc. In various studies, camrelizumab, as a newly-marketed PD-1 drug, the AE (TEAE) with the highest incidence rate during treatment (>30%, camrelizumab alone or combination therapy) is the reactive capillary endothelial proliferation (RCEP) (59.5%). Other common TEAEs (>10%) include hypothyroidism, constipation, diarrhea, nausea, fatigue, fever, alanine aminotransferase (ALT) increased, aspartate aminotransferase (AST) increased, blood bilirubin increased, neutrophil count decreased, platelet count decreased, white blood cell count decreased, decreased appetite, proteinuria, and cough. The most frequently reported serious adverse events (SAEs) in all studies (≥1% of subjects) are abnormal liver function, lung infections, platelet count decreased, neoplasm progression, and lung inflammation. Immune-related SAE lung inflammation (occurred in 1.6% of subjects) and interstitial lung disease (occurred in 0.5% of subjects) are considered to be camrelizumab-related adverse events. Other immune-related TEAEs include AST increased, ALT increased, rash, diarrhea, and hypothyroidism. Famitinib is a small molecule anti-tumor angiogenesis targeted drug that is currently in clinical trials. Common adverse events in current clinical trials include myelosuppression, proteinuria, hand-foot syndrome, hypertension, fatigue, oral ulcers, and thyroid dysfunction, etc., but most of the adverse events are mild to moderate (grade I/II), severe adverse events (grade III /IV) are relatively rare. Compared with monotherapy, the triple-drug combination therapy in this clinical trial may even lead to more obvious toxic and side effects.

1.3.2 Known possible benefits

At present, the key treatment for advanced triple-negative breast cancer is still chemotherapy. It is particularly important to develop effective treatments such as targeted therapy and immunotherapy. The nab-paclitaxel, camrelizumab and famitinib in this study have all achieved certain effects in clinical trials of breast cancer and other tumors. Combining triple anti-tumor drugs with different mechanisms of action may better control the tumor conditions of patients with advanced triple-negative breast cancer to a certain extent, and even bring survival benefits. Moreover, this study selected the immunomodulatory population among triple-negative breast cancer patients as subjects, this part of patients may be more sensitive to PD-1 antibody treatment, and the survival benefits may be more obvious.

2. Study objectives and endpoints

2.1 Study objectives

2.1.1 Primary objective

To evaluate the objective response rate (ORR) of PD-1 antibody camrelizumab combined with nab-paclitaxel plus famitinib as the first-line treatment in patients with unresectable locally advanced or metastatic triple-negative immunomodulatory breast cancer.

2.1.2 Secondary objectives

To evaluate the progression-free survival (PFS), overall survival (OS), duration of objective response (DoR) and disease control rate (DCR) of PD-1 antibody camrelizumab in combination with nab-paclitaxel plus famitinib as the first-line treatment in patients with unresectable locally advanced or metastatic triple-negative immunomodulatory breast cancer;

To evaluate the safety of PD-1 antibody camrelizumab plus nab-paclitaxel and famitinib as a first-line treatment in patients with unresectable locally advanced or metastatic triple-negative immunomodulatory breast cancer;

To evaluate the pharmacokinetics (PK) characteristics of PD-1 antibody camrelizumab plus nab-paclitaxel plus famitinib as a first-line therapy in patients with unresectable locally advanced or metastatic triple-negative immunomodulatory breast cancer;

To evaluate the immunogenicity of PD-1 antibody camrelizumab plus nab-paclitaxel and famitinib as the first-line treatment in patients with unresectable locally advanced or metastatic triple-negative immunomodulatory breast cancer.

To assess predictive and prognostic exploratory biomarkers in archival and/or fresh tumor tissue and blood and their association with disease status and/or response to study treatment.

2.2 Study endpoints

2.2.1 Primary endpoint

Objective response rate (ORR).

2.2.2 Secondary endpoints

PFS

OS

DCR

DoR

Safety endpoints: The incidence and grade of AE and SAE, AE is assessed according to NCI-CTC AE 5.0; changes in the following indicators compared to their corresponding baseline: ECOG performance status, vital signs, physical examination, laboratory examination indicators (hematology, urinalysis, stool routine test, blood biochemistry), 12-lead electrocardiogram (ECG), echocardiogram, etc.

The PK parameters of the PD-1 antibody camrelizumab plus nab-paclitaxel and famitinib.

The proportion of subjects who developed anti-SHR-1210 antibody and their antibody levels.

The predictive and prognostic exploratory biomarkers in archival and/or fresh tumor tissue and blood and their association with disease status and/or response to study treatment.

3. Study design

3.1 Overall design

This study is an open-label, single-arm, phase 2 clinical trial.

It is planned to enroll 46 patients with inoperable locally advanced or metastatic triple-negative immunomodulatory breast cancer who have not used paclitaxel or have used paclitaxel drugs in the previous adjuvant/neo-adjuvant treatment stage but the time to recurrence is longer than 6 months. They have not received systemic anti-tumor therapy at this advanced stage, and the immunomodulatory type is defined as the proportion of CD8+ cells detected by immunohistochemistry no less than 10%. To ensure that there are at least 41 subjects who could be viewed as evaluable patients (estimated at a dropout rate of approximately 10%). After confirmation of enrollment, the anti-PD-1 antibody camrelizumab was administered: 200 mg intravenously, Q2W, every 4 weeks as a treatment cycle; nab-paclitaxel: 100 mg/m² intravenously, once a week, three consecutive weeks and discontinuation for one week, every 4 weeks as a treatment cycle; the starting dose of famitinib malate: 20 mg qd in oral route, it can be reduced to 15 mg according to tolerance, and 4 consecutive weeks as a treatment cycle. Among them, nab-paclitaxel should be used for at least 6 cycles, and there is no limit on the number of treatment cycles. The triple-drug combination therapy must be terminated when the disease progresses, the toxicity is intolerable, the informed consent is withdrawn or it must be terminated according to the investigator's judges (whichever occurs first). After the subject finishes the treatment, it is necessary to continue to receive safety follow-up until 28 days after the last administration, all adverse events return to within grade I, or all adverse events are clinically stable (whichever is achieved later); subjects out of group for non-PD or non-death causes need to receive efficacy follow-up until PD, the beginning of other anti-tumor drug treatment or death (whichever occurs first); all subjects will receive survival follow-up (OS data collection) until death, loss to follow-up or discontinuation of the trial (whichever occurs first).

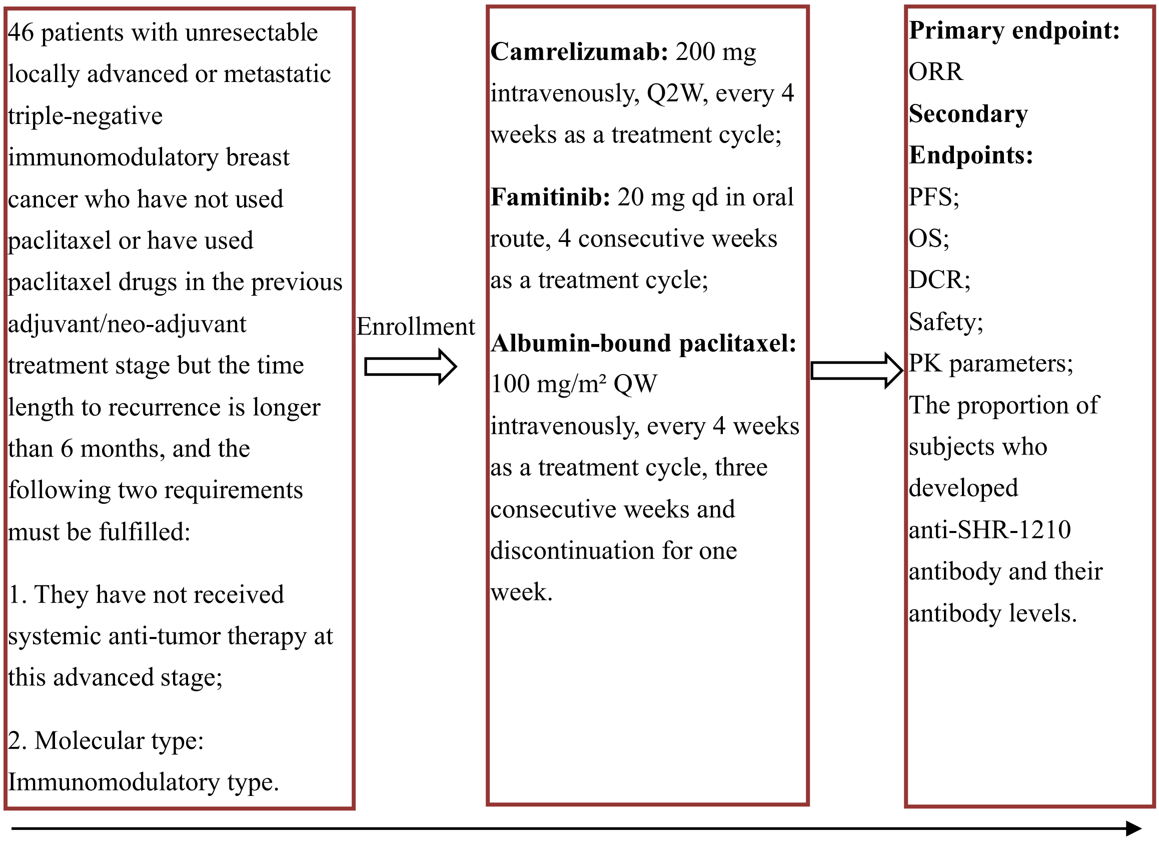

The assessment will be conducted every 2 cycles (8 weeks) until disease progression or discontinuation from the trial.

3.2 Measures to minimize bias

This is a single-arm, open-label, non-randomized, and unblinded study.

4. Subject's screening and withdraw

4.1 Inclusion criteria

Patients must meet all the following inclusion criteria to be enrolled in this trial:

1. Age: ≥18 years old and ≤70 years old;

2. ECOG status of grade 0～1;

3. The expected survival period must be at least 3 months;

4. Advanced triple-negative invasive breast cancer is confirmed by pathological examination, as well as meets the following conditions:

The definition of triple-negative breast cancer (TNBC) refers to the breast cancer with estrogen receptor (ER), progesterone receptor (PR) and human epidermal growth factor receptor 2 (HER-2) all negative in pathological examination. Specifically: ER negative: IHC<1%, PR negative: IHC<1%, HER2 negative: IHC-/+ or IHC++ but FISH/CISH negative. All specimens need to be verified by the

Pathology Department of Fudan University Shanghai Cancer Center , and patients with recurrence and metastasis must have their molecular classification re-examined;

Tumor staging: recurrent or metastatic breast cancer; patients with local recurrence must be confirmed by the investigator that they cannot receive radical surgical resection;

Definition of immunomodulatory type: The proportion of CD8+ cells is ≥10% in IHC staining analysis.

5. Patients who have never received taxanes treatment or received taxanes treatment as adjuvant/neoadjuvant therapy but the interval between the end of treatment and relapse is greater than 6 months; or have not received systemic anti-tumor treatment (chemotherapy, targeted therapy, etc.) in the advanced stage;

6. At least one extracranial target lesion with measurable diameter can be identified based on imaging judgment. (RECIST 1.1 standard);

7. The main organs are functioning well, namely the relevant examinations meet the following requirements within 7 days before the first administration:

1) Routine blood test

ANC≥1.5×10^9/L;

PLT≥90×10^9/L;

Hb≥90 g/L;

2) Blood chemistry test

TBIL≤1.5×ULN;

Serum cholesterol ≤ 1.25 x ULN; triglyceride ≤ 2.0 x ULN;

ALT and AST≤2.5×ULN; for patients with liver metastasis lesions, ALT and AST≤5×ULN;

BUN and Cr≤1.5×ULN and creatinine clearance ≥50 mL/min (formula Cockcroft-Gault);

PT and APTT ≤ 1.5 x ULN

3) Echocardiography

LVEF ≥50%;

4) 12-lead electrocardiogram

QT interval corrected by Fridericia method (QTcF) for males <450 ms, females <470 ms;

8. Patients join this study voluntarily, sign the informed consent form, with good

compliance and are also cooperative during follow-up.

4.2 Exclusion criteria

Patients with any of the following situations will not be included in this study:

1. Patients who have received taxanes treatment as adjuvant/neoadjuvant therapy, but the interval between the end of paclitaxel treatment and recurrence and metastasis were <6 months;

2. Patients who have previously received treatment (except bevacizumab) with VEGFR class of small molecule tyrosine kinase inhibitors (such as famitinib, sorafenib, sunitinib, regorafenib, etc.);

3. Patients with a medical history of hemorrhage, and any severe bleeding event that reached grade 3 or above in CTCAE 5.0 within 4 weeks before screening;

4. Imaging result shows that the tumor has invaded the periphery of important blood vessels or it is highly possible that the tumor will invade important blood vessels and cause fatal hemorrhage during treatment at investigator's discretion;

5. Patients with abnormal coagulation function and bleeding tendency (14 days before randomization, the patient must meet the standard: INR is within the normal range without anticoagulant); patients that are treated with anticoagulant or vitamin K antagonist such as warfarin, heparin or its analogues; under the premise that the prothrombin time International Normalized Ratio (INR, International Normalized Ratio) ≤1.5, the use of low-dose warfarin (1 mg orally, once daily) or low-dose aspirin (not exceed 100 mg/day) is permitted for the purpose of prevention;

6. Patients with arteriovenous thrombosis events occurred within one year before screening, such as cerebrovascular accidents (including transient ischemic attack), deep vein thrombosis (except those who recovered, as confirmed by the investigator, from venous thrombosis caused by intravenous catheterization due to pre-chemotherapy) and pulmonary embolism, etc.;

7. Patients with a history of autoimmune diseases or those using glucocorticoids or immunosuppressive drugs;

8. Patients with swallowing inability, chronic diarrhea and intestinal obstruction, or with other conditions that affect the administration and absorption of medications;

9. Patients with a third space effusion that cannot be controlled by drainage or other methods (such as large amounts of pleural fluid and ascites);

10. Patients that have received radiotherapy, chemotherapy, surgery or other targeted and immunotherapy for advanced triple-negative breast cancer within 4 weeks before receiving the first study treatment;

11. Patients that have not recovered from the AEs of the previous medication before receiving the first treatment at investigator's discretion (NCI-CTCAE version 5.0 classification > grade 1);

12. Patients with untreated central nervous system (CNS) disease (patients with treated, asymptomatic treated CNS metastases also eligible.);

13. Patients that participated in other anti-tumor drug clinical trials within 4 weeks before receiving the first administration;

14. Patients with long-term unhealed wounds or fractures with incomplete healing;

15. Patients whose urinalysis showed that the patient's urine protein ≥2+ and the 24h urine protein quantitative > 1 g was confirmed;

16. Patients with past or current history of pulmonary fibrosis, interstitial pneumonia, pneumoconiosis, radiation pneumonia, drug-related pneumonia, or severely impaired lung function.

17. Patients with known active stage of HBV or HCV infection or hepatitis B DNA ≥500, or patients with chronic abnormal liver function;

18. Patients who suffered from other malignant tumors in the past 5 years, excluding cured cervical carcinoma in situ, skin basal cell carcinoma or skin squamous cell carcinoma;

19. Patients with allergies, or those who are known of having a medical history of allergies to any drug components of this study; or those who are allergic to other monoclonal antibodies;

20. Patients with active infections or those who have been treated with systemic immune stimulating factors within 4 weeks before enrollment;

21. Patients with a history of immunodeficiency, including HIV positive, or have other acquired or congenital immunodeficiency diseases, or have a history of organ transplantation, or positive syphilis antibody test;

22. For female subjects: patients that are non-surgical sterilized or non-menopausal refused to use a medically approved contraceptive method during the study treatment period and within 6 months after the end of the study treatment; women of childbearing age are positive in serum or urine pregnancy test within 7 days before enrolled in this study or in lactating period.

23. Patients with hypertension that cannot be controlled by a single antihypertensive drug (systolic blood pressure> 140 mmHg, diastolic blood pressure> 90 mmHg); those with a history of unstable angina; whose who are newly diagnosed with angina within 3 months before screening or with a myocardial infarction occurrence within 6 months before screening; patients with arrhythmia (including QTcF: male ≥450 ms, female ≥470 ms) that requires long-term use of anti-arrhythmic drugs or with cardiac insufficiency that ≥ grade 2in New York Heart Association grading;

24. Patients with a history of abnormal thyroid function;

25. Patients with grade ≥ 2 peripheral neuropathy;

26. Patients with a clear history of neurological or mental disorders, including epilepsy

or dementia;

27. Patients with any other conditions that is considered not suitable to participate in this study at investigator's discretion.

4.3. Lifestyle requirements

4.3.1. Contraception

In this study, nab-paclitaxel, camrelizumab and famitinib were suspected of reproductive toxicity, but have not been confirmed in clinical use. All fertile female subjects who will use the above-mentioned study drugs, if the investigator believes that they are at risk of pregnancy, then they must use at least two effective methods of contraception. The contraception period will last the entire treatment, begins from the signing of the informed consent form and ends 7 months after the last dose of the test drug. The investigator or his designated personnel negotiates with the subject to choose two suitable contraceptive methods from the following options, and confirms that the subject knows how to use contraceptive methods in a correct and continuous way. In addition, the subject needs to know: once the chosen contraceptive method is discontinued, the investigator should be notified immediately.

4.4. Subject's withdrawal from the study or discontinuation of the treatment

4.4.1. Withdrawal from the study

The subject can voluntarily withdraw from the trial at any time, or may be required to withdraw from the trial due to safety or behavioral reasons, or unable to comply with the study visit schedule or procedures required by the protocol of clinical study center where he or she belongs to.

Reasons for the subject's withdrawal from the study may include:

The subject withdraws the informed consent to participate in the study and refuses further followed up;

Clinical adverse events, abnormalities values of laboratory tests or complicated diseases, and the investigator believes that continuing to participate in the trial is not in the best interests for the subjects;

Other situations leading to the investigator's determination that it is necessary for the subject to withdraw study, such as the subject's loss of ability to express his will freely due to imprisonment or isolation;

Lost to follow-up;

The subject's death;

The investigator terminated the study.

The reason for the subject's withdrawal must be recorded in the case report form and the subject's medical record.

It should be noted that the withdrawal of informed consent means that the subject has

withdrawn the consent for further contact, or no longer agrees to the previously authorized person to provide further information. Whenever possible, the subject should notify the investigator in written notice that he has decided not to receive followed up. The investigator should make every effort to explain and record the withdrawal of informed consent, and clarify the withdrawal is whether it is not willing to take the study drug, or not willing to be followed up by the visit specified in the protocol at the same time.

4.4.2. Criteria for discontinuation of study treatment

Discontinuation of study treatment does not mean withdrawal from the study. Subjects who terminate the treatment must complete the remaining study visits as required by the protocol. If the subject meets any of the following criteria, the treatment must be terminated:

The subject requests the discontinuation of test drug treatment;

Medical imaging or clinical features indicate disease progression;

Pregnancy of the subject during the study;

The occurrence of any clinical adverse events, abnormalities in laboratory tests or other medical conditions that may cause the subjects no longer benefit from subsequent treatment;

Overall deterioration of health status, unable to continue participating in the trial;

Significant deviations from the protocol after enrollment, such as unqualified and non-compliant subjects, etc.;

Lost to follow-up;

The investigator terminates the study;

The subject’s death;

Other reasons leading to the investigator’s determination that it is impossible to continue the treatment.

4.4.3. Steps to withdraw or discontinue study treatment

The patient must do his/her best to complete the efficacy and safety examinations specified in the protocol when he/she withdraws from the trial, to complete the safety follow-up visit and subsequent survival follow-up, and to fully record the adverse events (AE) and outcome. The investigator can suggest or provide new or alternative treatment methods to the subject according to his/her actual situation. Patients with non-progressive disease should continue to be followed up for imaging assessment until the subject starts new anti-tumor therapy or the disease progression.

If the subject refuses to go to the clinical research center for further visits, the survival status should continue to be tracked unless the subject withdraws consent to disclose further information or be contacted. In this case, no study assessment should be conducted, and no information should be collected. The investigator can keep and continue to use all data collected before the subject withdraws his/her informed consent, unless the subject requests

that the above-mentioned collected information be also withdrawn.

4.5. Definition of the end of study

1 year after the enrollment of the last subject.

The time the investigator decides to terminate the study.

5. Investigational product management

5.1. Investigational product delivery

This study was conducted in the Department of Breast Surgery, Fudan University Shanghai Cancer Center. The subjects would be automatically assigned a subject number after they signed a written informed consent form and completed necessary baseline assessments. The corresponding investigational products will be delivered according to the drug number. The camrelizumab, nab-paclitaxel and famitinib in this project will be all provided by Hengrui (unified management in accordance with hospital regulations). After completing the screening process, they will be formally enrolled in the study. The drug delivery should be strictly managed. Special persons in this hospital will be appointed to be responsible for keeping and filling in the receipt and use records. The remaining drugs and empty bottles shall be recovered in time during the trial. It is necessary to regularly check the use and record of drugs, and deal with the recovery at any time.

5.2. Route of administration

Camrelizumab: 200 mg, intravenously, D1, once every two weeks, every 4 weeks as a treatment cycle;

nab-paclitaxel: 100 mg/m^2, intravenously, D1, once a week, three consecutive weeks and discontinuation for one week, every 4 weeks as a treatment cycle;

Famitinib malate: 20 mg, once a day, in oral route, which can be reduced to 15 mg according to tolerance, 4 consecutive weeks as a treatment cycle.

5.3. Drug storage and management

Investigational products will be uniformly stored, delivered, and recycled by the testing unit as per the requirement of GCP. The study drugs should be stored at room temperature, sealed and protected from light, and the shelf life is temporarily set as 2 years.

6. Study procedures

6.1. Screening

This study allows re-screening of subjects who have failed the previous screening. When re-screening, they must re-sign the informed consent form and re-register to obtain a new subject number.

The screening period starts with the signing of the informed consent form and ends with the first study medication or screening failure.

Subjects must sign an informed consent form before proceeding to the screening procedures

specified in this study. Laboratory examinations results and imaging assessments required for routine clinical treatment before signing the informed consent form can be used if they are within the specified window period and meet the needs of this study.

Unless otherwise specified, the following screening procedures must be completed within 28 days before starting the treatment (see “Schedule of Activities” for details):

Obtain written informed consent form signed by the subject;

Demographic data: Gender, date of birth, nationality, height, weight, etc.;

Tumor diagnosis: Primary and recurrent lesions, date of pathological diagnosis of metastases, pathological type, pathological staging (TNM), clinical tumor staging, number of metastasis lesions, and date of disease progression or recurrence after the last treatment. If the recurrence lesion is a single measurable target lesion, the selection must be based on the pathological results of recurrence lesions. If it is difficult to obtain materials for the recurrence or metastasis lesions, the pathological results of the primary lesion can be used;

Tumor treatment history:

Tumor surgery history: name of surgery, date of surgery;

Radiotherapy history: radiotherapy site, dosage, start date and end date

Neo-adjuvant treatment history: treatment regimen, cycle, start and end time, etc.;

Adjuvant treatment history: treatment regimen cycle, start and end time, etc.;

Treatment history at advanced stage: treatment regimen, cycle, start time and end time, etc.;

History of drug menopause: drug name, dose, start and end time, etc.;

Concurrent medical history, prior medication history, history of drug allergy, etc.;

Tumor imaging examination: The screening period includes at least CT or MRI of the chest, abdomen and brain. Bone scans should be performed when bone metastases are suspected clinically; CT or MRI should be performed for positive bone scans and other suspicious or known metastasis lesions (such as the pelvis). The CT/MRI scan and other imaging results obtained before the subject signing the informed consent from can be used for tumor assessment in the screening period as long as they meet the requirements (and within 28 days before the first study drug is taken);

Record the concomitant medication: record the concomitant medication and concomitant treatment within 28 days before enrollment and during the study; once the subject has discontinued the study medication, only the concomitant medication and concomitant treatment used for the new or unresolved adverse events related to the study medication should be recorded;

Adverse event record: record from the day of signing the informed consent form.

The following screening procedures should be completed within 7 days before the start of study drug treatment:

ECOG performance status;

Vital signs examination: pulse, breathing rate, body temperature and blood pressure (blood pressure monitoring: blood pressure is monitored 3 times a day for 2 weeks before famitinib treatment, if blood pressure is abnormal, conduct daily follow-up; if blood pressure is normal, blood pressure will be monitored 2 times a week thereafter);

Physical examination: general condition, head and face, skin, lymph nodes, eyes, ear, nose, throat, oral cavity, respiratory system, cardiovascular system, abdomen, genitourinary system, musculoskeletal, neurological system and mental state, etc.;

Routine blood test: red blood cell count (RBC), hemoglobin (Hb), platelet count (PLT), white blood cell count (WBC), neutrophil count (ANC), lymphocyte counts;

Hematology: white blood cells, red blood cells, urine protein. If the urine protein is ≥2+, a 24-hour urine protein quantitative examination shall be added;

Fecal occult blood test (OB): The fecal occult blood test will be performed within 7 days before the first administration and at the end of the treatment visit. During the treatment period, the investigator will conduct fecal occult blood test based on the subject's relevant clinical tips (such as melena);

Blood chemistry test: alanine aminotransferase (ALT), aspartate aminotransferase (AST), glutamyl transpeptidase (γ-GT), total bilirubin (TBIL), direct bilirubin (DBIL), alkaline phosphatase (AKP), blood urea nitrogen (BUN) or urea (preferably blood urea nitrogen), total protein (TP), albumin (ALB), creatinine (Cr), blood glucose (GLU), K+, Na+, Ca2+, Mg2+ and Cl-;

Blood coagulation function: activated partial thromboplastin time (APTT), prothrombin time (PT), thrombin time (TT), fibrinogen (FIB), international normalized ratio (INR);

Thyroid function: serum thyroid stimulating hormone (TSH), free triiodothyronine (FT3), free thyroxine (FT4); if FT3 and FT4 are not available, they can be replaced by T3 and T4;

Tumor markers (CEA, CA153, CA125);

12-lead electrocardiogram: If there is any abnormality, perform other related examinations based on the investigator's judgement;

Echocardiography: Includes left ventricular ejection fraction (LVEF) assessment. Echocardiography examinations will be performed within 7 days before enrollment and at the time of treatment discontinuation/withdrawal, and examinations will be performed according to clinical prompts during the treatment;

Infectious disease screening;

Pregnancy test: All female subjects of childbearing age need to be tested;

6.2. Enrollment

Obtain written informed consent form signed by the subject;

Confirm the inclusion criteria and exclusion criteria;

Collect pregnancy test results;

For pre-administration laboratory tests (blood routine, urine routine, blood chemistry, coagulation function, and thyroid function) and electrocardiogram examinations, if the corresponding baseline laboratory examinations have been carried out within 7 days before administration, there is no need to repeat;

Administration.

After completing all screening and assessments, the subjects who are evaluated as qualified by the investigator will be given a number. After the number is obtained, the subjects will be treated with the study drug within 48 hours.

6.3. Treatment period

The treatment period starts from the first administration. The first study medication should be as close as possible to the time to complete the screening examinations and the time of confirmation that the subject meets the inclusion and exclusion criteria. Every 28 days is viewed as a treatment cycle.

For laboratory tests (blood routine, urine routine, blood chemistry, coagulation function, hormone test, and thyroid function) and electrocardiogram test, if the corresponding tests have been performed within 7 days before the administration, there is no need to repeat.

Before the start of a new treatment cycle, subjects should go to the clinical research center without eating breakfast and taking study drugs for corresponding examinations and assessments.

The following examinations should be completed within the window time listed in the test procedure. In case of legal holidays, the examinations can be performed in advance and the reasons for the over-window should be recorded in the CRF accordingly. The investigator can increase the examination items or enhance visit frequency according to the subjects' clinical conditions.

ECOG performance status: needs to be performed during follow-up in every treatment cycle follow-up;

Vital signs: needs to be performed during follow-up in every treatment cycle follow-up (if blood pressure is abnormal during famitinib treatment, follow-up and test daily; if blood pressure is normal, monitor blood pressure at least twice a week);

Physical examination: needs to be performed during follow-up in every treatment cycle follow-up;

Hematology: needs to be performed during follow-up in every treatment cycle follow-up, and nab-paclitaxel test needs to be performed before weekly chemotherapy (D1±1 day, D8±1 day, D15±1 day);

Urinalysis: needs to be performed during follow-up in every treatment cycle follow-up; if the test result shows urine protein ++ or above, add a 24-hour urine protein quantitative examination;

Fecal occult blood: needs to be performed during follow-up in every treatment cycle follow-up;

Blood chemistry: needs to be performed during follow-up in every treatment cycle follow-up; if necessary, myocardial enzyme spectrum examination can be carried out according to the condition of the subject; needs to be performed before weekly nab-paclitaxel chemotherapy (D1±1 day, D8± 1 day, D15±1 day); needs to be performed before every two weeks of camrelizumab treatment (D1±1 day, D15±1 day);

Coagulation function: needs to be performed during follow-up in every treatment cycle follow-up;

Thyroid function: needs to be performed during follow-up in every treatment cycle follow-up; additional examinations are performed when necessary, which is determined by the investigator according to the conditions of the subjects.

Tumor markers (CEA153, CA153, CA125): needs to be performed during follow-up in every treatment cycle follow-up;

Pregnancy test: performed if the subject is likely to become pregnant;

12-lead ECG: needs to be performed in every treatment cycle's follow-up; if necessary, the examination can be repeated (the interval time must be at least 10 minutes);

Echocardiography: needs to be performed during follow-up in every treatment cycle follow-up; if symptoms such as chest pain or palpitations occur during the study period, additional examinations can be performed when appropriate. The examination items are determined by the investigator according to the conditions of the subjects.

Tumor imaging examination: The time-point of tumor imaging examination during the administration period is determined after the start of the treatment (i.e., C1D1), regardless of the time during which the administration is suspended due to toxicity. The allowable window time for tumor imaging examination is ±7 days. Subjects who are assessed for CR or PR for the first time need to undergo imaging examination for confirmation 4 weeks later. The confirmed tumor assessment cannot change the previous fixed examination time-point. The specific time-points of assessment are as follows:

✔ For the trial period, once every 2 cycles (every 8 weeks) until the disease progression or the treatment is terminated and the subject withdraws from the study;

✔ If the disease progresses, the subject should terminate the trial and enter the follow-up period. Before the disease progresses, other anti-tumor treatments cannot be carried out;

✔ When withdrawal from the study is required due to the adverse event of intolerance or other reasons, a complete tumor imaging examination (unless performed within 28 days) is needed before the treatment is terminated.

Concomitant medication/treatment: record the concomitant medication/treatment information during the study at any time;

Adverse events: Observe and record adverse events during the study at any time.

PK blood sampling: SHR-1210: within 30 minutes before administration in each cycle from cycle 1 to cycle 4, and then within 30 minutes before administration every subsequent 4 cycles (12 weeks) and 28 days after treatment (±7 days), collect blood samples, separate serum, and examine SHR-1210 blood concentration and immunogenicity. Famitinib and nab-paclitaxel: within 30 minutes before all administration in the third week of the first cycle, within 5 minutes and 4 hours (±10 minutes) after the end of administration, collect blood samples, separate the plasma, and examine blood concentration of famitinib and paclitaxel respectively. Record in detail the administration time of famitinib the day before and on the day.

6.4. Follow-up period

Subjects will enter the follow-up period after terminating administration for any reason. The following follow-up is required until the completion of survival follow-up:

Safety follow-up: new adverse events within 28±7 days after the last administration should be recorded and followed up. For adverse events that have not recovered during the trial, follow-up and final assessment should be conducted. All adverse events have degraded to within grade 1, or the investigator believes that no further follow-up is necessary (such as clinically stable);

It is necessary to record in detail the adverse events, concomitant medication/treatment, whether to start other anti-tumor treatments, and unscheduled examinations during the period.

Efficacy follow-up: for subjects with non-imaging evidence of disease progression and non-death (such as intolerance or other conditions), the tumor imaging assessment will continue to be conducted at the time-point specified in the protocol since the last tumor imaging assessment during the study period, until the subject has PD, starts to use other anti-tumor drugs or dies (whichever comes first);

Record in detail relevant information such as the time of each follow-up, the results of tumor imaging assessments and other anti-tumor treatments during the period.

Survival follow-up: subjects who are excluded from the group due to PD or non-PD non-dead subjects are required to undergo survival follow-up after completing

safety follow-up or efficacy follow-up. Since the completion of safety follow-up and efficacy follow-up (whichever is completed later), the subject, his/her family or local physician must be interviewed by telephone (or clinical follow-up) at least once every 12 weeks (±7 days) until death, lost to follow-up or discontinuation of the trial (whichever comes first);

Collect survival information (date of death and cause of death) and information after the discontinuation of treatment (including anti-tumor treatments received), and each survival follow-up needs to be recorded in detail and filled in corresponding eCRF tables.

Subjects with non-imaging evidence of disease progression and non-death (such as intolerance or other conditions) must complete efficacy follow-up before receiving the survival follow-up.

6.5. Last visit

Complete the safety visit within 28 ± 7 days after the last administration, including ECOG performance status, vital signs, and physical examinations, and continue to monitor and record adverse events and concomitant medication information. If the subject starts to receive a new anti-tumor treatment within 28 days after the last administration, the visit should be completed before the new anti-tumor treatment.

6.6. Withdrawal from the study

Subjects who withdraw from the study due to the adverse event of intolerance or other reasons should complete all examinations required in the End of Treatment (EOT). It is also recommended that a complete tumor imaging examination be performed when the treatment is terminated (unless it has been performed within 28 days). After that, it is necessary to continue imaging examinations as possible until the disease progression/the patient dies/the patient receives new anti-tumor treatment (whichever occurs first). Subjects who are out of group due to reasons except disease progression will undergo tumor assessment every 3 cycles until disease progression, death, or the end of the study, and the time to progression or death, other tumor treatments, and SAE related to the study drug will be recorded.

6.7. Unscheduled visits

During the trial, if subjects need unscheduled follow-up due to adverse events, the following items should be recorded:

Concomitant medication/treatment;

Adverse events;

Relevant examinations performed (including imaging examinations, if any).

6.8. Rescue treatment/alternative treatment

Participation in the study is not the only option. The doctor will explain to you the available rescue/alternative treatments and discuss other possible treatment options. You can still choose to receive conventional chemotherapy recommended by clinicians, and you are free to

choose to participate in this study or other treatments.

7. Assessment

7.1. Efficacy assessment

7.1.1. Imaging assessment

7.1.1.1. Efficacy assessment in solid tumors

The efficacy assessment of this trial will adopt the Response Evaluation Criteria in Solid Tumors RECIST1.1, and the efficacy assessment will be performed on all patients who meet the inclusion criteria. The final results of ORR will be assessed by the investigator.

The efficacy in each checkpoint is divided into complete response (CR), partial response (PR), stable disease (SD) and progressive disease (PD) as per RECIST1.1. Please refer to appendix 3 for details.

This trial requires the patient to have at least one measurable lesion. The measurable lesion is defined as: neoplastic, non-lymphoid lesion with the longest diameter ≥ 10 mm, or tumor-related lymph node lesion with the longest and short diameter ≥ 15 mm. If the scanning slice thickness/slice distance exceeds 5 mm, the longest diameter of the measurable lesion is required to be twice the slice thickness/slice distance.

7.1.1.2. Requirements for medical imaging scanning

It is recommended to adopt qualified multi-slice spiral-enhanced CT for tumor imaging assessment. Subjects with a history of contrast media allergy should be treated according to the guidelines for the prevention of allergic reactions to contrast media in the trial center to perform enhanced CT as possible. If the subject is strictly contraindicated with contrast media, chest CT plain scan and abdominal MRI scan are allowed. CT or MRI is the only acceptable examination method for this test, and other imaging examination methods are used as supplementary imaging diagnostic methods. In principle, a same examination method is recommended for the baseline and follow-up.

During the screening period, at least CT or MRI of the chest, abdomen and brain should be performed. Bone scans are required when bone metastases are clinically suspected; CT or MRI should be performed for positive bone scans and other suspected or known metastasis lesions (such as the pelvis). If the bone X-ray examination has a good observation and assessment of this bone metastasis lesion, X-ray examination can also be used. After the start of the test, the same examination method is required in the specified visit window time. Imaging results such as CT/MRI scans obtained before the subject signing the informed consent form can be used for tumor assessment during the screening period if they meet the requirements of this test (and within 28 days prior to the first dosing of study drug).

The subsequent follow-up imaging assessment should be carried out under the same parameters and conditions as the baseline examinations (scanning layer thickness, use of contrast media, etc.), and it is performed once every 2 cycles (every 8 weeks ± 7 days) until the disease progression or the treatment is terminated and the subject withdraws from the study. The allowable window period for the imaging examination is ±7 days. Subjects who

are assessed for CR or PR for the first time need to be confirmed after 4 weeks. The imaging time is fixed since the study drug is taken for the first time, and the suspension of dosing does not change the assessment time. During the treatment and follow-up period, the investigator can also add scan sites for tumor assessment based on clinical indications. If there are indications of disease progression, an unscheduled visit can also be performed based on the condition of the disease. According to RECIST1.1, bone scan and PET are not suitable for evaluating the efficacy in target lesions. If necessary, if these tests are used to evaluate non-target lesions, the assessment frequency of these non-target lesions can be reduced. For example, the bone scan can be re-conducted only when the target lesion is confirmed to reach CR or when the bone lesion is suspected to be progressing.

In the subsequent follow-up tumor assessment, the same imaging techniques should be used for the same type of lesions as in the screening period. The assessment of anti-tumor activity will be carried out during the screening period and treatment through radiography according to the schedule of activities; the assessment should also be conducted when disease progression is suspected (for example, worsening symptoms) and the subject withdraws from treatment (if the assessment not completed in the previous 4 weeks).

Tumor assessments should be continuously conducted among all subjects in accordance with the research protocol, regardless of the interruption or delay of medication. If the subject needs to terminate the treatment because of general health deterioration, but there is no objective evidence of disease progression at this time, it should be reported as symptoms worsening. Even after the drug discontinuation, we must do our best to record the objective progression of the disease (such as confirmed by imaging examinations).

According to the requirements of RECIST1.1, there is more than one measurable lesion during baseline assessment. All lesions should be recorded and measured. The total number of lesions should not exceed 5 (not exceed 2 per organ). For example, if there are only one or two involved organs, patients can select at most 2 or 4 target lesions as baseline measurement lesions.

The target lesion must be selected based on the size (the longest diameter), which can represent all involved organs, and the measurement must be of good repeatability. When the largest lesion cannot be measured repeatedly, a repeatedly measurable largest lesion can be selected again.

Measurable lymph nodes must meet the following criteria: CT measured short diameter ≥ 15 mm. The baseline examination only needs to detect the short diameter. The short diameter of the nodule is usually used to determine whether the nodule has tumor metastasis. The size of the nodule is generally represented by the two-dimensional data of imaging examination (CT uses the axial plane, MRI chooses a plane from the axial plane, the sagittal plane or the coronal plane), and the minimum is the short diameter. For example, a 20 mm×30 mm abdominal nodule with a short diameter of 20 mm can be regarded as a malignant, measurable nodule. In this example, 20 mm is the measured value of the nodule. Nodules with a diameter ≥10 mm but ＜15 mm should not be regarded as target lesions; and nodules with a diameter less than 10 mm are not classified as pathological and do not need to be recorded and further observed.

The sum of the diameters of all target lesions (including the longest diameter of non-nodular lesions and the short diameter of nodular lesions) will be reported as the sum of the baseline diameters. If the lymph node diameter is included, as mentioned above, only the short diameter is counted. The sum of the baseline diameters will be used as a reference value for the baseline level of the disease.

All the other lesions, including pathological lymph nodes, can be regarded as non-target lesions and do not need to be measured. However, they should be recorded during baseline assessment, such as “existence”, “absence” or “clear disease progression” in rare cases. Extensive target lesions can be recorded along with target organs (such as extensive liver metastases).

7.1.2. Primary endpoint

Objective Response Rate (ORR): The proportion of subjects who received treatment and whose best overall response (BOR) was assessed as complete response (CR) or partial response (PR) according to RECIST1.1. The best overall response refers to the best efficacy assessed by the investigator. It is the best efficacy recorded during the period from the date of the first study dose to the date when the objective progression is recorded in accordance with RECIST 1.1 or to the date when the subsequent anti-tumor treatment is started (whichever occurs first). For subjects who have not recorded disease progression and have not started subsequent anti-tumor treatment, the best overall response will be determined based on all efficacy assessment results. Subjects who were assessed as CR or PR for the first time need to be confirmed 4 weeks later.

7.1.3. Secondary endpoint

Progression Free Survival (PFS): defined as the time from the date of the first study dose to the first recording of tumor progression (according to RECIST 1.1, regardless of whether the treatment is continued) or to the date of death due to any reason, whichever occurs first. For subjects who died but who had not previously reported disease progression, they were deemed to suffer disease progression on the date of death. Subjects with neither disease progression nor death will be censored on the date of their last evaluable tumor assessment. Subjects who did not have any tumor assessment during the study period and did not die will be censored on the date of randomization. Subjects who have not reported disease progression but started subsequent anti-tumor treatment will be censored for the last tumor assessment date that can be evaluated before the start of subsequent anti-tumor treatment.

Objective duration of response (Duration of Response, DoR): defined as the date from the first recording of tumor response (assessed according to RECIST 1.1) to the first recording of the objective progression of the tumor (assessed according to RECIST 1.1) or to the date of death for any reason, whichever occurs first. Subjects who have neither suffered disease progression nor died will be censored on the date of their last tumor assessment. Subjects who start receiving any subsequent anti-tumor treatment (except palliative radiotherapy for non-target bone lesions or central neurological system lesions during the treatment) and who have not reported disease progression in the past will be censored for the last tumor assessment date before the start of subsequent anti-tumor treatment.

Disease Control Rate (DCR): the proportion of subjects who received treatment and whose best overall response (BOR) was assessed as complete response (CR), partial response (PR) and stable disease (SD) ≥8 weeks according to RECIST1.1.

Overall Survival (OS): refers to the period from the date of the first study dose to the date of death for any reason. For subjects who were still alive in the last follow-up, their OS was counted as censored data based on the last follow-up time. For subjects who are lost to follow-up, their OS is counted as censored data based on the last confirmed survival time before the loss to follow-up. The OS for censored data is defined as the time from random grouping to censoring.

The above endpoints except OS are the efficacy assessment results during the trial period, and they are all evaluated according to RECIST 1.1. The analysis of OS includes the assessment results of tumor during the treatment and follow-up period.

7.2. Safety assessment

7.2.1. Pregnancy test

For fertile female subjects, blood HCG test will be performed within 7 days before administration. The examination must have a sensitivity of at least 25 mIU/mL for hCG. After obtaining a negative pregnancy test result during the screening period, appropriate contraceptive measures should be adopted. After that, a blood pregnancy test is required at the end of the administration visit. During the trial, whether to perform additional pregnancy tests can be determined according to the diagnosis and treatment routine of the trial center. If the hCG test is positive, the subject should terminate the administration and proceed according to section 11.3.

7.2.2. Adverse events

The assessment of adverse events (AE) includes type, incidence, severity (grading in accordance with NCI CTCAE version 5.0), time of occurrence and end, treatment measures, whether it is a serious adverse event and its relevance and outcome. AEs that occurred during the study, including signs and symptoms during the screening, will be recorded in the case report form in time.

7.2.3. Laboratory safety assessment

Laboratory examination samples will be collected according to the time-points specified in the “Schedule of Activities”. The following laboratory indicators are all sampled and tested by the relevant clinical research center. Considering the safety of subjects, unscheduled clinical laboratory examinations may be performed at any time.

Table 2: Laboratory examinations

Hematology	Blood chemistry test	Routine stool test	Urinalysis[a]
Hemoglobin<br>Red blood cell counts<br>White blood cell counts<br>Neutrophils counts<br>Lymphocyte counts<br>Platelet counts	Total bilirubin<br>Conjugated bilirubin<br>Unconjugated bilirubin<br>ALT<br>AST<br>Alkaline phosphatase<br>γ-GT<br>total protein<br>A/G<br>Urea/urea nitrogen<br>Creatinine<br>Uric Acid<br>Blood glucose<br>Triglycerides<br>Cholesterol<br>Potassium<br>Sodium<br>Chlorine<br>Calcium<br>Phosphorus<br>Magnesium	Stool occult blood	urinary protein<br>urine glucose<br>urine occult blood
**Infectious diseases screening**	**Coagulation function**	**Thyroid function**	**Others**
Five indicators of hepatitis B virus<br>HIV antibody<br>HCV antibody	PT<br>APTT<br>TT<br>Fbg<br>D-dimer	Free T3<br>Free T4<br>T3<br>T4<br>TSH	Pregnancy test[b]

Note:

a. If the protein ≥ 2+ by semi-quantitative methods (e.g., urine test paper), a 24-hour urine protein quantitative test shall be performed.

b. Women of childbearing age need to undergo blood HCG test during the screening period to exclude pregnancy, and urine HCG test can be performed at other time-points.

7.2.4. Vital signs and physical examination

Physical examination is performed by the study physician. The examination includes: general condition, skin and mucous, lymph nodes, head and neck, chest, abdomen, musculoskeletal, neural reflex, respiratory system, cardiovascular system, genitourinary system (if necessary), mental condition, etc. The weight should be measured each time the physical examination is performed, but the height can usually only be measured once during the screening period. If the investigator believes that the height of the subject may have changed, the height should be re-measured.

Vital signs examination includes the following items: body temperature, blood pressure, respiratory frequency and pulse.

The ECOG performance status is evaluated by the study physician in accordance with the "Physical Condition Scoring Criteria (ECOG)" in Appendix 1.

7.2.5. 12-Lead ECG

The 12-lead ECG will be performed by a qualified doctor at the time specified in the "Schedule of Activities".

All ECG examinations are required to be performed after the subjects have rest quietly in a side-lying position for at least 10 minutes, including at least: heart rate, QT, QTc and P-R time.

7.3. Pharmacokinetics and immunogenicity assessment

7.3.1. Pharmacokinetic and immunogenic blood sample collection

SHR-1210: From the 1st cycle to the 4th cycle, within 30 minutes before the administration of each cycle, then every 4 subsequent cycles (12 weeks), within 30 minutes before the administration, and 28 days (±7 days) after the treatment ends, collect blood samples and separate serum, examine the blood concentration and immunogenicity of SHR-1210.

Famitinib and nab-paclitaxel: within 30 minutes before all administration in the third week of the first cycle, within 5 minutes after the end of the administration, and 4 hours (±10 minutes), collect blood samples, separate the plasma, and examine the blood concentration of famitinib and paclitaxel respectively. To record the administration time of famitinib on the day before and on the day in details.

8. Dose adjustment

8.1. Overall regulations of dosage

When the hematological toxicity reaches grade III and above or the non-hematological toxicity reaches grade II and above, the investigator decides whether to suspend or reduce the dose; in the non-hematological toxicity, when controllable nausea, vomiting and fever with definite cause (such as infection, tumor, etc.) occur, active symptomatic treatment and treatment can be carried out without dose suspension or reduction.

Immune checkpoint inhibitors and chemotherapy and anti-angiogenesis targeted drugs have different mechanisms of action, and their toxicity profiles are quite different. There is currently no evidence that there will be significant interactions. Therefore, if the investigator judges that the toxicity is related to some drugs but not to other drugs, only the dose of drugs related to toxicity can be adjusted, and the treatment related to those drugs can be interrupted/delayed/terminated. If it is impossible to judge whether it is only related to some drugs, it is still necessary to adjust the medication of all study drugs.

The efficacy of anti-tumor drugs is continuous, and if the treatment is interrupted/delayed within the prescribed time limit, there is no evidence that the efficacy will be significantly reduced. Therefore, subjects can only interrupt/delay the treatment of some drugs related to

toxicity, and when the toxicity is basically recovered, all drug treatments are carried out as planned, to avoid disrupting the combination treatment regimen, and strive to give all drugs within the specified time window of each administration cycle treatment.

In general, each cycle can delay treatment in the beginning due to the unrecovered toxicity, but it is not allowed to delay the treatment beyond the treatment window time within each cycle. Every 4 weeks is viewed as a fixed treatment cycle. The interval between the administrations of paclitaxel is not allowed to be less than 7 days. If the eighth and fifteenth days of each cycle are delayed beyond the time window (plus 3 days) specified in the test flow chart, there will be no delay, and you can skip directly to the next treatment time-point.

The order of medication suspension is recommended to be nab-paclitaxel, famitinib and PD-1 to maintain the efficacy of anti-vascular targeted therapy and immunotherapy.

For situations that are not clearly specified in the protocol, the investigator needs to consider the subject's benefit/risk ratio before deciding. Once the dose is lowered, it cannot be enhanced back unless some drugs are discontinued.

If the discontinuation of nab-paclitaxel lasts over 6 weeks and still fails to meet the medication requirements of chemotherapy, the chemotherapy will be permanently discontinued. If the discontinuation of camrelizumab lasts over 12 weeks and still fails to meet the medication requirements, then it is permanently discontinued. If the discontinuation of famitinib lasts over 28 days and still fails to meet the medication requirements, then it is permanently discontinued.

8.1.1. Administration interruption and dose adjustment of camrelizumab

Camrelizumab is administered with a fixed dose, and it is not allowed to increase or reduce the dose of camrelizumab. If the subject delays or interrupts the treatment of camrelizumab due to toxicity, when the toxicity condition is improved within the prescribed time limit, the subject can continue to receive treatment at the original dose. It is allowed to delay/interrupt the administration for up to 12 weeks, calculated from the time of the last administration, otherwise the treatment will be terminated.

The time window for each administration is calculated from the date of the first administration in each cycle. The delay is generally no more than 3 days. If there is a delay (<3 days), the time window for the next administration will remain unchanged, and the time window for subsequent administration will still be 3 days. If the administration of D15 is delayed for more than 3 days, it is recommended that the dose should be not supplemented in this cycle, and should be continued in the next cycle, still subject to the time window calculated from the date of first administration.

Adverse events related to camrelizumab may be immunotoxicity, which may occur within a relatively short period of time after the first administration, or several months after the last administration. If the conditions listed in Table 3 below occur, camrelizumab treatment should be delayed or interrupted.

Table 3: SHR-1210 dose adjustment regulations due to immune-related toxicity

Camrelizumab-related	Grade of treatment	Time to restart	Permanent

toxicities	suspension (NCI CTC v5.0)	treatment	discontinuation
Diarrhea/ colitis	Grade 2-3	Toxicity reduced to grade 0-1	The toxicity does not reduce within 12 weeks after the last administration, or the corticosteroid dose cannot be reduced to 10 mg/day or lower dose of prednisone (or equivalent drug) within 12 weeks
	Grade 4	Permanent discontinuation	Permanent discontinuation
AST, ALT, or bilirubin elevation[a]	Grade 2	Toxicity reduced to grade 0-1	The toxicity does not reduce within 12 weeks after the last administration, or the corticosteroid dose cannot be reduced to 10 mg/day or lower dose of prednisone (or equivalent drug) within 12 weeks
	Grade 3-4	Permanent discontinuation[a]	Permanent discontinuation
Type I diabetes (if new) or hyperglycemia with signs of pancreatic β-cell failure	Grade 3-4 or new type I diabetes	When the subject's clinical and metabolic status is stable, camrelizumab treatment can be restarted.	
Hyperthyroidism	Grade 3	Toxicity reduced to grade 0-1	The toxicity does not reduce within 12 weeks after the last administration, or the corticosteroid dose cannot be reduced to 10 mg/day or lower dose of prednisone (or equivalent drug) within 12 weeks
	Grade 4	Permanent discontinuation	Permanent discontinuation
Hypothyroidism	Grade 2-4	After starting thyroid hormone replacement therapy, the treatment can be continued	
Pneumonia	Grade 2	Toxicity reduced to grade 0-1	The toxicity does not reduce within 12 weeks after the last administration, or the

			corticosteroid dose cannot be reduced to 10 mg/day or lower dose of prednisone (or equivalent drug) within 12 weeks
	Grade 3-4	Permanent discontinuation	Permanent discontinuation
Hypophysitis	Grade 2-4	Toxicity reduced to grade 0-1	The toxicity does not reduce within 12 weeks after the last administration, or the corticosteroid dose cannot be reduced to 10 mg/day or lower dose of prednisone (or equivalent drug) within 12 weeks
Infusion reaction	Grade 2[b]	Toxicity reduced to grade 0-1	If symptoms recur after adequate preventive medication, the medication will be permanently discontinued.
	Grade 3-4	Permanent discontinuation	Permanent discontinuation
Renal Failure/nephritis	Grade 2	Toxicity reduced to grade 0-1	The toxicity does not reduce within 12 weeks after the last administration, or the corticosteroid dose cannot be reduced to 10 mg/day or lower dose of prednisone (or equivalent drug) within 12 weeks
	Grade 3-4	Permanent discontinuation	Permanent discontinuation
Other drug-related toxicity	Grade 3	Toxicity reduced to grade 0-1	The toxicity does not reduce within 12 weeks after the last administration, or the corticosteroid dose cannot be reduced to 10 mg/day or lower dose of prednisone (or equivalent drug) within 12 weeks

	Grade 4	Permanent discontinuation	Permanent discontinuation

Note: If any serious or ≥ grade 3 (pneumonia ≥ grade 2) drug-related AEs occur again, or any life-threatening AEs, the drug should be permanently discontinued.

a. For subjects with liver metastases with grade 2 AST or ALT increased at baseline examinations, if the AST or ALT increased is ≥50% from baseline during the treatment period and continues for at least 1 week, the drug should be permanently discontinued.

b. If the symptoms are relieved within 1 hour after the temporary discontinuation of the drug, the infusion can be continued at 50% of the initial infusion rate. Otherwise, the medication cannot be restarted until the symptoms are completely relieved. The subject should be given adequate preventive medication when taking the next medication. For further treatment, please refer to Table 7 for recommendations for treatment of infusion reactions.

c. For subjects with intolerable or persistent grade 2 drug-related AEs, the investigator may suspend camrelizumab treatment at their discretion. For subjects with persistent grade 2 drug-related AEs, if the toxicity fails to be reduced to grade 0-1 after the last administration, the drug should be permanently discontinued.

Camrelizumab is already on the market, and other adverse reactions that occur during treatment should be dealt with reference to the latest instructions.

8.1.2. Administration interruption and dose adjustment of nab-paclitaxel

In general, before the D1 administration of each cycle, the absolute value of neutrophils must be ≥1500/μL and platelets must be ≥100000/μL. If the D1 of a cycle, during the treatment of camrelizumab and famitinib, the subject has not recovered from the hematological toxicity (the investigator judges that it is caused by nab-paclitaxel), and the nab-paclitaxel treatment is not concurrently administered, then it must be treated until the absolute value of neutrophils ≥1500/μL and platelets≥100000/μL, and subsequent D8 and D15 nab-paclitaxel treatments are not allowed to be conducted directly. During the treatment period, if the patient has severe neutropenia (ANC<500/μL for 1 week or more) or severe sensory neurotoxicity, the therapeutic dose of the subsequent treatment should be reduced to 75 mg/m^2. If the above-mentioned severe neutropenia or sensory neurotoxicity occurs again, the subsequent treatment dose should be reduced to 50 mg/m^2. Dose adjustments are not allowed for peripheral neurotoxicity of grade 1 or 2. Patients with sensory neurotoxicity of grade 3 should suspend the administration, and the treatment can be continued after the neurotoxicity is reduced to ≤ grade 2, and the dose should be reduced by a dose level in subsequent treatments.

The time window for each administration is calculated from the date of the first administration in each cycle. The delay is generally no more than 3 days. If there is a delay (<3 days), the time window for the next administration will remain unchanged, and the time window for subsequent administration will still be 3 days. If the dose of D15 is delayed for more than 3 days, it is recommended that the dose should be not supplemented in this cycle, and the drug should be continued in the next cycle, still subject to the time window calculated

from the date of the first administration.

nab-paclitaxel is already on the market, and other adverse reactions that may occur during treatment should be dealt with reference to the latest instructions.

8.1.3. Administration adjustment of famitinib

Dose adjustments due to famitinib-related toxicity include: administration suspension (up to 28 days), dose reduction (according to the initial dose of apatinib used, it can be reduced to 15 mg qd) and permanent discontinuation.

When hematological toxicity grade ≥ 3 or non-hematological toxicity grade ≥ 2, administration delay and dose reduction are required; For non-hematological toxicity, controllable nausea, vomiting and fever with definite cause (below 38°C), active symptomatic treatment can be carried out first, with no need for immediate administration delay or dose reduction.

When famitinib-related toxic reactions occur, the administration should be delayed until the subject recovers from the toxicity, and the original dose should be continued or reduced by a dose level. The minimum dose level can be reduced to 15 mg/d, if so, the patient is still unable to tolerate, and famitinib should be permanently discontinued. During the study period, the dose of famitinib is not allowed to be increased.

9. Concomitant treatment

For patients without high-risk infectious factors or bleeding risks, it is recommended to give colony stimulating factor or interleukin-11 or thrombopoietin therapy only when bone marrow suppression of grade 3 or above occurs. The investigator has the right to decide whether it needs to be dealt with accordingly.

The patient can receive the best supportive care. During treatment, patients can receive bisphosphonates for bone metastases. The clinical combined diseases and various AEs should be actively handled, such as rash, diarrhea, hypertension, hand-foot syndrome, liver and kidney damage, stomatitis, non-infectious pneumonia, and infusion reaction, etc., symptomatic treatment should be carried out according to the judgment of the clinicians. All drugs used in combination should be recorded in the case report form (CRF) in strict accordance with GCP regulations.

This protocol prohibits the use of NMPA approved modern Chinese medicine formulations and immunomodulators (such as thymosin, interferon, interleukin-2 and lentinan, etc.) for the treatment of breast cancer.

10. Treatment of common adverse reactions

10.1. Treatment of hand-foot syndrome

Hand-foot syndrome (HFSR): a skin toxicity, it is a kind of palm-plantar sensation or erythema.

When it occurs, the reactions in compression or stress area are more obvious. It can appear among cancer patients in the process of receiving chemotherapy or molecular targeted therapy. The characteristics of HFSR are numbness, dullness, paresthesia, tingling, no pain or pain, skin swelling or erythema, desquamation, chapped, indurated blisters or severe pain.

Grading:

Grade 1: Numbness/hypesthesia/paresthesia, painless swelling or erythema, and/or discomfort that does not affect normal activities of the hands and/or feet.

Grade 2: Painful erythema, swelling, and/or discomfort affecting the patient's daily activities of the hands and/or feet.

Grade 3: Wet desquamation, ulcers, blisters, or severe pain and/or severe discomfort that prevents the patient from working or performing daily activities of hands and/or feet. The pain is intense and the skin function is lost, which are relatively rare.

Symptomatic treatment:

Take some necessary supportive treatments including: strengthening skin care, keeping the skin clean, avoiding secondary infections; avoiding pressure or friction; using moisturizers or lubricants, and topical use of emulsions or lubricants containing urea and corticosteroids; if necessary, use antifungal or antibiotic topically.

Note: If the hand-foot syndrome of grade 2 or above occurs 3 consecutive times, and there is

a tendency to aggravate, the medication shall be terminated and the subject shall withdraw from the clinical trial.

10.2. Treatment of hepatic toxicity

Transaminase and total bilirubin (TBIL) increased may indicate drug-induced liver injury (DILI). The threshold of potential DILI may depend on the patient's baseline values of AST/ALT and TBIL. Medical review is required to ensure that the increase in liver-related examination values is not caused by cholestasis, which is defined as: for patients without bone metastasis, ALP increases > 2×ULN, with R value <2, or for patients with bone metastasis, hepatic ALP increases. The R value is calculated by dividing ALT by ALP, using the ULN multiple of the two values for calculation. It indicates that the relative increase pattern of ALT and/or ALP is due to cholestasis or liver cell damage or mixed damage.

For patients without cholestasis, treatment should be discontinued immediately, and liver function (LFT) should be re-examined as soon as possible, preferably within 48 hours of the discovery of abnormal results. The assessment should include laboratory examinations, detailed medical history, physical examinations and the possibility of liver metastasis or new liver disease, obstruction/compression, etc.

Hepatotoxicity monitoring includes the following liver function tests: albumin, ALT, AST, total bilirubin, direct and indirect bilirubin, alkaline phosphatase (if alkaline phosphatase elevates to grade 2 or above, then test the type of alkaline phosphatase), creatine kinase, and prothrombin time (PT) or INR and γ-glutamyl transferase. For patients with Gilbert syndrome: total bilirubin and direct bilirubin must be monitored, and monitoring should be strengthened only when direct bilirubin changes.

If AST, ALT, and/or bilirubin elevate and medication needs to be discontinued, close observation is recommended, including:

Re-examine liver enzyme and serum bilirubin, 2 to 3 times a week. If the abnormal results are stable or return to normal values, the frequency of re-examinations should be reduced to 1 or less per week.

Obtain a more detailed medical history of current symptoms.

Obtain a more detailed past medical history and/or concomitant medical history, including any pre-existing liver disease history or risk factors.

Obtain a history of concomitant medications (including over-the-counter drugs, traditional Chinese medicines, and dietary supplements), alcohol consumption, recreational drugs, and special diets.

Exclude acute viral hepatitis A, B, C, D and E; hepatotropic virus infection (CMV, EBV or HSV); autoimmune or alcoholic hepatitis; NASH; hypoxic/ischemic liver disease; and biliary tract disease.

Obtain a history of exposure to environmental chemicals.

Obtain the results of other tests to assess liver function, if applicable (e.g., INR,

direct bilirubin).

Consider a gastrointestinal or liver disease consultation.

10.3. Treatment of hypertension

Targeted drug therapy causes hypertension: angiogenesis inhibitors such as bevacizumab, sorafenib, sunitinib, etc., target the VEGF signaling pathway, which can cause hypertension or aggravate the original hypertension. The key mechanism of action may be： the decrease of NO/PGI2 secretion by endothelial cells/platelets, abnormal blood vessel density (small blood vessels and capillaries), vascular stiffness, and endothelin dysfunction. Among them, sunitinib can also cause a decrease in left ventricular ejection fraction (LVEF).

Monitoring and treatment of this type of hypertension: blood pressure monitoring should be performed weekly during the first 6 weeks of targeted drug therapy. Once hypertension occurs, the following standard treatment drugs can be given: angiotensin II receptor blockers, β-receptor blockers, diuretics, ACEI, etc. or a combination therapy on the above-mentioned drugs.

Reference for the treatment of hypertension caused by famitinib: Hypertension after Sorafenib treatment generally occurs in 1 to 2 weeks after treatment, and concomitant medication can be adopted continuously, and this kind of hypertension can usually be controlled through conventional antihypertensive treatment. For increased blood pressure that is difficult to control, it can generally be relieved by reducing the dose of targeted drugs or drug discontinuation.

The optimized selection of drugs for hypertension caused by targeted therapy (non-hepatic metabolism):

- Valsartan (Diovan) 80-320 mg qd;
- Atenolol (Tenormine) 50-100 mg qd;
- Losartan potassium/hydrochlorothiazide tablets (Hyzaar) 12.5-100 mg qd;
- Telmisartan (Mecaxine) 20-80 mg qd;
- For those whose blood pressure is difficult to control: Amlodipine (Luohuoxi) 2.5-10 mg qd.

10.4. Treatment of proteinuria

For patients with two consecutive test results of urine protein++, a 24-hour urine protein quantification examination is required.

Note: In case of nephrotic syndrome, the trial administration shall be terminated and the subject shall withdraw from the clinical trial.

10.5. Guidelines for supportive treatment of immuno-oncology drug Camrelizumab

Subjects should receive appropriate supportive treatment measures deemed necessary by the investigator. Supportive treatment measures for the treatment of potentially immunologically

related AEs are listed below, including oral or intravenous use of corticosteroids, and the use of other anti-inflammatory drugs when there is no improvement of symptoms after corticosteroid use. Steroid reduction may take multiple cycles, as symptoms may worsen during the reduction process. Other reasons that may require other supportive treatment should be excluded as much as possible, such as metastatic disease or bacterial or viral infection. When the investigator confirms that the AE is related to SHR1210, the supportive treatment measures listed below can be adopted, otherwise they are not needed.

If capillary hyperplasia occurs, biopsy and pathological examination should be performed as much as possible. For subjects with severer capillary hyperplasia or longer duration, endoscopy and MRI are recommended to confirm whether the visceral mucosa is involved.

Symptoms and signs including diarrhea/colitis enterocolitis (such as diarrhea, abdominal pain, hematochezia or mucous stool, with or without fever) and bowel perforation (such as peritonitis and intestinal obstruction) should be carefully monitored. All subjects with diarrhea/colitis should be advised to drink enough fluids. If enough liquid cannot be taken orally, fluids and electrolytes should be infused intravenously. For patients with Grade ≥ 2 diarrhea, GI consultation and endoscopy should be adopted to diagnose or rule out colitis; for patients with Grade 2 diarrhea/colitis, corticosteroids should be taken orally; for patients with Grade 3 or 4 diarrhea/colitis, steroids should be given intravenously, followed by oral high-dose steroids; after the symptoms have improved to Grade 1 or below, the dose of steroids should be reduced for no less than 4 weeks.

For patients with grade 2 AEs such as elevated AST, ALT, or bilirubin, intravenous or oral corticosteroids should be given, and liver function should be monitored more frequently until it returns to baseline (test once a week); for patients with Grade 3 - 4 AEs, 24 - 48 hours of intravenous corticosteroid treatment should be adopted; after symptoms have improved to Grade 1 or below, the dose of steroids should be reduced for no less than 4 weeks.

Hyperthyroidism/hypothyroidism may occur at any time during treatment, and patients are monitored for changes in thyroid function (monitor regularly at the beginning of and during treatment) as well as for clinical symptoms and signs of thyroid disease. For Grade 2 hyperthyroidism, non-selective β-blockers (such as propranolol) are recommended in initial treatment; for Grade 3 - 4 hyperthyroidism, corticosteroids should be given intravenously at the beginning and then orally. After the symptoms have improved to Grade 1 or below, the dose of steroids should be reduced for at least 4 weeks. In the process of steroid reduction, appropriate hormone replacement therapy may be adopted; for Grade 2 - 4 hypothyroidism, thyroid hormone replacement therapy (such as levothyroxine) may be adopted.

Patients with Grade 2 pneumonia should be given systemic corticosteroid therapy. After the symptoms have improved to Grade 1 or below, the dose of steroids should be reduced for no less than 4 weeks; if steroids are given for a long time, antibiotics should be used prophylactically.

For Grade 2 immune-related hypophysis, corticosteroid therapy should be continued. After symptoms have improved to Grade 1 or below, the dose of steroids should be reduced for no less than 4 weeks. In the process of steroid reduction, appropriate hormone replacement

therapy may be adopted; for patients with Grade 3 or 4 hypophysis, intravenous corticosteroids should be given at the beginning, followed by oral corticosteroids. After the symptoms have improved to Grade 1 or below, the dose of steroids should be reduced for no less than 4 weeks. During the steroid reduction process, appropriate hormone replacement therapy may be adopted.

For patients with Grade 2 AEs such as renal failure or nephritis, corticosteroids should be given; for Grade 3 - 4 AEs, patients should be given systemic corticosteroid therapy; after symptoms have improved to Grade 1 or below, the dose of steroids should be reduced for no less than 4 weeks.

10.6. Infusion Reaction Management

Infusion of immunologic agent camrelizumab may cause related infusion reactions. Once they occur, follow Table 4 for reaction grading and treatment management.

Table 4: Classification and treatment management of infusion reactions

CTCAE Grade	Clinical Symptoms	Clinical Treatment	Camrelizumab Treatment
Grade 1	Mild transient reaction.	Bedside observation and close monitoring until recovery.<br>Preventive medication before infusion recommended to be administered in the future: diphenhydramine<br>50 mg, or equivalent and/or acetaminophen<br>325-1000 mg, at least 30 minutes before medication.	Continued.
Grade 2	Moderate reaction, requiring treatment or suspension of medication, which can quickly relieve symptoms after treatment (such as antihistamines, non-steroidal anti-inflammatory drugs, anesthetics, bronchodilators, intravenous infusions, etc.).	Intravenous infusion of normal saline, diphenhydramine 50 mg IV or equivalent and/or acetaminophen<br>325-1000 mg;<br>bedside observation and close monitoring until recovery.<br>According to clinical conditions, corticosteroids or bronchodilators can be used;<br>the amount of study drug infused are documented in original medical records;	Suspended. When the medication is restarted after the symptoms disappear, the infusion rate should be 50% of the initial infusion rate. If there are no complications within 30 minutes, the infusion rate can be increased to 100% of the initial infusion rate. Monitor patients closely. If the symptoms reoccur, no more infusion of camrelizumab should be used for the current treatment.

		Preventive medication before infusion recommended to be given in the future: diphenhydramine 50 mg, or equivalent and/or acetaminophen 325-1000 mg are administered at least 30 minutes before the medication. If necessary, cortisol hormone (equivalent to 25 mg hydrocortisone) is used.	
Grade ≥ 3	Grade 3: Serious reaction. No rapid relief after treatment and/or suspension of medication; or recurrence of symptoms after symptom relief; occurrence of sequelae that require hospitalization.<br>Grade 4: Life-threatening	Stop the infusion of SHR1210 immediately;<br>Begin intravenous infusion of normal saline.<br>• Bronchodilator is recommended; subcutaneous injection of 1:1000 epinephrine solution<br>0.2-1 mg, or slow intravenous injection of 1:10000 solution of adrenaline 0.1-0.25 mg is adopted; and/or diphenhydramine 50 mg plus methylprednisolone 100 mg or equivalent intravenous injection are adopted if necessary;<br>•Follow the guidelines of research institutions for the treatment of allergic reactions;<br>Bedside observation and close monitoring until recovery.	Drug discontinuation.

11. Adverse event report

11.1. Adverse events (AE)

11.1.1. Definition of AE

AE refers to any untoward medical occurrence in a study subject administered an

investigational product which does not necessarily have a causal relationship with the treatment. AEs that occur from the time of signing the informed consent form until 28 days after the last medication were collected during the study. AE can be any unfavorable and unintended symptoms, signs, abnormal laboratory findings or diseases, etc., including at least the following conditions:

1) Aggravation of existing (prior to enrollment of study) medical condition/disease (including exacerbation of symptoms, signs, and laboratory abnormalities);

2) Any new occurrence of AEs: any adverse medical conditions that newly occur (including symptoms, signs, newly diagnosed diseases);

3) Abnormal laboratory test findings with clinical significance.

The investigator should record any AEs of the subject in detail, including the name of the AE, the description of all related symptoms, time of occurrence, severity, correlation with the study drug, duration, measures taken, results and prognosis.

11.1.2. Criteria for the severity of AEs

Refer to NCI-CTC AE V5.0 for the classification of AEs. If there is an AE that is not listed in the NCI-CTCAE V5.0, please refer to the following table 5:

Table 5: Classification criteria of the severity of AEs

Grades	Clinical descriptions of severity.
Grade1	Mild; events cause clinical symptoms or mild clinical symptoms; events cause only clinical or laboratory abnormalities; intervention not indicated.
Grade2	Moderate; minimal, local or non-invasive treatment; discomfort caused by AEs is enough to interfere with age-appropriate activities of daily living (ADL) of the subject including cooking, shopping, using phone, counting money, etc.
Grade3	Severe or medically significant but not immediately life-threatening; hospitalization or prolongation of hospitalization indicated; disabling; limiting self-care ADL. Self-care ADL refer to bathing, dressing, undressing, eating, going to the bathroom, taking medicine, etc., without being bedridden.
Grade 4	Events are life-threatening; events require urgent treatment.
Grade 5	Death related to AE.

11.1.3. Criteria for the relationship between AE and study drug

All AEs are collected and recorded from the day the informed consent form is signed until 28 days after the last use of the study drug, regardless of the relation between the AE and the study drug, and regardless of whether the AE is from the treatment group and whether the investigational product is administered or not. Any subjective discomforts of subjects or abnormal changes in laboratory test indexes during treatment should be documented truthfully, and the severity, duration, treatment measures and prognosis of the AE should be recorded at the same time. Extensive factors should be taken into consideration by the investigator to comprehensively assess relationship between the AE and the study drug such as the time sequence between the occurrence of AE and medication, characteristics and toxicological and

pharmacological effects of the study drug, concomitant drugs used on the subject, as well as the subject's underlying disease, medical history, family history, and challenge and re-challenge reaction. The possible relationship between the AE and the study drug is assessed according to the five-level classification of "definitely related, possibly related, possibly unrelated, definitely unrelated, and unable to determine".

Table 6: Classification of the relationship between AE and study drugs

Grades	Criteria
Definitely related	There is a plausible time relationship between the event and the medication, and the event conforms to the known reactions of the suspected drug;<br>symptoms improve after stopping the drug, and the event recurs after administration is restarted.
Possibly related	There is a plausible time relationship between the event and the medication, and the event does not conform to the known reactions of the suspected drug; the clinical state of patients or other treatment may also be responsible for the event.
Possibly unrelated	There is no plausible time relationship between the event and the medication, and the event does not conform to the known reactions of the suspected drug; the clinical state of patients or other treatment may also be responsible for the event.
Definitely unrelated	There is no plausible time relationship between the event and the medication, and the event does not conform to the known reactions of the suspected drug. The clinical state of patients or other treatment may also be responsible for the event. The event is eliminated after symptoms improve or other treatment is terminated, and the event recurs after administration is restarted.
Unable to determine	There is no clear time relationship between the event and the medication. The event is similar to the known reactions of the drug. Other drugs used at the same time may also cause corresponding events.

11.2. Serious adverse events (SAE)

11.2.1. Definition of SAE

SAE refers to any medical events during clinical trials including inpatient hospitalization or prolongation of existing hospitalization, disability, disruption of work ability, life-threatening event or death, and congenital malformation. The aforementioned medical events include:

Events leading to death;

Life-threatening events (defined as events in which subjects are at the risk of immediate death);

Events that require hospitalization or prolonged hospitalization;

Events that can cause permanent or severe disability/dysfunction/disruption of work

ability;

Events that can cause congenital anomaly or birth defects;

Other important medical events (defined as events that harm the subject or events that require intervention to prevent any of the above from happening).

11.2.2. Hospitalization

Adverse events in clinical studies that lead to hospitalization (even if less than 24 hours) or prolongation of existing hospitalization should be considered as SAEs.

Hospitalization does not include the following:

Rehabilitation institutions

Nursing home

Admission to the routine emergency room

Same-day surgery (such as outpatient/same-day/ambulatory surgery)

Social factors (medical insurance reimbursement, etc.)

Hospitalization or prolongation of hospitalization in the absence of a precipitating clinical AE is not in itself an SAE. For example:

Admission for treatment of a preexisting condition not associated with the development of a new AE or with a worsening of the preexisting condition (for example, to check the laboratory abnormalities that persist until the study);

Administrative admission (such as, annual routine physical examination);

Protocol-specific admission during a study (such as procedure required by the protocol);

Optional admission not associated with a precipitating clinical AE (such as elective surgery);

Scheduled treatment or surgery should be recorded in the entire protocol and/or the subject’s individual baseline data;

Admission exclusively for the administration of blood products.

Diagnostic or therapeutic invasive (such as surgery) and non-invasive operations should not be reported as AEs unless the symptoms leading to these operations meet the definition of AEs. For example, an acute appendicitis that begins during the reporting period should be reported if the AE requirements are met, and the resulting appendectomy should be recorded as treatment of the AE .

11.2.3. Disease progression and death

Disease progression is defined as that the disease under study is progressing or worsening, including radiological progression and progression of clinical symptoms and signs. New

metastases relative to the primary tumor or the progression of the original metastases are both considered to be disease progression. Events caused by symptoms and signs of disease progression should not be reported as SAEs, which include events that pose a threat to life, require inpatient hospitalization or prolongation of existing hospitalization, and cause permanent or severe disability/dysfunction/disruption of work ability and congenital abnormalities or birth defects. Deaths due to symptoms and signs of disease progression should be reported as SAEs.

Death of subject during the study must be reported as SAE, regardless of whether the subject has received new anti-tumor therapy. The term “death” should not be reported as an AE or SAE term, but rather as an outcome of an event. The event that causes or leads to death should be recorded as an AE or SAE. If the cause of death is unknown and cannot be determined at the time of reporting, the AE or SAE term should be reported as “unknown cause of death”.

11.2.4. Other anti-tumor treatment

SAEs are recorded from the time of subject signing the informed consent form to the end of the safety follow-up (28 days after the last use of the study drug). If a subject begins a new anticancer therapy during the safety follow-up period, the reporting period for non-fatal SAEs ends at the time the new treatment is started, unless the SAE is suspected to be related to the investigational product. Death must be reported as an SAE if it occurs during the safety reporting period, irrespective of any intervening treatment.

11.2.5. SAE reporting system

SAEs should be collected from the subject signing the informed consent to form the end of the safety follow-up. Any SAE that occurs should be immediately recorded in the Serious Adverse Event Report Form by investigator whether it is the first report or follow-up report. The form must include the signature and date of the investigator and must be timely reported to relevant department in accordance with legal requirements.

SAEs suspected to be related to the study drug that occur after the safety follow-up should be collected and recorded in detail, including symptoms, severity, causality with investigational product, time of onset, time of treatment, action taken with investigational product, follow-up time and methods as well as outcome. If the investigator considers that an SAE is not related to investigational product, while potentially related to study conditions (such as discontinuation of the original treatment, or complications during the trial), the relationship should be specified in the narrative section of the SAE report form. If the intensity of an ongoing SAE or its relationship with the investigational product changes, a follow-up report should be submitted immediately. If the investigator believes that there is misinformation in the previously submitted SAE report form, correction, cancellation or degradation can be made in the follow-up report, and then be reported in accordance with the SAE reporting procedure.

11.3. Pregnancy

If a female subject is pregnant during the clinical trial, the subject will withdraw from the trial.

The investigator should report the pregnancy event to the Ethics Committee once informed.

The investigator should follow up on the pregnancy outcome until 1 month after delivery.

Pregnancy results including stillbirth, spontaneous abortion, and fetal malformation are considered as SAEs and should be reported in accordance with the time limit for SAE reporting.

SAE experienced by the subject during pregnancy should be recorded in the Serious Adverse Event Report Form and reported according to the SAE reporting procedure.

11.4 Follow-up of AE/SAE

All AEs/SAEs should be followed up until the safety follow-up is ended or AEs/SAEs disappear, relieve to baseline or Grade ≤ 1, or AEs/SAEs reach a stable state, or AEs/SAEs are explained reasonably (such as lost to follow-up, death).

The investigator should inquire the subject at each visit about the AE/SAE that occurred since the last visit. At the end of the study, the principles of collection and follow-up of AEs/SAEs after the subject's last medication can be referred to Table 7 below:

Table 7: AE/SAE collection and follow-up principles

Grades	Collection/Recording Requirements	follow-up Requirements
No drug-related AE	Until the end of the safety follow-up or the beginning of a new anti-tumor treatment (whichever comes first)	By the end of the safety follow-up
Drug-related AE	By the end of the safety follow-up	Follow-up until AEs disappear, relieve to baseline or Grade ≤ 1, or reach a stable state, or AEs are explained reasonably (such as lost to follow-up, death).
No drug-related SAE	Until the end of the safety follow-up or the beginning of a new anti-tumor treatment (whichever comes first)	By the end of the safety follow-up
Drug-related SAE	Indefinite period	Follow-up until SAEs disappear, relieve to baseline or Grade ≤ 1, or reach a stable state, or SAEs are explained reasonably (such as lost to follow-up, death).

AE: adverse event; SAE: serious adverse event

12. Data analysis/statistical methods

12.1 Sample size

Assuming that the point estimation of ORR in the treatment group is 0.6, 41 subjects are needed when the width of the two-sided 95% confidence interval is 0.3. Considering the drop-out rate of 10%, therefore, a total of 46 subjects are needed.

12.2 Statistical analysis plan

In this study, SAS version 9.4 or above software is used for data processing and analysis.

Categorical data will be descriptively summarized using statistics including frequency and percentage, as well as the 95% confidence interval when necessary. Continuous data will be descriptively summarized using statistics including the mean, standard deviation (SD), median, minimum and maximum.

The detailed analysis plan and methods will be described in the Statistical Analysis Plan (SAP).

12.3 Statistical assumptions and discriminant rules

Not applicable.

12.4 Analyzed population

Full Analysis Set (FAS): It refers to subjects who have taken the study drug at least once. The full analysis set is the key analysis set for the efficacy analysis of this study;

Per-Protocol Analysis Set (PPS): A subset of subjects defined in the FAS; subjects with major protocol violation and major impact on the analysis results are excluded;

Safety Analysis Set (SS): It refers to subjects who have taken the study drug at least once;

Pharmacokinetic Analysis Set (PKS): It refers to subjects who have used the study drug and whose pharmacokinetics data can be evaluated after the medication;

Immunogenicity Analysis Set: It refers to subjects who have used the study drug and whose immunogenicity data can be evaluated after the medication.

12.5. Statistical methods

The following subsections should include a description of the planned statistical methods.

12.5.1 Basic methods

This trial is a single-arm, open-label, phase 2 clinical trial.

12.5.2 Primary efficacy endpoint analysis

The analysis of the primary endpoint is based on the FAS. The primary endpoint analysis will estimate the objective response rate (ORR) assessed by the investigator and its 95%

confidence interval (Clopper-Pearson method).

Objective response rate (ORR): It refers to the proportion of subjects whose best overall response (BOR) is complete response (CR) or partial response (PR) when the clinical curative effect of tumor is assessed according to RECIST 1.1.

The specific analysis method of the PPS is the same as that of the FAS.

12.5.3 Secondary efficacy endpoint analysis

The secondary endpoint analysis will include DCR, PFS, DoR and OS.

It will estimate the disease control rate (DCR) assessed by the investigator and its 95% confidence interval (Clopper-Pearson method).

The Kaplan-Meier method is used to estimate the progress-free survival (PFS), duration of response (DoR), and overall survival (OS) and calculate the corresponding 95% confidence intervals (Brookmeyer-Crowley method based on log-log transformation with standard errors calculated using Greenwood formula).

12.5.4 Processing of missing data

In this study, the missing data of efficacy endpoints require no special processing, and the missing values are not estimated in the safety assessment.

12.5.5 Safety analysis

The analysis is based on the SS analysis set.

AEs that occur during the study will be coded using the MedDRA dictionary. The frequency and incidence of AEs will be described in terms of system organs and preferred terms, and the correlation and severity of AEs should be further described in tabular form. The AEs, adverse reactions, AEs leading to withdrawal of the study, the incidence of AEs and SAEs leading to death should be summarized; Severity of AEs and adverse reactions should be determined as follows: for subject to which the same AE repeatedly occur, the most serious one is included in the analysis; for subject to which different AEs occur, the most severe AE is included in the analysis.

Laboratory test indexes: Descriptive statistics are performed for laboratory indicators.

Vital signs: Mean, maximum, minimum, median, and standard deviation are used to describe the measured values and value changes in each visit.

Descriptive analysis is performed on physical examination, 12-lead ECG, etc.

The baseline is defined as the latest test data before the first administration.

12.5.6 Pharmacokinetic analysis

Descriptive statistical summary analysis is performed on the blood concentrations of SHR-1210, famitinib, and paclitaxel.

A population pharmacokinetic model is used to study the pharmacokinetic characteristics of

SHR-1210, famitinib and paclitaxel.

12.5.7 Immunogenicity analysis

The positive rate of anti-SHR-1210 antibodies is calculated, and the antibody levels of positive subjects are described in tabular form.

13. Data processing

13.1 Requirements for data filled by investigators

1) For all patients who have signed the informed consent form and been screened to enter the study, all items in the case report form must be recorded carefully in detail, and no blank or missing items shall be allowed (blank spaces without records shall be underlined);

2) All data in the case report form shall be checked with the medical records of subjects to ensure correctness;

3) As the original data, the case report form can only be underlined when any correction is made, and the revised data should be noted aside with the investigator's signature and date;

4) The copy of test report shall be pasted at the specified area of the test report attached to the case report form;

5) Significantly high data or data outside the scope of clinical acceptance should be verified and explained as necessary by the investigator;

6) Please refer to the pathology report form for instructions.

13.2 Traceability of data, filling of Case Report Form (CRF)

As the original records of study, the medical records should be stored properly. The case report form is obtained from the medical records and filled in by the investigator for each enrolled case.

14. Quality assurance and quality control

The study site will be regularly monitored and visited during the study to ensure the implementation of the protocol. The original data shall be reviewed to ensure the consistency of the data on the case report form.

15. Ethics

This study must be carried out in strict accordance with the requirements of the SFDA *Good Clinical Practice* and *Declaration of Helsinki*.

Independent ethics committee (IEC)

The protocol, informed consent form, and other information provided to the subjects must be reviewed and approved by IEC before the clinical trial.

Informed consent form (ICF)

The investigator is responsible for informing each subject of the purpose, process and

potential risks of the study in both oral and written form before they are enrolled in the study, and subjects should be informed of the right to decide whether to participate in the study and to withdraw study at any time. The subject or his legal representative should sign the informed consent form after reading and understanding it, and keep the copy of the signature page.

16. Study progress, data storage

16.1 Study progress

Duration of enrollment: 6 months (from August 2019 to January 2020).

Follow-up after treatment: 12 months after the last subject is enrolled.

Key analysis date: January 2021.

16.2 Data storage

The case report form should be signed and reviewed by the principal investigator. All case report forms, detailed data of typical cases, and clinical study drug use record forms should be preserved properly after the study is completed. Investigators should keep all original data of subjects, imaging data of CR or PR patients, original signed informed consent form, CRF copy, and drug distribution records.

17. Reference

1. 2018 NCCN Guidelines Breast Cancer Version 1.2018-March,2018

2. Breast Cancer Professional Committee of China Anti-Cancer Association, 2017 Edition of Guidelines and Norms for Diagnosis and Treatment of Breast Cancer by China Anti-Cancer Association. [J] China Oncology, 2017, 21(9): 695-760

3. Yi-Zhou Jiang, Ding Ma, Chen Suo,et al. Genomic and Transcriptomic Landscape of Triple-Negative Breast Cancers: Subtypes and Treatment Strategies [J]. Cancer Cell, 2019, 35:1-13.

4. Nanda R, Chow LQ, Dees EC et al. Pembrolizumab in Patients with Advanced Triple-Negative Breast Cancer: Phase Ib KEYNOTE-012 Study. J Clin Oncol 2016; 34: 2460-2467. 5. Jun Cao, Jian Zhang, Zhonghua Wang, et al. Hypothyroidism as a potential biomarker of efficacy of famitinib, a novel VEGFR-2 inhibitor in metastatic breast cancer[J]. Cancer Chemother Pharmacol, 2014, 10.1007/s00280-014-2505-x.

6. Schmid P, Adams S, Rugo H S, et al. Atezolizumab and nab-paclitaxel in advanced triple-negative breast cancer[J]. New England Journal of Medicine, 2018, 379(22): 2108-2121.

7. Schmitt naegel M, Rigamonti N, Kadioglu E et al. Dual angiopoietin-2 and VEGFA inhibition elicits antitumor immunity that is enhanced by PD-1 checkpoint blockade. Sci Transl Med 2017; 9.

8. Socinski M A, Jotte R M, Cappuzzo F, et al. Atezolizumab for first-line treatment of metastatic nonsquamous NSCLC[J]. New England Journal of Medicine, 2018, 378(24): 2288-2301.

Appendix 1: Physical Condition Scoring Criteria (ECOG, Eastern Cooperative Oncology Group)

Activity score	Description
0	Asymptomatic, fully active, and able to carry out unlimited activities.
1	Symptomatic, fully able to walk, but limited in heavy physical activity, able to perform light or sedentary tasks, such as light housework and office work.
2	Symptomatic, able to walk, able to perform self-care ADL, but unable to engage in any physical activity, and remain awake more than 50% of a day (day time in bed <50%).
3	Symptomatic, limited self-care ability, time in bed or chair >50% of waking hours, but not yet being bedridden.
4	Completely loss of function, completely unable to perform self-care ADL, and being bedridden.
5	Dead.

Appendix 2: Clinical Staging Criteria for Breast Cancer (AJCC breast cancer TNM staging)

Stage 0	TisN0M0
Stage I	T1N0M0
Stage IIA	T0N1M0
	T1N1M0
	T2N0M0
Stage IIB	T2N1M0
	T3N0M0
Stage IIIA	T0N2M0
	T1N2M0
	T2N2M0
	T3N1, 2M0
Stage IIIB	T4N0M0, T3N1M0, T4N2M0
Stage IIIC	Any T, N3M0
Stage IV	Any T, any N, M1

Appendix 3: Response Evaluation Criteria in Solid Tumors

The Response Evaluation Criteria in Solid Tumors V1.1 (excerpt)

(New Response Evaluation Criteria in Solid Tumors: Revised RECIST Version 1.1)

1. Background

Omitted intentionally.

2. Purpose

Omitted intentionally.

3. Measurability of the tumor at baseline

3.1 Definition

Tumor lesions/lymph nodes at the baseline can be classified as measurable and non-measurable according to the following definitions:

3.1.1 Measurable lesions

Tumor lesions: must be accurately measured in at least one dimension (longest diameter in the plane of measurement is to be recorded) with a minimum size of :

10 mm by CT scan (CT scan slice thickness no greater than 5mm)

Clinical routine examination instrument 10 mm (tumor lesions that cannot be accurately measured with a diameter measuring instrument should be recorded as non-measurable)

Chest X-ray 20 mm

Malignant lymph nodes: pathologically enlarged and measurable; the short diameter of a single lymph node must be ≥15 mm (the CT slice thickness is recommended not to exceed 5 mm). At baseline during follow-up, only the short diameter is measured and followed up.

3.1.2 Non-measurable Lesions

Other lesions include small lesions (maximum diameter <10 mm or short diameter of pathological lymph node ≥10 mm to <15 mm) and unmeasurable lesions. Unmeasured lesions include meningeal disease, ascites, pleural or pericardial effusion, inflammatory breast cancer, skin/lung cancerous lymphangitis, abdominal masses that cannot be diagnosed and followed up by imaging, and cystic lesions.

3.1.3 Special considerations regarding lesion measurement

Bone lesions, cystic lesions, and lesions previously treated with local therapy should be particularly noted:

Bone lesions:

Bone scan, PET scan or plain film are not suitable for bone lesions measurement,

but can be used to confirm the existence or disappearance of bone lesions;

Lytic lesions or mixed lytic/osteoblastic lesions with identifiable soft tissue that can be assessed by tomographic techniques such as CT or MRI can be considered as measurable if the soft tissue meets the definition of measurability described above;

Osteogenic lesions are non-measurable lesions.

Cystic lesions:

Lesions that meet the criteria for radiographically defined simple cysts are not considered as measurable or non-measurable malignant lesions;

If cystic metastatic lesions are present and meet the definition of measurability described above, they can be considered as measurable lesions. However, if non-cystic lesions are present in the same patient, they should be preferred as target lesions.

Locally treated lesions:

Lesions situated in a previously irradiated area, or in an area previously subjected to other local-regional therapy are generally considered non-measurable unless there is unequivocal progress of this lesion. The protocol should describe in detail the conditions under which these lesions are considered measurable.

3.2 Description of measurement method

3.2.1 Lesion measurement

All tumors should be measured in metric for clinical assessment. All baseline of tumor size should be assessed as close as possible before the start of treatment, which must be done within 28 days (4 weeks) prior to the start of treatment.

3.2.2 Assessment method

The same techniques and methods should be adopted for baseline assessment and subsequent measurement of lesions. Except for lesions that can only be evaluated by clinical examination, all lesions must be evaluated by imaging.

Clinical lesions: Clinical lesions can only be considered as measurable (such as skin nodules, etc.) when they are superficial and have a diameter of ≥10 mm. For patients with skin lesions, it is recommended that color photo with a ruler should be taken to measure the size of the lesion. For lesions both imaging and clinical examination are applicable to, imaging assessment should be adopted whenever possible since it is more objective and can be reviewed repeatedly at the end of the study.

Chest X-ray: When tumor progression is considered as an important study endpoint, priority should be given to chest CT because CT is more sensitive than X-ray, especially for new lesions. Chest X-ray examination is only applicable when the measured lesion has a clear boundary and the lung is well ventilated.

CT, MRI: CT is currently the best available and reproducible method for efficacy assessment. Measurability of lesions in this guideline is defined on the basis of CT slice thickness ≤ 5 mm.

If CT slice thickness is greater than 5 mm, the minimum size for a measurable lesion should be twice the slice thickness. MRI is also acceptable in certain situations (e.g., for body scans).

Ultrasound: Ultrasound should not be used to measure the size of the lesion. Ultrasonography, due to its operational dependency, is not reproducible for measurements and cannot guarantee the identity of techniques and results between different measurements. If new lesions are identified by ultrasound in the course of the trial, CT or MRI should be used for confirmation. If there is concern about radiation exposure at CT, MRI may be used instead.

Endoscopy, laparoscopy: It is not recommended to use these techniques for objective assessment of tumors, but endoscopy and laparoscopy are useful to confirm CR when biopsies are obtained or to confirm relapse in trials where the endpoint of the study is recurrence following CR or surgical resection.

Tumor markers: Tumor markers alone cannot be used to evaluate objective tumor response. However, if the marker level exceeds the upper limit of normal at baseline, it must return to normal when used to evaluate complete response. The fact that tumor markers vary from disease to disease should be taken into consideration when measurement criteria is included into the protocol. Specific criteria for CA-125 response (recurrent ovarian cancer) and PSA (recurrent prostate cancer) response have been published. In addition, the Gynecologic Cancer Intergroup has developed CA-125 progress criteria which are to be integrated with objective tumor assessment for first-line treatment protocol of ovarian cancer.

Cytology/histology techniques: These techniques can be used to identify PR and CR (e.g., residual benign tumor tissues in lesions of germ cell tumors) under specific conditions specified in the protocol. When effusions are known to be a potential adverse effect of treatment (such as treatment with taxane compounds or angiogenesis inhibitors) and the measurable tumor meets the criteria for disease response or stabilization, tumor-related effusion that appears or worsens during treatment can be diagnosed by cytological techniques to distinguish disease response (or stable disease) and disease progression.

4. Tumor response evaluation

4.1 Evaluation of all tumors and measurable lesions

To evaluate the objective response or future progress, it is necessary to estimate the overall tumor burden at baseline, as a reference for the subsequent measurements. Only patients with measurable lesions at baseline should be included in the study where objective response is the primary endpoint. Measurable lesions are defined by the presence of at least one measurable lesion. For studies with disease progression (time to progression or grade of progression on a fixed date) as the primary endpoint, the inclusion criteria for the protocol must specify whether it is limited to patients with measurable lesions.

4.2 Baseline records of target and non-target lesions

When more than one measurable lesion is present at baseline, all lesions up to a maximum of five (no more than 2 per organ) should be recorded and measured as target lesions representing all involved organs (this is, at most two or four target lesions can be used for baseline measurement among patients with one or two involved organs).

The target lesion must be selected based on its size (the maximum diameter) and the reproducibility of measurement, representing all involved organs. When the largest lesion cannot be measured repeatedly, another largest lesion which can be measured repeatedly can be selected.

Lymph nodes need special attention because they are normal tissues and can be detected by imaging even without tumor metastasis. Short diameter must be no less than 15mm for pathological lymph nodes defined as measurable nodules or target lesions. Only short diameter needs to be detected in measurement at baseline. The short diameter of a nodule is normally used by radiologists to judge whether a nodule has metastatic disease. Nodule size is generally represented by the two-dimensional data of the image detection (for CT, use the axial plane; for MRI, select a plane from the axial plane, sagittal plane or coronal plane). The minimum value is the short diameter. For example, a 20 mm × 30 mm abdominal nodule with a short diameter of 20 mm can be regarded as a malignant, measurable nodule. Here 20 mm is the measured value of the nodule. Nodules with a diameter of ≥10 mm but <15 mm should not be regarded as target lesions. Nodules with a diameter of <10 mm are not included in the category of pathological nodules, which need no records and further observations.

The sum of the diameters of all target lesions (including the maximum diameter of non-nodular lesions and the short diameter of nodular lesions) will be reported as the sum of the baseline diameters. If lymph nodes are to be included in the sum, only the short diameter will be included as noted above. The sum of baseline diameters will be used as reference of baseline disease.

Other lesions, including pathological lymph nodes, which require no measurement, should be identified as non-target lesions and recorded at baseline (such as “existence”, “absence” or rarely “unequivocal progression”). Extensive target lesions can be recorded together with target organs (such as pelvic lymph nodes amplification or large-scale liver metastases).

4.3 Response criteria

4.3.1 Assessment of target lesions

Complete response (CR): All target lesions disappear, and the short diameter of all pathological lymph nodes (including target and non-target nodules) must be reduced to <10 mm.

Partial response (PR): The sum of diameters of target lesions is reduced by at least 30% from the baseline.

Disease progression (PD): The sum of diameters of target lesions is increased by at least 20%, compared to the minimum sum in the whole study (if the baseline value is the smallest, use it as the reference). In addition, the absolute value of the sum of diameters must be increased by at least 5 mm (the appearance of one or more new lesions is also considered disease progression).

Stable disease (SD): If target lesions dose not relive to PR or deteriorate to disease progression, the minimum sum of diameters can be used as a reference in the study.

4.3.2 Precautions for target lesion assessment

Lymph nodes: Even if the diameter of lymph nodes identified as target lesions is reduced to less than 10 mm, the actual short diameter compared to the baseline value must be recorded for each measurement (consistent with the anatomical plane at the baseline). That is, if the lymph node is a target lesion, the lesion cannot be said to have disappeared completely even if it reaches the criterion of complete response, as the short diameter of a normal lymph node is defined as <10 mm. Case report forms or other data collection methods may therefore be designed to have target nodal lesions recorded in a separate section: for CR, the short diameter of all lymph nodes must be less than 10 mm; for PR, SD and PD, the actual value of the short diameter of the target lymph node is to be included in the sum of diameters of target lesions.

Target lesions that are too small to be measured: In clinical studies, all lesions (nodules or non-nodules) recorded at baseline should be measured and recorded again in subsequent assessments, even if the lesions are very small (e.g. 2 mm). However, sometimes lesions are so small that they become much faint on CT scan and it is difficult for radiologists to define the exact diameter, then the lesions may be reported as being “too small to measure”. When this happens, it is still important to record a value on the CRF. If the radiologist believes that the lesion may have disappeared, the diameter of it should be recorded as 0 mm. If the lesion does exist but is rather vague, and the diameter cannot be measured accurately, it should be recorded as the default value of 5 mm. (Note: It is unlikely to happen to lymph nodes because they generally have a measurable size under normal conditions, or are often surrounded by fat as they are in the retroperitoneum; but if this happens, a default value of 5 mm should be recorded). The default value of 5 mm is derived from the cutting thickness of the CT scan which does not change with different cutting thicknesses of CT. Since there is little chance that the same result will recur, it is suggested that the default value will reduce the risk of erroneous assessment. But it needs to be reiterated that if the radiologist can provide the exact diameter of the lesion, the actual value must be recorded, even if it is below 5 mm.

Separate or combined lesions: When a non-nodular lesion splits into fragments, the maximum diameters of separated parts should be added together for the sum of the diameters of the lesions. Similarly, combined lesions can be separated according to the planes between them, and then the maximum diameter of each part can be calculated. However, if the union is inseparable, the maximum diameter should be recorded as the maximum diameter of the entire fusion lesion.

4.3.3 Assessment of non-target lesion

This part defines the response criteria for non-target tumors. While some non-target lesions can actually be measured, there is no need for measurement, and only qualitative assessment is needed at the time-points specified by the protocol.

Complete response (CR): All non-target lesions disappear, and tumor markers return to normal levels. All lymph nodes are of non-pathological size (short diameter <10 mm).

Incomplete response/non-disease progress: The presence of one or more non-target lesions and/or the persistent presence of tumor markers exceeding normal levels.

Disease progression: There is unequivocal progression of existing non-target lesions. Note: The appearance of one or more new lesions is also considered disease progression.

4.3.4 Special considerations regarding the assessment of the progression of non-target lesions

The supplementary explanation of the definition of the progression of non-target lesions is as follows. When patients have measurable non-target lesions, the lesions can be evaluated as unequivocal progression only when the non-target lesions have deteriorated to the point where treatment must be terminated, even if the target lesions are assessed as stable or partial response. However, the general increase in the size of one or more non-target lesions is not sufficient to qualify for progression. Therefore, when the target lesion become stable or partial response, it is almost impossible to define the overall tumor progression only by the change of the non-target lesion.

When the patient's non-target lesions are not measurable: This situation occurs in some phase 3 trials when the inclusion criteria don't stipulate that there must be a measurable lesion. The overall assessment also refers to the above criteria. However, because there is no measurable data of the lesion in this case, it is difficult to evaluate the deterioration of non-target lesions (by definition: all non-target lesions must be unmeasurable). Therefore, when changes in non-target lesions lead to an increase in the overall disease burden comparable to the disease progression of the target lesions, it is necessary to establish an effective test method for lesion assessment based on the definition of unequivocal progression of non-target lesions. As described, an increase in tumor burden is equivalent to an additional 73% increase in volume (equivalent to 20% increase in the diameter of a measurable lesion). Another example is the change in peritoneal effusion from "minor" to "large"; lymphatic disease from "local" to "widespread"; or described in the protocol as "worsen enough for treatment changes". Examples include pleural effusions ranging from trace amount to large amount, lymphatic involvement spreading from the primary site to a distance, or may be described in the protocol as "necessary for treatment changes" If unequivocal progression is found, the patient should be considered to have had overall disease progression at that time-point. It is preferable to have criteria that can be applied to the assessment of non-measurable lesions. It should be noted that the added criteria must be reliable.

4.3.5 New lesions

The appearance of new malignant lesions indicates the progression of the disease; therefore, assessments of new lesions are very important. There are currently no specific criteria for imaging detection of lesions, but the discovery of a new lesion is certain. For example, the progression cannot be attributed to different imaging techniques, changes in imaging morphology, or other lesions other than tumors (such as, some so-called new bone lesions are only the cure of the original lesion, or the recurrence of the original lesion), which is very important when a patient's baseline lesions show partial or complete response. For example, a necrosis of a liver lesion may be classified as a new cystic lesion on the CT report, while it is not in fact.

The lesions that have been detected during follow-up but not found in the baseline

examination will be regarded as new lesions indicating disease progression. For example, for a patient who is found to have visceral lesions in the baseline examination and have metastases during CT or MRI head examination, the intracranial metastases will be regarded as the evidence of disease progression, even if no head examination has been done in the baseline examination.

If a new lesion is equivocal, for example because of its small size, further treatment and follow-up assessment are required to confirm whether it is a new lesion. If repeated examinations confirm there is definitely a new lesion, the disease progression should be counted from the time of its initial discovery.

FDG-PET assessment of lesions generally requires additional testing for confirmation. It is reasonable to combine the results of FDG-PET inspection and additional CT inspection to evaluate the disease progression (especially for new suspicious diseases). New lesions can be confirmed by FDG-PET examination, according to the following procedures:

Disease progression is indicated if the baseline FDG-PET test result is negative, and the FDG-PET test at the next follow-up is positive.

No baseline FDG-PET examination is performed, and subsequent FDG-PET examination result is positive:

If the positive result of FDG-PET examination at the follow-up is consistent with that of the CT examination, disease progression is indicated.

If the positive result of FDG-PET examination is not consistent with the CT examination result, another CT examination is required for confirmation (if confirmed, the time to progression should be counted from the discovery of abnormality in the previous FDG-PET examination).

If the positive result of FDG-PET examination is consistent with that of the CT examination, but the lesion does not show progression by imaging tests, then there is no disease progression.

4.4 Assessment of best overall efficacy

The best overall efficacy is the best efficacy recorded from the beginning of the study to the end of the study, and any necessary conditions must be taken into consideration for confirmation. Sometimes the efficacy appears after the end of the treatment, so the protocol should clarify whether the assessment after the treatment is concluded in the best overall efficacy assessment. The protocol must specify influences of any new treatments before progression on the best efficacy. The best efficacy of the patient is mainly dependent on results of target and non-target lesions and conditions of new lesions, as well as on the nature of the study, protocol requirements, and criteria for results measurement. Specifically, in non-randomized trials where efficacy is the primary endpoint, confirmation of PR or CR is necessary to evaluate the best overall efficacy.

4.4.1 Time-point response

It is assumed that at each protocol-specified time-point a response occurs. Table 1 provides a

summary of the overall efficacy at each time-point for patients who have measurable disease at baseline.

Table 1: Time-point response: subjects with target lesions (including or excluding non-target lesions)

Target lesion	Non-target lesion	New lesion	Overall efficacy
CR	CR	None	CR
CR	Non-CR/non-PD	None	PR
CR	Unable to be evaluated	None	PR
PR	Non-progressive or unable to be fully evaluated	None	PR
SD	Non-progressive or unable to be fully evaluated	None	SD
Unable to be fully evaluated	Non-progressive	None	NE
PD	Any cases	Yes or no	PD
Any cases	PD	Yes or no	PD
Any cases	Any cases	Yes	PD
CR = complete response, PR = partial response, SD = stable disease, PD = disease progression, NE = not assessable.			

If the patient has no measurable lesions (no target lesions), refer to Table 2 for assessment.

Table 2: Time-point response-subjects with only non-target lesions

Non-target lesion	New lesion	Overall response
CR	None	CR
Non-CR/non-PD	None	Non-CR/non-PDa
Unable to be fully evaluated	None	Unable to be evaluated
Unclear PD	Yes or no	PD
Any cases	Yes	PD
a: For non-target lesions, “Non-CR/Non-PD” refers to better response than SD. As SD is increasingly used as an endpoint for evaluating efficacy, non-CR/non-PD efficacy has been developed for cases where no lesions have been specified for measurement.		

4.4.2 Description of missing and unevaluable assessment

If imaging or measurement of the lesion cannot be performed at a particular point in time, the patient cannot be evaluated at that point in time. If only part of the lesions can be evaluated in an assessment, the lesions are usually regarded as unable to be evaluated at that time-point, unless there is evidence that the missing lesions will not affect the efficacy assessment at the specified time-point. This is likely to occur in the case of disease progression. For example, if a patient has 3 lesions with a total diameter of 50 mm at baseline, only 2 lesions of which are evaluable with a total diameter of 80 mm, the patient will be evaluated as disease progression, regardless of the impact of the missing lesions.

4.4.3 Best overall response: all time-points

The best overall response is determined once all the data of the patient is available.

Assessment of the best overall response when the confirmation of the complete or partial response is not required in the study: The best response in the study refers to the best response of all time-points. For example, the response of a patient is evaluated as SD in the first cycle, PR in the second cycle, and PD in the last cycle, but the best overall response is evaluated as PR. The best overall response evaluated as SD must meet the protocol-specified minimum time from baseline. If the minimum time standard is not met, the best overall response cannot not be evaluated as SD, which instead dependence on further assessments. For instance, if a patient is evaluated as SD in the first cycle and PD in the second cycle, but does not meet the minimum time requirement for SD, the best overall response should be evaluated as PD. The same patient lost to follow-up after being evaluated as SD in the first cycle will be considered unevaluable.

Assessment of the best overall response when complete or partial response needs to be confirmed in the study: A complete or partial response can be declared only if each subject meets the criteria for a partial or complete response specified in the study and if response is confirmed at a subsequent time-point as specified in the protocol (generally 4 weeks later). In this case, the description of best overall response is shown in Table 3.

Table 3: The best overall response to be confirmed for CR and PR

Overall response at the first time-point	Overall response at subsequent time-points	Best overall response
CR	CR	CR
CR	PR	SD, PD or PR[a]
CR	SD	If SD lasts for enough time, it is SD, otherwise it should be PD
CR	PD	If SD lasts for enough time, it is SD, otherwise it should be PD
CR	NE	If SD lasts for enough time, it is SD, otherwise it should be NE
PR	CR	PR
PR	PR	PR
PR	SD	SD
PR	PD	If SD lasts for enough time, it is SD, otherwise it should be PD
PR	NE	If SD lasts for enough time, it is SD, otherwise it should be NE
NE	NE	NE
CR = complete response, PR = partial response, SD = stable disease, PD = disease progression, NE = not assessable.<br>a: If there is CR at the first time-point, and any disease at a subsequent time-point, the response assessment will still be PD at the subsequent time-point even if the response of the subject reaches the PR standard compared to the baseline (because the disease will reappear after CR). The best response		

depends on whether SD occurs within the shortest treatment interval. However, sometimes the first assessment is CR, but the subsequent scans still suggest small lesions. Thus, in fact the response assessment should be PR instead of CR at the first time-point. In this case, the first assessment as CR should be modified to PR, and the best response should be PR.

4.4.4 Special tips for response assessment

When a nodular lesion is included in the total target lesion assessment, and the size of the nodule is reduced to "normal" (<10 mm), there will still be a scan report of the lesion. To avoid overestimation based on the increase in the size of the nodule, the measurement result will be recorded even if the nodule is normal. As mentioned earlier, this means that subjects with complete response will not be recorded as 0 on the CRF.

If response confirmation is required during the study, repeated "unmeasurable" time-points will complicate the best response assessment. The analysis plan of the study must show that these missing data/assessments can be explained in response assessment. For example, in most studies, the PR-NE-PR response of a subject can be regarded as a confirmation of response.

A symptomatic progression should be reported when a subject's overall health condition deteriorates and requires discontinuation of medication with no evidence to support it. Every effort should be made to evaluate objective progression even after treatment discontinuation. Symptomatic deterioration is not a description of a response but the reason for stopping treatment. The objective response of subjects with symptomatic deterioration will be assessed through the target and non-target lesions shown in Tables 1 to 3.

Early progression, early death and non-evaluability are special cases of the study and should be clearly described in each protocol (depending on the treatment interval and treatment period).

In some cases, it is difficult to distinguish local lesions from normal tissues. When the assessment of complete response depends on this definition, it is recommended that biopsy be performed prior to the assessment of local disease. FDG-PET may be used to confirm the complete response in a manner similar to a biopsy when the abnormal imaging test results of some subjects' local lesions are considered to represent fibrosis or scar formation. In this case, the application of FDG-PET should be described prospectively in the plan, and the report of the specialist medical literature for this situation should be used as support. However, it must be noted that the limitations of FDG-PET and the biopsy itself (including the resolution and sensitivity of both) will lead to false positive results in the complete response assessment.

For equivocal findings of progression (e.g., very small and uncertain new lesions; cystic changes or necrosis in existing lesions), treatment may continue until the next assessment. If disease progression is confirmed in the next assessment, the date of progression should be recorded as the earlier date when progression is suspected for the first time.

4.5 Frequency of tumor re-assessment

The frequency of tumor re-assessment during treatment depends on the treatment regimen, which should be consistent with the type and schedule of treatment. However, in phase 2 trial

where the beneficial effect of treatment is not known, follow-up every 6 to 8 weeks (at the end of a cycle) is reasonable, and the length of the time interval may be adjusted in special regimens or situations. The protocol should specify which tissue sites need to be evaluated at the baseline (usually those most likely to be closely related to the metastasis of the tumor studied) and the frequency of assessment repetitions. Normally, both target and non-target lesions should be assessed at each assessment. In optional cases, some non-target lesions may be evaluated less frequently. For example, repeated bone scans may be required when the target disease is confirmed as CR or when there is suspicion of skeletal disease progress.

Tumor re-assessment after treatment depends on whether the response rate or the time-point of a certain event (progress/death) is regarded as the clinical endpoint. If the time-point of a certain event (such as TTP/DFS/PFS) is selected as the endpoint, the routine repeated assessments specified in the protocol need to be carried out. The scheduled assessment, especially in randomized comparative trials, should be listed in the timetable (e.g. 6~8 weeks during treatment, or 3~4 months after treatment), and should not be affected by other factors, such as treatment delay, dosing interval and any other events that may cause imbalance in the treatment arm in the timing of disease assessment.

4.6 Response assessment/confirmation of response period

4.6.1 Confirmation

For non-randomized clinical trials with response as the primary endpoint, PR and CR must be confirmed to ensure responses are not evaluated by error. This also allows for a sound interpretation of results in the context of available historical data where response should also have been confirmed. Yet in all other cases including randomized clinical trials (Phase 2 or 3) or studies with stable disease or disease progress as the primary endpoint, response confirmation is no longer necessary because it is of no value for the interpretation of trial results. However, without the requirement for response confirmation, central review to prevent bias is even more important, especially in non-blinded studies.

In the case of SD, measurements must meet the SD criteria specified in the protocol at least once within the shortest time interval after the start of the trial (generally no less than 6-8 weeks).

4.6.2 Overall response stage

The overall response stage starts from the measurement of the first CR or PR (whichever is measured first) to the time when the disease recurrence or progression is recorded for the first time (the minimum value recorded in the study is used as a reference for disease progression). The total complete response starts from the measurement of the first CR to the time when the disease recurrence or progression is recorded for the first time

4.6.3 Stable disease stage

The stable disease stage starts from the beginning of treatment to disease progression (in randomized trials, starting from the time of randomization); with the minimum sum in the trial as a reference (the baseline sum is used as a reference for PD calculation if it is the smallest). The clinical relevance of stable disease varies between studies and diseases. If the

proportion of patients who maintain the minimum stable stage is used as the endpoint in a study, the protocol should specify the minimum time interval between two measurements in the SD definition.

Note: The response stage, stable stage, and PFS are affected by the frequency of follow-up after baseline assessment. It is not within the scope of this guideline to define a standard follow-up frequency. The frequency of follow-up is dependent on many factors, such as the type and stage of the disease, the treatment period, and standard norms. However, the limitations in the accuracy of the endpoints should be considered if comparisons between trials are needed.

4.7 PFS/TTP

4.7.1 Phase 2 clinical trials

This guideline mainly focuses on the application of objective response as an endpoint in phase 2 clinical trials. In some cases, response rate may not be the best choice for evaluating the potential anticancer activity of new drugs/new regimens, where the PFS/PPF at demarcated time-point can be considered as a suitable surrogate indicator to provide the original signal of the biological activity of the new drug. However, these assessments will be obviously questioned in an uncontrolled trial because seemingly valuable observations may be related to biological factors such as patient screening rather than the results of drug intervention. Therefore, phase 2 clinical trials with objective response as study endpoints are best designed with randomized controlled groups. But non-randomized trials are justified for certain tumors with consistent clinical performance (often poor). In these cases, careful documentation of efficacy is required when assessing the expected PFS or PPF due to the absence of positive controls.

Appendix 4: Classification criteria for AE of drug (NCI-CTCAE Version 5.0)

Table 4: Classification criteria of the severity of AEs

Grades	Clinical description of severity.
Grade1	Mild; events cause clinical symptoms or mild clinical symptoms; events cause only clinical or laboratory abnormalities; events require no treatment.
Grade2	Moderate; events require minor, topical or non-invasive treatment; discomfort caused by AEs is enough to interfere with age-appropriate Activities of Daily Living (ADL) of the subject including cooking, shopping, using phone, counting money, etc.
Grade3	Events cause severe illness or medically serious symptoms but temporarily not life-threatening; events require hospitalization or an extended length of hospital stay; events cause disabilities; events interrupt a subject's self-care ADL including bathing, dressing, undressing, eating, going to the bathroom, taking medicine, etc., without being bedridden.
Grade 4	Events are life-threatening; events require urgent treatment.
Grade 5	Events cause death.
